# A novel prognostic model for papillary thyroid cancer based on epithelial–mesenchymal transition‐related genes

**DOI:** 10.1002/cam4.4836

**Published:** 2022-05-24

**Authors:** Rui Liu, Zhen Cao, Meng Pan, Mengwei Wu, Xiaobin Li, Hongwei Yuan, Ziwen Liu

**Affiliations:** ^1^ Department of General Surgery, Peking Union Medical College Hospital Chinese Academy of Medical Sciences and Peking Union Medical College Beijing People's Republic of China; ^2^ State Key Laboratory of Medical Molecular Biology & Department of Immunology Institute of Basic Medical Sciences Chinese Academy of Medical Sciences, School of Basic Medicine Peking Union Medical College Beijing People's Republic of China

**Keywords:** bioinformatics, epithelial–mesenchymal transition, nomogram, papillary thyroid cancer, predictive model, recurrence, The Cancer Genome Atlas

## Abstract

**Background:**

The frequent incidence of postsurgical recurrence issues in papillary thyroid cancer (PTC) patients is a primary concern considering the low cancer‐related mortality. Previous studies have demonstrated that epithelial–mesenchymal transition (EMT) activation is closely related to PTC progression and invasion. In this study, we aimed to develop a novel EMT signature and ancillary nomogram to improve personalized prediction of progression‐free interval (PFI).

**Methods:**

First, we carried out a differential analysis of PTC samples and pairwise normal thyroid samples to explore the differentially expressed genes (DEGs). The intersection of the DEGs with EMT‐related genes (ERGs) were identified as differentially expressed EMT‐related genes (DE‐ERGs). We determined PFI‐related DE‐ERGs by Cox regression analysis and then established a novel gene classifier by LASSO regression analysis. We validated the signature in external datasets and in multiple cell lines. Further, we used uni‐ and multivariate analyses to identify independent prognostic characters.

**Results:**

We identified 244 prognosis‐related DE‐ERGs. The 244 DE‐ERGs were associated with several pivotal oncogenic processes. We also constructed a novel 10‐gene signature and relevant prognostic model for recurrence prediction of PTC. The 10‐gene signature had a C‐index of 0.723 and the relevant nomogram had a C‐index of 0.776. The efficacy of the signature and nomogram was satisfying and closely correlated with relevant clinical parameters. Furthermore, the signature also had a unique potential in differentiating anaplastic thyroid cancer (ATC) samples.

**Conclusions:**

The novel EMT signature and nomogram are useful and convenient for personalized management for thyroid cancer.

## Graphical abstract

We e identified a 10‐EMTgene signature and constructed a prognostic nomogram with relevant clinical parameters involved. The novel 10‐gene classifier and the nomogram could provide a novel and reliable tool for prognosis prediction with PTC, therefore help decision making for the purpose of individualized surveillance.

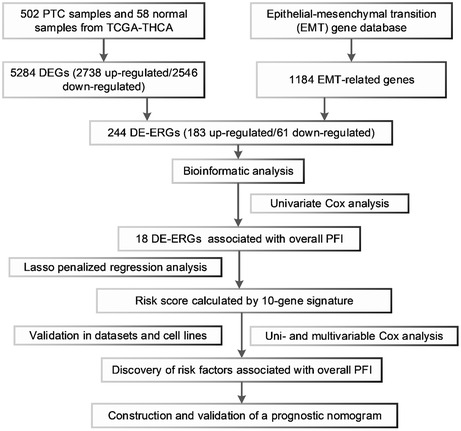

## 1 INTRODUCTION

With a 3% annual increase of the incidence of the past recent decades in the United States, thyroid cancer (TC) has become the most common endocrine malignant tumor and a leading public health issue.^1^ Although most cases (>90%) are papillary thyroid cancer (PTC) with a good prognosis, one of the main obstacles to the treatment is the continuing high rate of recurrence, distant metastasis, and even persistent disease.^2^, ^3^ Compared with cancer‐related morbidity of 13.5/100,000 in 2015, approximately 30% of PTC patients would suffer from postoperative recurrence, with the consequent requirement for reoperation.^4^ Postoperative recurrence not only results in a higher rate of surgical complications such as vocal cord paralysis or hypocalcemia but also with dedifferentiation events that coexist in metastatic lesions.^5^ In other words, anaplastic thyroid cancer (ATC), which accounts for 90% of TC‐related deaths, may occur in recurrent or metastatic sites.^6^ Hence, individualized therapy and surveillance based on precise prediction of the risk of recurrence remains a key focus of clinical research on PTC.^7^

Epithelial–mesenchymal transition (EMT) is a morphological alteration in which cells develop a fibroblast‐like phenotype following the initiation of mesenchymal cell‐related transcriptive events.^8^ In the process of EMT, epithelial cells dedifferentiate with a reduction in the expression of adhesion molecules (e.g., E‐cadherin), resulting in loss of the contact inhibition mediated by the tissue structure. Cells also undergo a change in morphology from a pebble to spindle shape caused by rearrangement of the cytoskeleton, loss of connection to the basement membrane, and acquisition of mesenchymal phenotypes.^9^ An increasing number of studies have demonstrated that EMT activation is closely related to multiple milestones in the development of cancer, including distant metastasis, extrathyroidal extension, and increased cancer cell stemness, and can be observed at the tumor‐stromal boundary of TC.^10^, ^11^ Due to the complicated process of EMT, which involves various transcriptional and translational factors associated with TC, ancillary diagnostic tools based on standardized genomic characteristics would offer better predictive value than risk stratification based on clinical parameters alone.^12^, ^13^ Based on the pivotal role of EMT in PTC invasion and anaplastic transformation, we established a novel 10‐gene signature derived from prognosis‐related differentially expressed EMT‐related genes (DE‐ERGs) using Cox proportional hazard analysis. Clinical validation on external datasets and experimental validation on PTC cell lines were also conducted. Thereafter, we used uni‐ and multivariate analyses to identify independent prognostic characters. We then constructed a stepwise regression model and a nomogram based on the risk score and other crucial clinical parameters to predict the prognosis of PTC patients. Subsequently, we verified the prognostic value of the novel ancillary model.

## 2 MATERIALS AND METHODS

### 2.1 Acquisition of TCGA‐THCA transcriptional data

We downloaded level‐3 RNA sequencing data in the form of fragments per kilobase of exon model per million reads mapped (FPKM) and counts from The Cancer Genome Atlas (TCGA) updated in July 2019.^14^ The TCGA, a landmark cancer genomics program, molecularly characterized over 20,000 primary cancer and matched normal samples spanning 33 cancer types. We applied a transcript per million (TPM) transformation and normalized the sequencing data with a base‐2 logarithm. Cases with a follow‐up period of less than a month were excluded. We obtained clinical data from the University of California，Santa Cruz (UCSC) Xena database, which provides interactive online visualization of seminal cancer genomics datasets, including data from TCGA and gene mutation status from the cBioPortal database.^15^

### 2.2 Differential classification of DE‐ERGs

We performed differential gene expression analysis of all the 502 PTC cases with 58 normal thyroid tissues from TCGA‐THCA dataset using the R package “EdgeR.” We identified DEGs according to the criteria of false discovery rate (FDR) <0.05 and |Log2FC| >1.^16^ A list of EMT‐related genes (ERGs) was obtained from the Epithelial‐Mesenchymal Transition Gene Database (dbEMT2, http://dbemt.bioinfo‐minzhao.org/).^17^ The DE‐ERGs were defined as the DEGs that intersected with the ERGs.

### 2.3 Functional annotation

The “clusterProfiler” package was used to annotate the enriched biological functions of the previously defined DE‐ERGs.^18^ Gene ontology (GO) and Kyoto Genes and Genomes (KEGG) pathway analyses were applied, and the terms biological process (BP), cellular component (CC), and molecular function (MF) were explored. The Benjamini–Hochberg method was used for false FDR correction.

### 2.4 Screening and assessment of the novel EMT signature

After differential gene expression analysis, all the 488 PTC cases from TCGA thyroid cancer (THCA) dataset were allocated randomly into the training and the testing datasets on a ratio of 0.8.^19^ The baseline characteristics of the 488 PTC patients in TCGA‐THCA dataset are presented in Table 1. We then used a univariate Cox regression model to screen the genes that were significantly related to progression‐free interval (PFI) (*P* < 0.05) in the training dataset from the DE‐ERGs. Further, crucial genes and relevant coefficients were selected from the PFI‐related DE‐ERGs by Least absolute shrinkage and selection operator (LASSO) regression analysis with the “glmnet” R package.^20^ To assess the efficacy of stratification of different risks of recurrence, we defined the PTC cases as “low risk” or “high risk” of recurrence according to the optimum cutoff defined by X‐Tile.^21^ Finally, we evaluated of the predictive power of the novel EMT signature by calculation of the area under the curve (AUC) of the receiver operating characteristic (ROC) curve in training, validation, and total set,^22^ and Kaplan–Meier (K–M) analysis and determination of Harrell's C‐index using the “survcomp” and “survival” packages of R.^23^ Next, we analyzed the correlation between the EMT signature and clinical characters including age, gender, BRAF mutation status, TNM stage, extrathyroidal extension, residual tumor and primary focality in TCGA‐THCA cohort. Since the prognosis of PTC/ poorly differentiated thyroid cancer (PDTC)/ATC varies from relatively well to very poor, we were interested in the role of EMT signature in TC samples with different degree of differentiation. To partially address this issue, we implemented three datasets which contains PTC, PDTC, and ATC samples from GSE29265, GSE33630,^24^ and GSE76039^25^ to calculate risk score with EMT signature and immune signature previously reported by Lin et al.^26^ In order to prove the predictive power of the signature, we compared with the predictive power of other clinical information including American Thyroid Association (ATA) stratification, American Joint Committee on Cancer (AJCC) stage, and (Metastasis, Age, Completeness of resection, Invasion, and Size) (MACIS) score. To validate the clinical value of the EMT signature, we also implemented GSE138042,^27^ GSE82208, GSE58545,^28^ and GSE60542^29^ including TC samples of different stage and tissue types. All the GSE datasets were obtained from Gene Expression Omnibus (GEO); the information is listed in Table 3.

**TABLE 1:** Baseline of enrolled 488 PTC patients in the TCGA‐THCA dataset

Characteristic	Training	Testing	*p*
*n*	380	108	
Progression, n (%)			0.901
Free	341 (69.9%)	98 (20.1%)	
Progression	39 (8%)	10 (2%)	
RAS_status, *n* (%)			0.602
Mutated	48 (9.8%)	11 (2.3%)	
Wild type	332 (68%)	97 (19.9%)	
BRAF_status, *n* (%)			0.482
Mutated	212 (43.4%)	65 (13.3%)	
Wild type	168 (34.4%)	43 (8.8%)	
Extrathyroid_extension, n (%)			0.756
Minimal (T3)	102 (21.7%)	28 (5.9%)	
Moderate/Advanced (T4)	13 (2.8%)	5 (1.1%)	
None	255 (54.1%)	68 (14.4%)	
Histological_type, *n* (%)			0.348
Classical/usual	268 (54.9%)	83 (17%)	
Follicular	84 (17.2%)	17 (3.5%)	
Tall Cell	28 (5.7%)	8 (1.6%)	
Neoplasm_focus_type, n (%)			0.101
Multifocal	178 (37.2%)	40 (8.4%)	
Unifocal	195 (40.8%)	65 (13.6%)	
Anatomic_site, *n* (%)			0.113
Bilateral	69 (14.3%)	12 (2.5%)	
Isthmus	19 (3.9%)	3 (0.6%)	
Unilateral	286 (59.3%)	93 (19.3%)	
Residual_tumor, *n* (%)			0.677
R0	291 (68.3%)	80 (18.8%)	
R1	38 (8.9%)	13 (3.1%)	
R2	3 (0.7%)	1 (0.2%)	
Ajcc_stage, *n* (%)			0.716
Stage I	215 (44.2%)	58 (11.9%)	
Stage II	37 (7.6%)	14 (2.9%)	
Stage III	84 (17.3%)	26 (5.3%)	
Stage IV	42 (8.6%)	10 (2.1%)	
M_stage, *n* (%)			0.691
M0	372 (76.4%)	107 (22%)	
M1	7 (1.4%)	1 (0.2%)	
N_stage, *n* (%)			0.378
N0	180 (41.1%)	45 (10.3%)	
N1	162 (37%)	51 (11.6%)	
T_stage, *n* (%)			0.960
T1	111 (22.8%)	30 (6.2%)	
T2	126 (25.9%)	35 (7.2%)	
T3	125 (25.7%)	38 (7.8%)	
T4	16 (3.3%)	5 (1%)	
Gender, *n* (%)			0.097
Female	286 (58.6%)	72 (14.8%)	
Male	94 (19.3%)	36 (7.4%)	
Age, *n* (%)			1.000
<55	253 (51.8%)	72 (14.8%)	
≥55	127 (26%)	36 (7.4%)	
Progression_free_interval, median (IQR)	888.5 (496.5, 1463.75)	927 (460, 1340.75)	0.671

### 2.5 Gene set enrichment analysis (GSEA) of the ERG‐based classifier

We explored the potential turbulence in the ERG‐based signature at the transcriptional level by GSEA.^30^ All the 488 PTC cases from the THCA set were defined as low‐ or high‐risk according to the optimum cutoff defined by X‐Tile. We then explored the upregulated pathways in high‐risk group using GSEA V4.1 software. C5: GO and C6: oncogenic signatures were used as background.

### 2.6 Cell culture and lysis

Normal human thyroid follicular cell line Nthy‐ori 3.1,^31^ and PTC cell lines B‐CPAP (primary and well differentiated)^32^ and KTC‐1 (metastatic and refractory to radio iodine)^33^ were kindly provided by the National Collection of Authenticated Cell Cultures of the Chinese Academy of Sciences. B‐CPAP and KTC‐1 were cultured under the circumstance of 5% CO_2_, 37°C, in RPMI Medium 1640 (Invitrogen) with 10% FBS (Gibco), non‐essential amino acids (Invitrogen), Glutamax (Invitrogen), and Sodium Pyruvate (Invitrogen, 11,360,070) added. TRIzol (Lablead) was used to lysate and isolate RNA from cells according to the manufacturer's protocol.

### 2.7 Quantitative real‐time Polymerase Chain Reaction (RT‐qPCR)

RT‐qPCR was conducted after isolation of RNA and performed essentially as described previously with housekeeper (GAPDH) mRNA for normalization via the 2^−ΔΔCt^ method.^34^ The sequences of primers are listed in Table 4.

### 2.8 Identification of prognosis‐associated factors

Univariate and multivariate Cox analyses were implemented to extract the correlating prognostic parameters. EMT risk score and clinical information included age, gender, BRAF mutation status, RAS mutation status, Neoplasm_size, TNM stage, extrathyroidal extension, residual tumor, primary focality, and anatomic site. Categorical variables including gender, age (over 55 or not), BRAFV600E, RAS, TNM, extrathyroidal extension, residual tumor, primary focality, anatomic site, and histological type were transformed into rank variables. We firstly conducted univariate analysis and excluded factors with *p* > 0.1 for further multivariate analysis.

### 2.9 Implementation and validation of the novel predictive model

We constructed a stepwise Cox model after collinearity tests for predicting the PTC's PFI. Clinical factors in multivariate analysis and other previously reported significantly correlated ones were included. Thereafter, the model was visualized as a nomogram. The cutoff value to define high‐ or low‐risk groups was generated by X‐Tile based on the nomogram points for each case. We then assessed the predictive ability of the model based on the AUC of the ROC curve, the C‐index, and the calibration curve, which was generated with 1000 times bootstrap resampling.

### 2.10 Statistical analysis

Statistical analyses were conducted using R version 4.0.3 (Bunny‐Wunnies Freak Out) and GraphPad Prism 8.4.3 (GraphPad Software, CA, USA). Codes used for analyses are listed in supplementary document S1. Continuous data were analyzed using unpaired *t*‐tests. Categorical variables were analyzed using chi‐squared tests. K–M survival curves were analyzed with the Gehan–Breslow–Wilcoxon test. Multiple testing correction were applied with Benjamini and Hochberg method for difference and enrichment analyses. *p*‐values <0.05 were considered to indicate statistical significance.

## 3 RESULTS

### 3.1 Determination of DE‐ERGs

**FIGURE 1:**
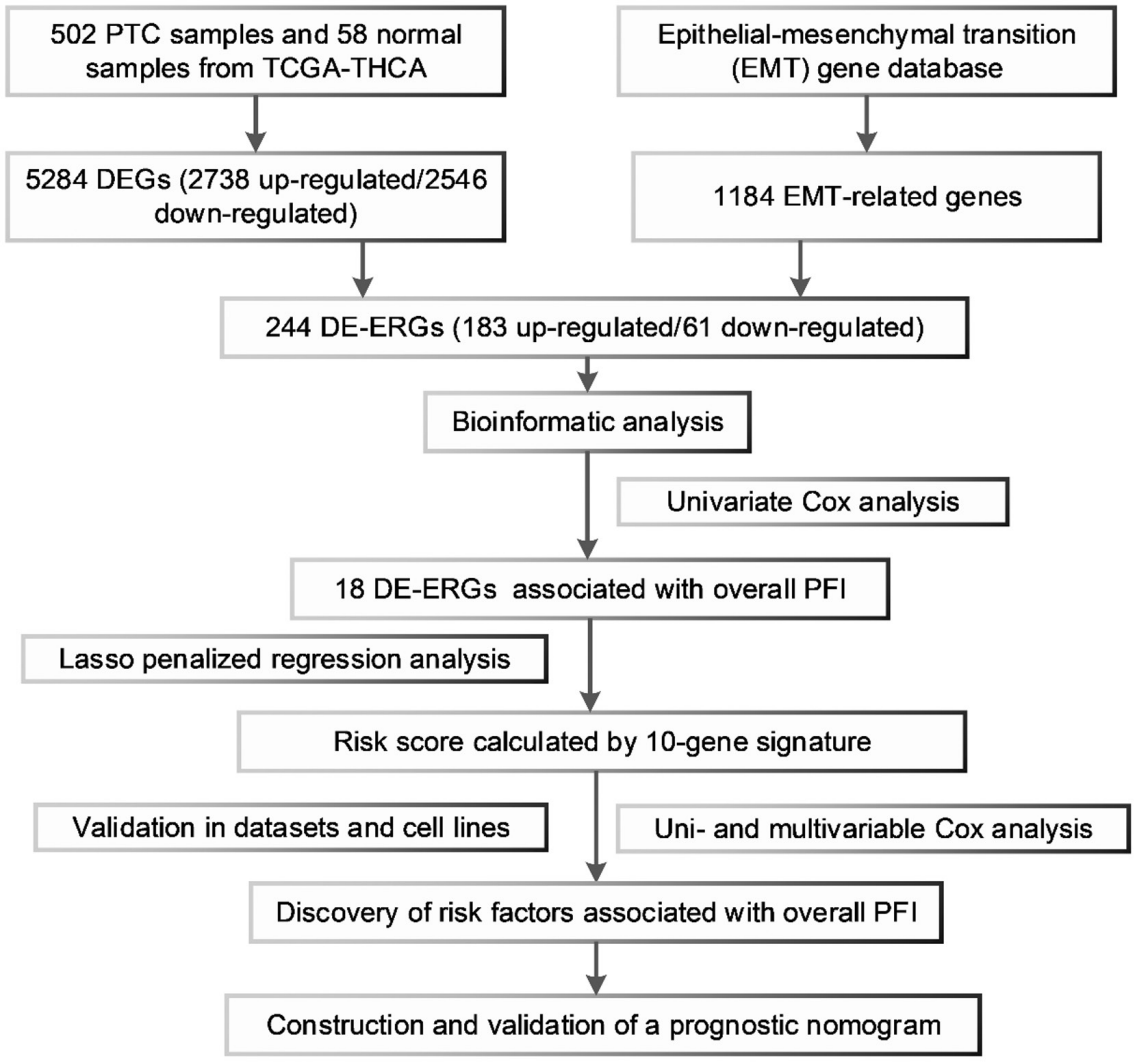
Flowchart showing the workflow of our analysis

**FIGURE 2:**
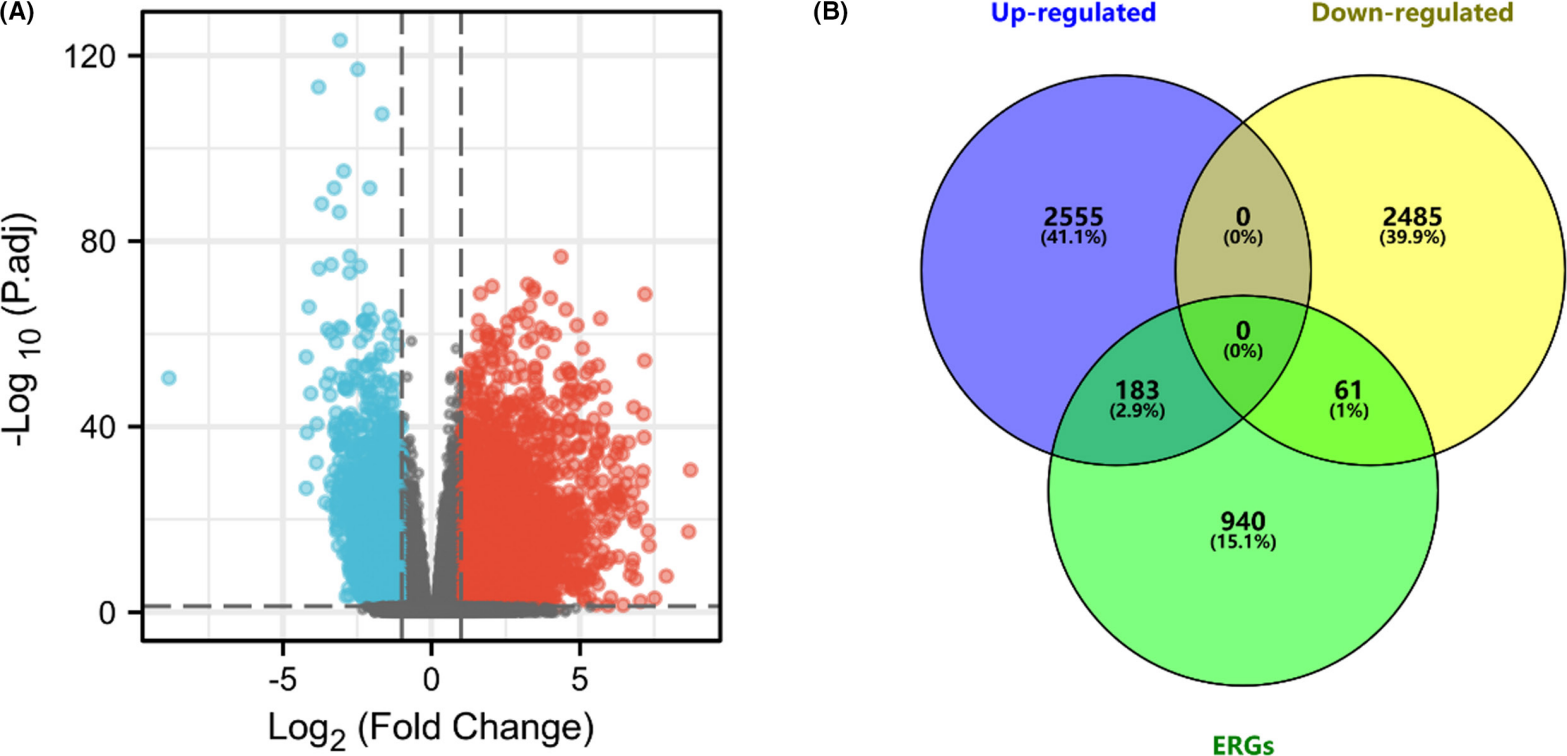
Differentially expressed gene (DEG) analysis and intersection showing the differentially expressed EMT‐related genes (ERGs) (DE‐ERGs). (A) Volcano plot showing the DEG analysis between the 502 PTC samples and 58 normal thyroid samples. (B) Venn diagram showing the intersection of 5284 DEGs with the list containing 1184 DE‐ERGs derived from dbEMT 2.0.

The workflow of our study is shown in Figure 1. In total, we enrolled 488 PTC cases with complete follow‐up information for our analysis. Through differential gene expression analysis of 502 PTC samples and 58 normal thyroid tissues from TCGA‐THCA, we retrieved a total of 5284 DEGs (2738 up‐ and 2546 downregulated) (Figure 2A). Furthermore, we downloaded the list of 1184 ERGs from dbEMT2. The intersection contained 244 DE‐ERGs (183 up‐ and 61 downregulated) (Figure 2B). The full list of DEGs, ERGs, and 244 DE‐ERGs is shown in Tables S1–S3.

### 3.2 Functional annotation of the 244 DE‐ERGs

**FIGURE 3:**
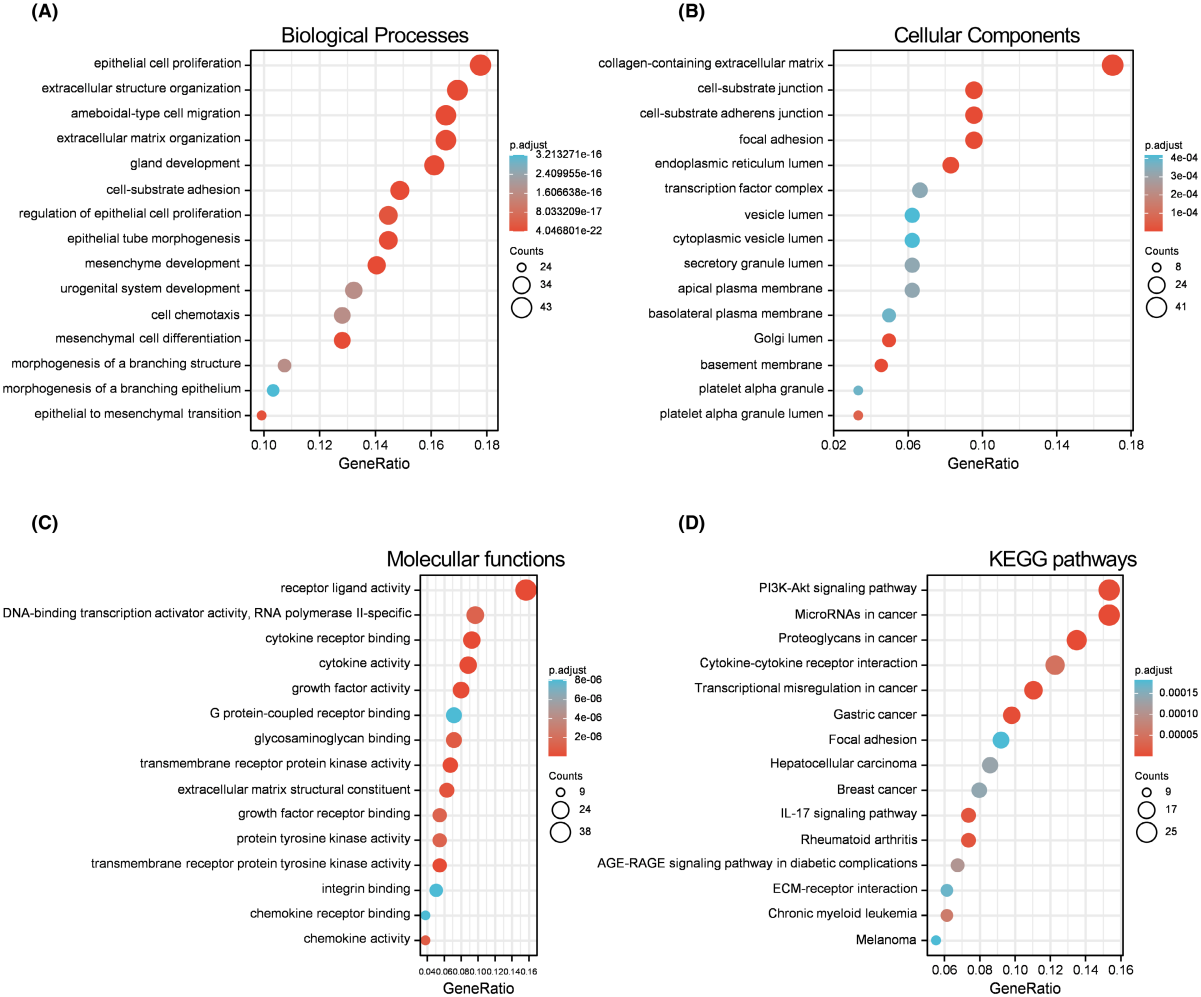
Functional annotations of the 244 DE‐ERGs. Bar charts showing the annotation of 244 DE‐ERGs by gene ontology (GO) analysis and KEGG pathway analysis. (A–C) The predominantly enriched terms of cellular component, biological process, and molecular function. (D). The predominantly enriched pathways of the 244 DE‐ERGs.

Annotation of the 244 DE‐ERGs by GO and KEGG pathway analyses is shown in Figure 3. For the biological process (BP) category, the DE‐ERGs were mainly enriched in epithelial cell proliferation, extracellular structure organization, and regulation of epithelial cell proliferation (Figure 3A). In the terms of the cellular component (CC) category, the DE‐ERGs were mainly enriched in collagen‐containing extracellular matrix, cell‐substrate junction, and focal adhesion (Figure 3B). In the terms of the molecular function (MF) category, the DE‐ERGs were mainly enriched in receptor ligand activity, DNA‐binding transcription activator activity, and polymerase II‐specific (Figure 3C). In terms of KEGG pathways, the DE‐ERGs were mainly enriched in PI3K‐Akt signaling pathway, microRNAs in cancer, and proteoglycans in cancer (Figure 3D).

### 3.3 Screening of significant DE‐ERGs and construction of the 10‐gene signature

**FIGURE 4:**
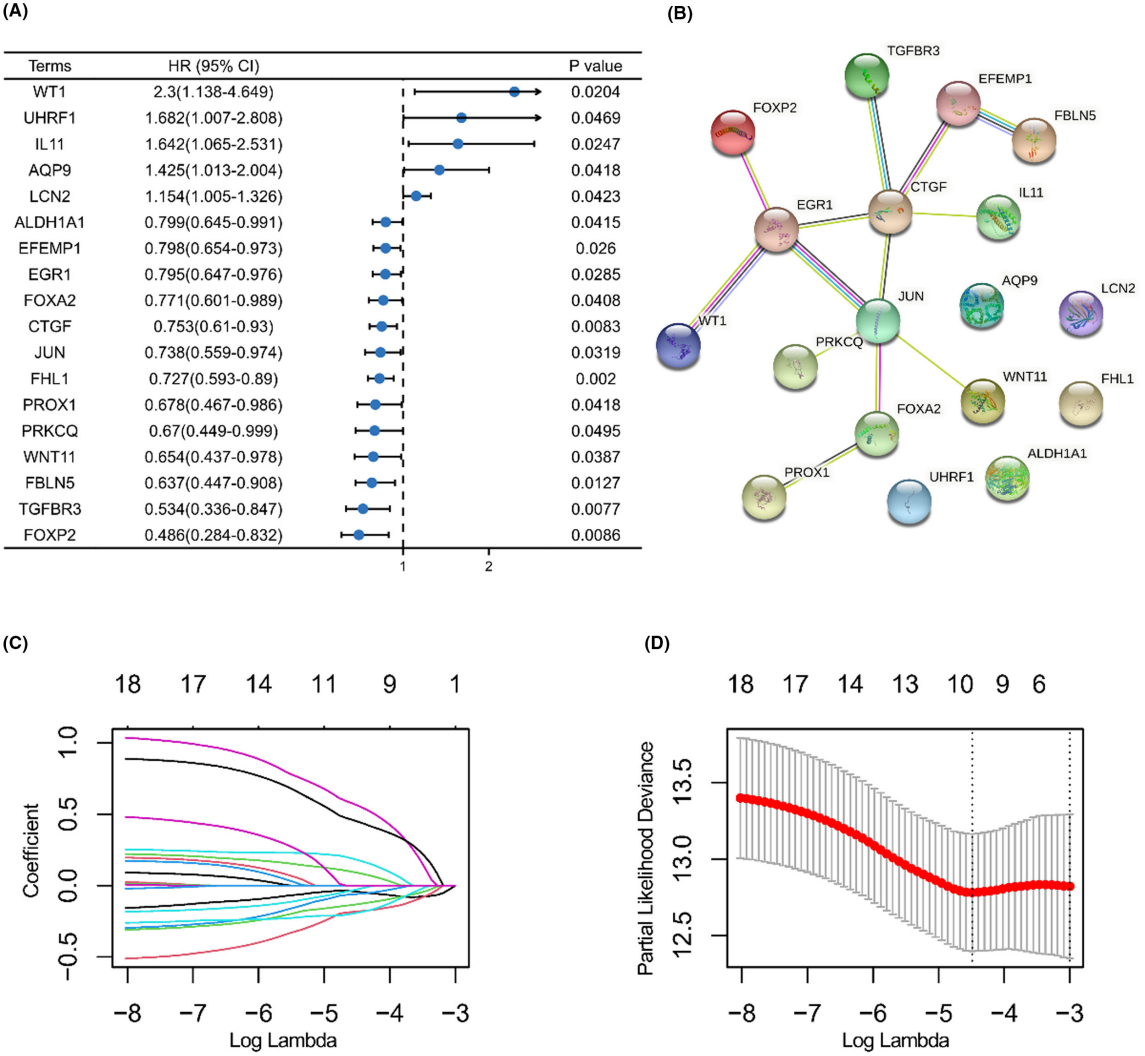
Screening of prognosis‐related genes and identification of the 10‐gene signature. (A) Forest plot showing the 18 prognosis‐related genes filtered by Cox proportional‐hazards analysis with criterion of a *p* < 0.05. (B) Potential PPI network of the 18 DE‐ERGs. (C) LASSO coefficient profiles of the 18 DE‐ERGs. (D) Lasso deviance profiles of the 18 DE‐ERGs. The lambda selection criterion was based on the value of lambda giving a minimum mean cross‐validation error; lambda min = 0.01124.

The baseline information of the training and testing sets allocated from the 488 PTC cases is shown in Table 1. A total of 18 PFI‐related DE‐ERGs were identified; full results are shown in Table S4. Forest plots of the logfc, *p‐*value, and hazard ratio of each item are shown in Figure 4A. The potential protein–protein interaction network of the 18 items was explored using the STRING database.^35^ In the complex network, five proteins (EFEMP1, CTGF, EGR1, JUN, and FOXA2) were identified as key nodes (Figure 4B). LASSO penalty regression analysis reduced and constructed a novel 10‐gene signature (Figure 4C,D). The risk score was calculated as follows: risk score = exp/FHL1 × (−0.04395) + exp/CTGF × (−0.13087) + exp/FOXP2 × (−0.05892) + exp/FBLN5 × (−0.19037) + exp/WT1 × 0.560368 + exp/IL11 × 0.448262 + exp/AQP9 × 0.088904 + exp/TGFBR3 × (−0.18178) + exp/WNT11 × (−0.02575) + exp/UHRF1 × 0.178616. The coefficient of variation (CV) in training and testing sets was 26.57% and 26.95%.

### 3.4 Verification of the discriminatory power of the 10‐gene signature

**FIGURE 5:**
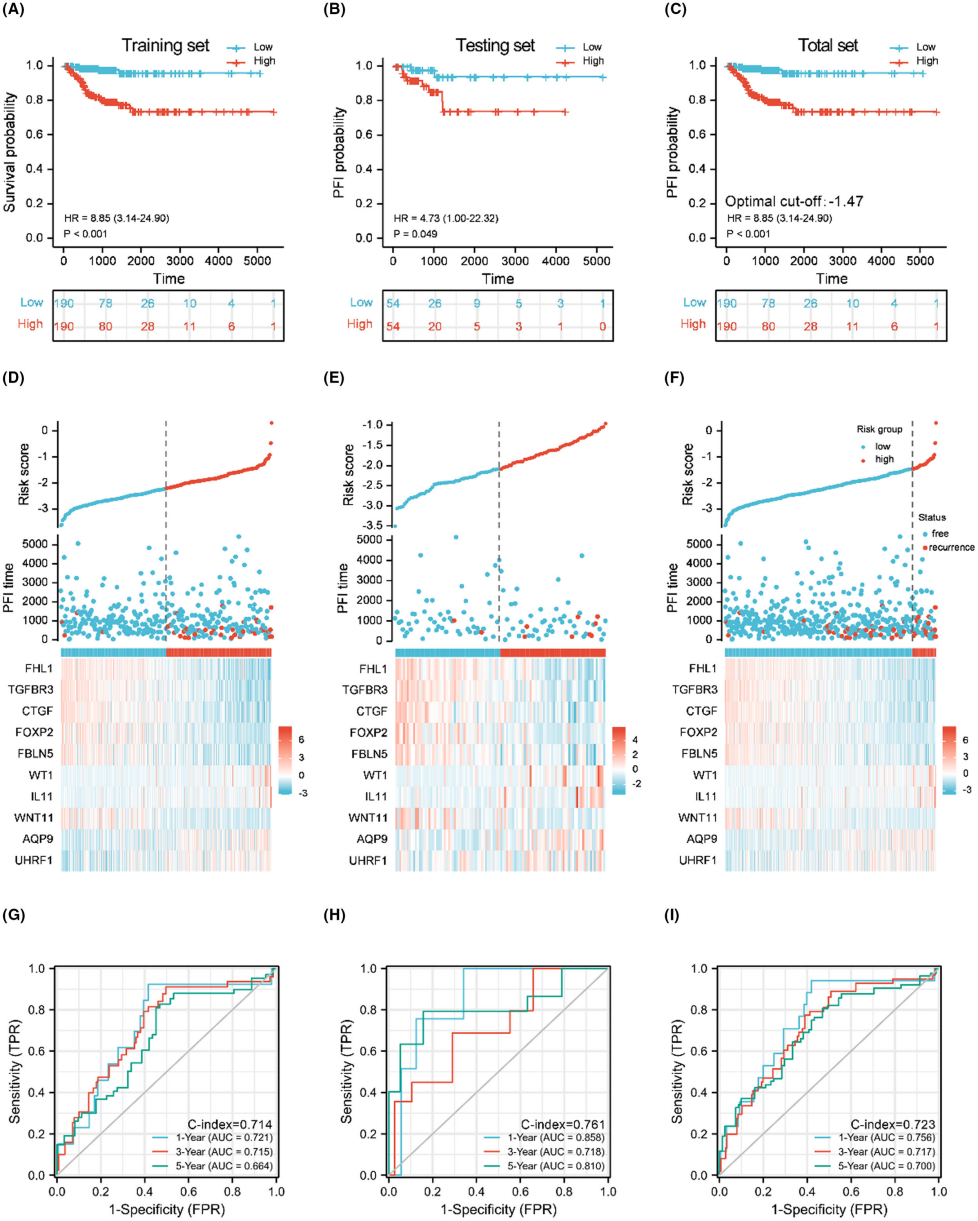
Verification of the discriminative power and predictive efficacy of the 10‐gene signature. 80% of TCGA‐THCA dataset cases (*n* = 380) were allocated into the training set (A, D, G) and the left 20% cases (*n* = 188) were allocated into the validation set (B, E, H); results for the total set are also presented (C, F, I). Cases were defined as “high risk” or “low risk” according to the median value (training and testing set) or optimal risk score cutoff determined by X‐Tile (total set), and the PFI correlation was evaluated by K–M analysis. (A–C) The proportion of cases of PFI in the different risk category groups at different timepoints. (D–F) Scatterplots showing the distribution of recurrence (red dots) or progression‐free events (blue dots) in the different risk groups. (G–I) The area under the curve (AUC) and C‐index of the receiver operating characteristic (ROC) curve showing the predictive efficacy of 10‐gene signature in 1‐, 3‐, and 5‐year follow‐up periods.

According to K–M analysis, the 10‐gene signature provided good discriminatory power for patients with high or low risk of recurrence (Figure 5A–C). PTC cases with higher risk score due to the optimum cutoff value of higher risk had a significantly poorer prognosis (*p* < 0.0001, Figure 5C).The relationships between risk score and recurrence events are shown in scatterplots (Figure 5D–F). In the training set, the AUCs for 1‐year, 3‐year, and 5‐year PFI prediction based on the 10‐gene signature were 0.721, 0.715, and 0.664, respectively, with a C‐index of 0.714 (Figure 5G). In the testing set, the AUCs were 0.858, 0.718, and 0.810, respectively, with a C‐index of 0.761 (Figure 5H). In the THCA total dataset, the AUCs were 0.756, 0.717, and 0.700, respectively, with a C‐index of 0.723 (Figure 5I). The optimal cutoff value for discriminating high risk patients of PTC on Illumina Hiseq 2500 platform was −1.47. Surprisingly, we observed statistically significant differences of undifferentiated TC to differentiated TC in all the three datasets (Figure S1A–C). However, for the risk score using the classifier reported by Lin et al, the difference was statistically significant only in GSE33630. We supposed that the optimal cutoff value for discriminating ATC from PTC on Affymetrix Human Genome U133 Plus 2.0 Array platform was 1.78. (Figure S1D–F). Furthermore, in GSE138042, we verified the classifier in samples with different disease stage (T1/T2 versus T3/T4) and found a statistically significant difference (*p* < 0.05). In GSE82208, the difference of risk score between follicular adenoma and follicular thyroid cancer (FTC) was statistically significant (*p* < 0.0001). Also, in GSE60542, GSE58545, GSE29265, and GSE33630, the difference of risk score between normal and PTC samples was statistically significant (*p* < 0.0001). And for discriminating adenoma from FTC, the optimal cutoff value would be −0.95. (Figure S2A–F). Full results are also listed in Tables S5 and S6.

### 3.5 GSEA

**FIGURE 6:**
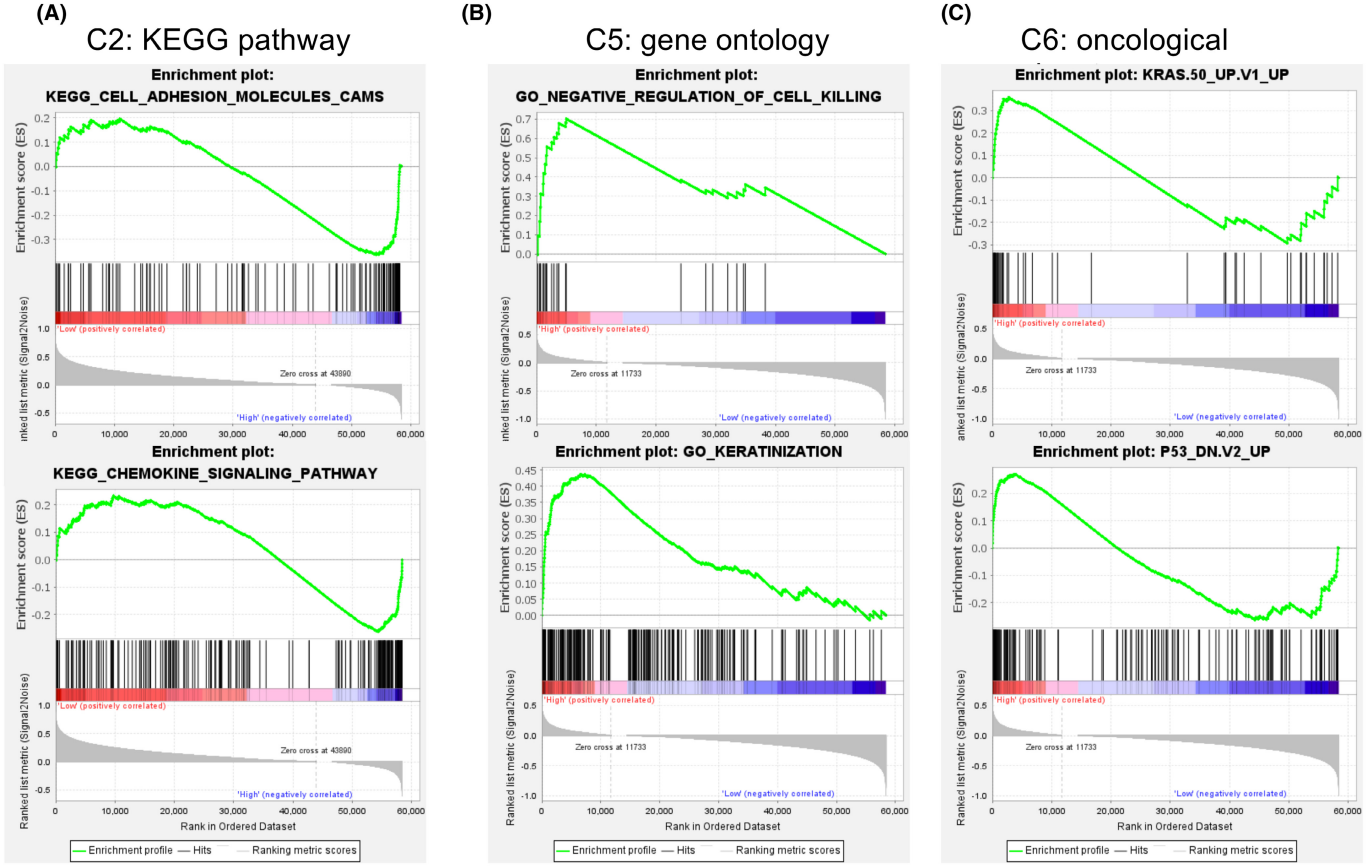
Gene set enrichment analysis of the turbulence at the molecular level caused by the signature. (A) The gene sets upregulated in the “high‐risk” phenotype group of C2: KEGG pathway. (B) The gene sets upregulated in the “high‐risk” phenotype group of C5: gene ontology. (C) The gene sets upregulated in the “high‐risk” phenotype of C6: oncological signatures.

GSEA in the 488 PTC cases from THCA dataset showed the representative altered biological functions of the high‐risk group (Figure 6A–C). In the terms of KEGG pathway, the transcriptional alterations were annotated to the processes such as cell adhesion molecules and chemokine signaling pathway. In the terms of gene ontology, the transcriptional alterations were annotated to the processes such as negative regulation of cell killing and keratinization. For the oncological signatures, multiple classical oncological pathways, including the p53, the KRAS pathways were altered in the high‐risk group. Full results are shown in Table S7.

### 3.6 RT‐qPCR quantification and EMT signatures in PTC cell lines

**FIGURE 7:**
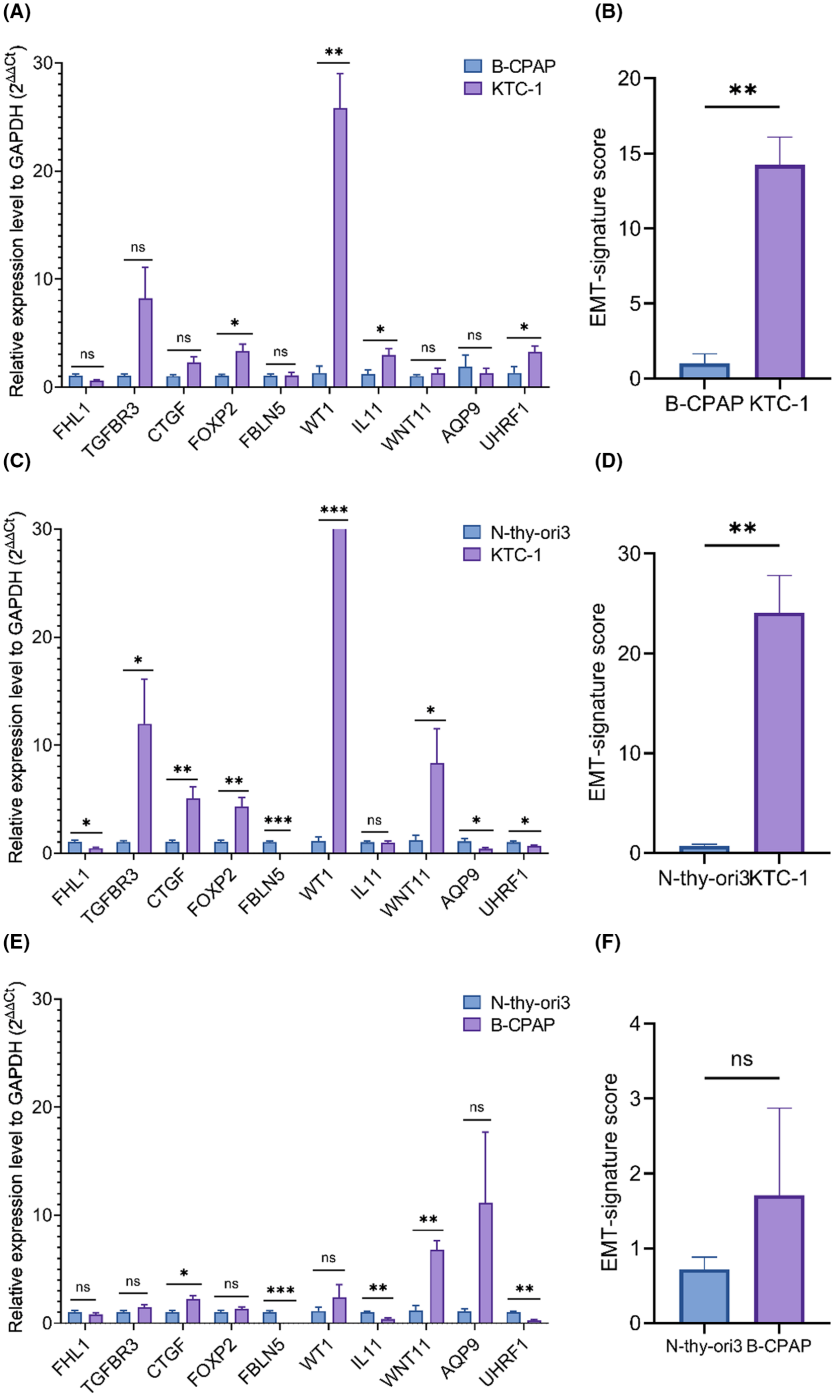
RT‐qPCR quantification and EMT signatures in PTC cell lines. (A, C, E) Relative expression level of 10 genes to GAPDH (2^−ΔΔCT^) in Nthy‐ori 3.1, B‐CPAP and KTC‐1 cell line. (B, D, F) Risk score of PTC cell lines calculated by EMT signature. Data are presented as interleaved bar plot. Unpaired *t*‐test with Welch's correction (*n* = 4), *p < 0.05, **p < 0.01.

The relative 10‐gene expression level of EMT signature in B‐CPAP and KTC‐1 cell were generated through RT‐qPCR quantification. In 10 genes of EMT signature, the expression level of FOXP2, WT1, IL11, and UHRF1 were higher in KTC‐1 than B‐CPAP, the differences were statistically significant (*p* < 0.05). The differences of FHL1, TGFBR3, CTGF, FBLN5, WNT11, and AQP9 were not statistically significant (*p* > 0.05), as shown in Figure 7A. The EMT risk score of KTC‐1 was higher than B‐CPAP, as shown in Figure 7B. The expression level of TGFBR3, CTGF, FOXP2, WT1, and WNT11 was higher in KTC‐1 than normal thyroid cell line Nthy‐ori 3.1, and the differences were statistically significant (*p* < 0.05), as shown in Figure 7C. The risk score of KTC‐1 was also higher than Nthy‐ori 3.1, and the difference was statistically significant, as shown in Figure 7D. The expression level of CTGF and WNT11 was higher in B‐CPAP than Nthy‐ori 3.1, and the differences were statistically significant (*p* < 0.05), as shown in Figure 7E. The risk score of B‐CPAP was higher than Nthy‐ori 3.1, but the difference was not statistically significant, as shown in Figure 7D.

### 3.7 Clinical correlation of the EMT signature

**FIGURE 8:**
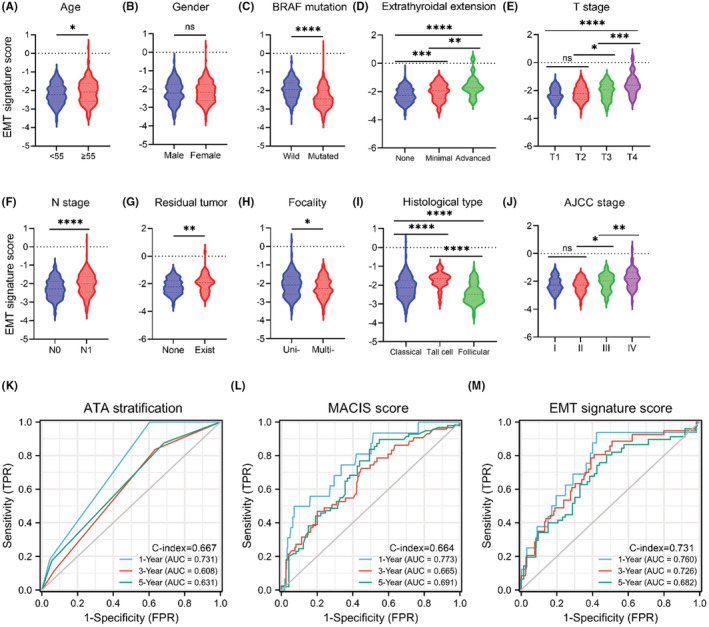
Clinical correlation of the EMT signature in TCGA‐THCA cohort. (A‐E) The distribution of the risk score according to different status of age, gender, BRAF mutation, extrathyroidal invasion, and T stage in the TCGA‐THCA dataset. (F‐J) The distribution of the risk score according to different status of N stage, residual tumor, focality type, histological type, and AJCC stage in the TCGA‐THCA dataset. (K‐M) The AUC and C‐index of the ROC curve showing the predictive efficacy of ATA stratification, MACIS score and 10‐gene signature in 1‐, 3‐, and 5‐year follow‐up periods. Data are presented as violin plot. Unpaired *t*‐test with Welch's correction, *p < 0.05, ****p < 0.0001.

Next, we analyzed the correlation between the EMT signature and clinical characters. In groups divided by age, patients who were over 55 had higher risk score than patients who were younger than 55 (Figure 8A). In groups divided by BRAF status, wild‐type samples had higher risk score than who were mutated (Figure 8C). In terms of extrathyroidal invasion, patients with no extra‐invasion had lower risk score than those with extra‐invasion existence (Figure 8D). Patients in T1/T2 had lower risk score than those in an advanced disease stage (T3/T4) (Figure 8E). Patients in N1 had higher risk score than those without lymph node metastases (N0) (Figure 8F). In groups divided by residual tumor, patients without residual tumor had lower risk score than patients with residual tumor (Figure 8G). In groups divided by focality type, unifocal samples had higher risk score than multifocal samples (Figure 8H). Patients with aggressive histological (tall cell) type have higher risk score than those with nonaggressive histological type (Figure 8I). Patients in stage 1/2 had lower risk score than those in stage 3/4 (Figure 8J). The differences were statistically significant (*p* < 0.05). For the terms divided by gender, the difference was not statistically significant (*p* > 0.05), as shown in Figure 8B; full clinical characters are as shown in Table S8. We also compared AUCs of ATA stratification, MACIS score,^36^ and risk score in 435 patients from TCGA‐THCA with complete information. For ATA stratification, the AUCs for 1‐year, 3‐year, and 5‐year PFI prediction were 0.731, 0.608, and 0.631, respectively, with a C‐index of 0.667 (Figure 8K). For MACIS score, the AUCs for 1‐year, 3‐year, and 5‐year PFI prediction were 0.773, 0.665, and 0.691, respectively, with a C‐index of 0.664 (Figure 8L). For EMT signature risk score, the AUCs for 1‐year, 3‐year, and 5‐year PFI prediction were 0.760, 0.726, and 0.682, respectively, with a C‐index of 0.731 (Figure 8M), as shown in Table S9.

### 3.8 Identification of independent prognosis‐related parameters

The univariate Cox analysis revealed that parameters including age (≥55), neoplasm_size (≥2 cm), histological_type (tall cell), T stage (T3/T4), M stage (M1), extrathyroidal extension, and risk score were significantly associated with prognosis (*p* < 0.05, Table 2). After exclusion of samples with incomplete information and *p* < 0.1 in univariate analysis, a total of 422 patients were enrolled in multivariate analysis. In multivariate analysis, neoplasm_size (≥2 cm) and risk score were significantly associated with prognosis (*p* < 0.05, Table 2). Neoplasm_size (≥2 cm) and risk score were identified as independent prognosis‐related factors in both the uni‐ and multivariate analysis.

**TABLE 2:** Univariate and multivariate analyses of prognosis‐related characters

Characteristics	Total (*N*)	Univariate analysis		Multivariate analysis	
		Hazard ratio (95% CI)	*P*‐value	Hazard ratio (95% CI)	*p‐*value
Gender	488				
Male	130	Reference			
Female	358	0.572 (0.320–1.024)	0.060	0.749 (0.391–1.435)	0.384
Age	488				
<55	325	Reference			
≥55	163	2.259 (1.289–3.961)	**0.004**	1.468 (0.659–3.268)	0.347
Risk score	488	4.335 (2.641–7.116)	**<0.001**	2.685 (1.462–4.933)	**0.001**
BRAF_status	488				
Wild type	211	Reference			
Mutated	277	1.455 (0.801–2.645)	0.218		
RAS_status	488				
Wild type	429	Reference			
Mutated	59	1.640 (0.768–3.504)	0.201		
Extrathyroid_extension	471				
None/Minimal	453	Reference			
Moderate/Advanced	18	2.100 (0.754–5.847)	0.156		
Neoplasm_size	474				
<2 cm	153	Reference			
≥2 cm	321	3.914 (1.547–9.901)	**0.004**	2.675 (1.022–7.001)	**0.045**
Histological_type	488				
Classical/Follicular	452	Reference			
Tall Cell	36	2.417 (1.084–5.389)	**0.031**	1.293 (0.500–3.345)	0.596
Anatomic_site	482				
Unilateral	379	Reference			
Bilateral	81	1.101 (0.513–2.365)	0.805		
Isthmus	22	0.413 (0.057–3.004)	0.382		
M_stage	487				
M0	479	Reference			
M1	8	5.630 (2.021–15.687)	**<0.001**	1.618 (0.434–6.031)	0.473
Ajcc_stage	486				
Stage I/II	324	Reference			
Stage III/IV	162	2.753 (1.567–4.839)	**<0.001**	1.386 (0.543–3.532)	0.495
N_stage	438				
N0	225	Reference			
N1	213	1.736 (0.950–3.172)	0.073	1.055 (0.526–2.114)	0.881
T_stage	486				
T1/T2	302	Reference			
T3/T4	184	2.806 (1.569–5.018)	**<0.001**	1.221 (0.577–2.581)	0.602

Bold values in Table 2 indicated the statistically significant (*p* < 0.05).

**TABLE 3:** External GEO datasets for validation of EMT signature

Datasets	References	Platform	Samples	Usage
GSE82208	Contributed by Wojtas B, et, al.	Affymetrix Human Genome U133 Plus 2.0 Array	25 Adenomas, 27 FTCs	External Validation
GSE29265	Contributed by Tomas G, et, al.	Affymetrix Human Genome U133 Plus 2.0 Array	20 PTCs, 20 Normals, 9 ATCs	External Validation
GSE33630	^24^	Affymetrix Human Genome U133 Plus 2.0 Array	49 PTCs, 45 Normals, 11 ATCs	External Validation
GSE60542	^29^	Affymetrix Human Genome U133 Plus 2.0 Array	33 PTCs, 30 Normals	External Validation
GSE76039	^25^	Affymetrix Human Genome U133 Plus 2.0 Array	20 ATCs, 17 PDTCs	External Validation
GSE58545	^28^	Affymetrix Human Genome U133A Array	27 PTCs, 18 Normals	External Validation
GSE138042	^27^	Illumina HiSeq 3000 (Homo sapiens)	50 PTCs (T1/T2), 24 PTCs (T3/T4)	External Validation

**TABLE 4:** Sequences of primers for RT‐qPCR analyses

Target gene	Primer	Sequence (5′‐3′)
GAPDH	Forward	AGTCCCTGCCACACTCAG
	Reverse	TACTTTATTGATGGTACATGACAAGG
FHL1	Forward	GACTGCTTCACCTGTAGTAACT
	Reverse	AGGTAACACACACAAAGCAATC
CTGF	Forward	ATTCTGTGGAGTATGTACCGAC
	Reverse	GTCTCCGTACATCTTCCTGTAG
FOXP2	Forward	AGCTCTGAAGTAAGCACAGTAG
	Reverse	TGCTGCTGTAAAAGAAGTTGTC
FBLN5	Forward	CTGTGACCCAGGATATGAACTT
	Reverse	TTGTAAATTGTAGCACGTCTGC
WT1	Forward	CCAATACAGAATACACACGCAC
	Reverse	CATCTGTAAGTGGGACAGCTTA
IL11	Forward	GTGGCCAGATACAGCTGTC
	Reverse	GAATTTGTCCCTCAGCTGTG
WNT11	Forward	TCTGCATGAAGAATGAGAAGGT
	Reverse	CACCAGTGGTACTTACAGTGG
AQP9	Forward	ATACCCAGCTCCGTATCTATCT
	Reverse	GTCAAAGATGGCAAAGACGATT
UHRF1	Forward	AATGTCAAGGGTGGCAAGAATA
	Reverse	GCCAGTATTTCACAACCTTGTA
TGFBR3	Forward	TCCTCTGAATGGCTGCGGTACTC
	Reverse	GGCTGGAACCTGTATCACAATGGAG

### 3.9 Establishment and validation of the novel signature‐based nomogram

**FIGURE 9:**
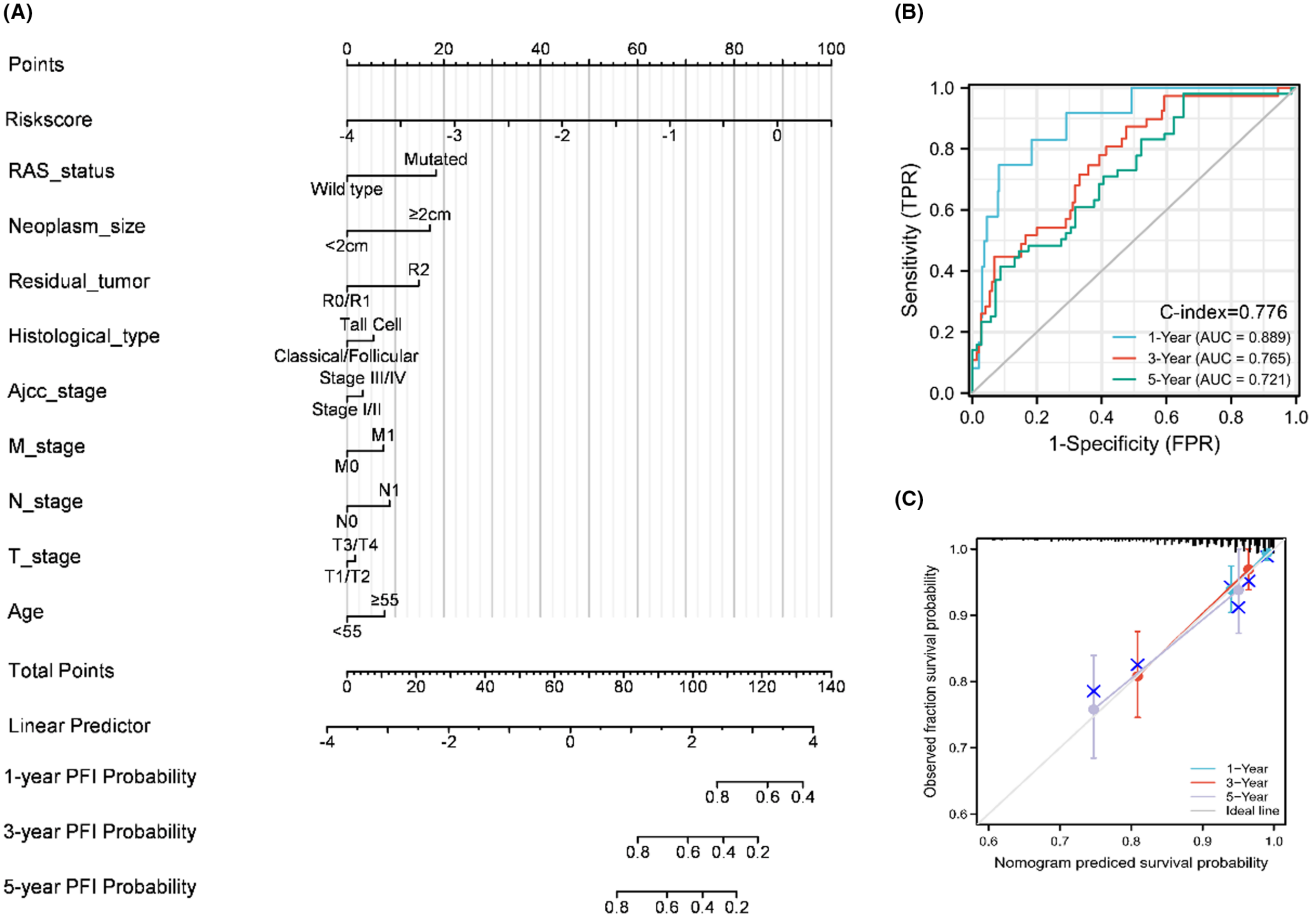
Establishment and assessment of the EMT‐based nomogram. (A) Visualization of the adjusted Cox proportional regression model of the risk score and relevant clinical parameters. (B) 1‐, 3‐, and 5‐year AUC and C‐index of the nomogram. (C) Calibration curve of the nomogram for the predicted and actual 1‐, 3‐, and 5‐year PFI.

We constructed a stepwise Cox regression model including the parameters of risk score, age, TNM stage, neoplasm size, residual tumor, histological type, and RAS status. The model was visualized in a predictive nomogram as shown in Figure 9A. The AUCs for 1‐year, 3‐year, and 5‐year PFI prediction were 0.889, 0.765, and 0.721, respectively, with a C‐index of 0.776 (Figure 9B). The evaluation of the predictive nomogram using a calibration curve revealed the efficacy and robustness of the model for prediction of the prognosis of PTC patients (Figure 9C). For the purposes of better understanding and use of this predictive nomogram, we uploaded a program that facilitates visualization of the prediction results using the “DynNom” package of R (https://liuruisurgeon.shinyapps.io/EMTbased_nomogram_PTC/).^37^ The progression‐free predictions are obtained by clicking the “predict” button after determination of the parameters (Figure S3).

## 4 DISCUSSION

As previously described, the progression of PTC, lymph node metastasis, stemness, and anaplastic transformation are closely related to EMT^38^; therefore, we hypothesized that EMT‐based signature would perform well in risk prediction and stratification of PTC patients. In our study, we extracted the EMT‐related genes from the dbEMT2, which curated over 1000 genes reported in more than 2000 published studies.^17^ Using integrated bioinformatics analysis, we established a novel EMT‐based signature for PFI prediction and recurrent risk stratification. Nomograms, which allow visualization of regression models, are widely used to provide an intuitive understanding of specific events based on enrolling meaningful characteristics.^39^ Traditional approach to staging, including the ATA recurrence stratification system, are widely used and practicable, but cannot be used for individualized and prospective risk evaluation prior to radical resection. The novel EMT‐related classifier and the relevant nomogram established in this study is economically feasible and simple to apply. Based on droplet digital PCR (ddPCR) technique with fine‐needle aspiration biopsy samples, the risk score could be obtained before surgery, making this a convenient approach to for decision‐making in relation to future therapy.^40^ For example, a more extensive neck dissection and close follow‐up after surgery would be taken if the PTC patient scored as high risk but with a relatively low risk in imaging or pathology examination. Another scenario is that indetermined FNA cases for PTC but with a high risk on the classifier, decision of active surveillance would be deliberated over. Since one of the optimum options for newly diagnosed low‐risk PTC is active surveillance, the risk score would provide accessible information that helps the patient to understand the decision to adopt this approach.^41^, ^42^ Moreover, we analyzed the correlations between EMT signature with relevant clinical characters, and found significant differences in terms divided by age, gender, BRAF mutation, residual tumor, focality, extrathyroidal invasion, histological type, and TNM stage. To the best of our knowledge, PTC patients with elder age, female gender, residual tumor, tall cell type, and advanced disease stage had significantly higher rates of all‐cause mortality and recurrence,^43^, ^44^, ^45^, ^46^ the results enhanced the potential clinical significance of EMT signature. Another interesting finding is the value of EMT signature in discovering possible dedifferentiation of thyroid cancer, which would partially address the mechanism of the ability of EMT signature in progression prediction, since anaplastic transformation are closely related to EMT.^38^ Further, the differences of risk score were statistically significant between follicular adenoma samples from follicular cancer, revealing a significant clinical value in FNA diagnosis, which remains challenging in differentiating FTC with benign follicular thyroid lesions.^47^ To validate the signature in clinical practice, we implemented multiple GEO datasets and confirmed the robustness of the signature in discriminating normal thyroid sample from PTCs. Also, we applied qPCR experiment of 10 genes on two PTC cell lines. According to background literatures, the B‐CPAP was originated from primary low‐risk PTC and with a relatively low invasiveness.^48^ The KTC‐1, which originated from advanced metastatic PTC and refractory to radio iodine therapy, was with lower degree of differentiation and invasiveness.^33^ Several significantly highly expressed genes and the higher risk score in KTC‐1 than in B‐CPAP also confirmed the connection between EMT signature with PTC invasion and dedifferentiation. The results also confirmed the intrinsic connection between EMT signature with dedifferentiation and invasiveness. Furthermore, the risk score was also lower in immortalized thyroid follicular cell line than in B‐CPAP. However, the difference was not statistically significant, indicating the mild invasiveness and progression for well differentiated cell line and circumstances for most original PTC cases.

Among our novel panel, half of them were previously identified in association with TC invasion and progression. For example, downregulated expression of FHL1 was associated with invasion of PTC.^49^ CTGF plays an essential role in PTC cell spheroids formation.^50^ LncRNA WT1 downregulates survivin by upregulating miR‐203 to inhibit PTC cell proliferation.^51^ Moreover, UHRF1 was highly expressed in human ATC cells, knockdown of UHRF1 inhibited proliferation and downregulated the expression of dedifferentiation marker CD97.^52^ All this evidence confirmed the intrinsic relationship between the novel 10‐gene signature and EMT‐induced aggressiveness of TC, although the roles of TGFBR3, FOXP2, FBLN5, IL11, WNT11, and AQP9in TC have not been reported previously. Thus, fundamental research focused on these genes may lead to a progress in the development of novel therapeutic targets. GO analysis indicated that DE‐ERGs were enriched in basement membrane, focal adhesion epithelial cell proliferation, extracellular matrix organization, and mesenchymal cell differentiation, which is consistent with the definition of ERGs. We assessed the potential turbulence caused by the novel ERG‐based signature by GSEA, which can help reflect the disturbance of the whole genome at the translational level better than general overrepresentation analysis (ORA).^53^ Multiple biological functions involved in several oncogenic and EMT‐related processes were found to be upregulated in the high‐risk group. These functions included process such as the cell adhesion molecules, chemokine signaling pathway, and keratinization, as well as the p53 and KRAS pathways. These findings partially explain the mechanism underlying the shorter PFI of cases with the higher risk score, although further studies are required for confirmation. During the establishment of stepwise regression model, we incorporated the risk score, clinical characters enrolled in multivariate analysis, and relevant characters previously reported significantly correlated with prognosis of PTC such RAS mutation status.^54^

Compared with the relatively low risk of cancer‐related death, recurrence or persistent disease remains the main issue that influence the prognosis of PTC patients.^55^ Reoperation results in a higher morbidity related to injury to the laryngeal nerve followed by vocal paralysis as the most serious complication.^56^ Personalized follow‐up based on precise prediction of recurrence risk after surgery would achieve a balance between excessive and insufficient management. The American Thyroid Association (ATA) published a risk stratification system (RSS) and response to therapy re‐classification (RTR), which has been widely employed for recurrence risk prediction.^57^ However, current prediction of recurrence risk is only possible after surgery or pathological examination.^58^ Furthermore, the complexity and diversity of the biological behavior of PTC are not taken into consideration, thus impeding early prognosis. Massive amounts of information obtained from high‐throughput methods, such as the multi‐omics technologies and next‐generating sequencing, have contributed tremendously to elucidation of the mechanism of TC.^59^, ^60^ Additionally, multiple novel classifiers have been identified for prognosis prediction from different gene sets focused on specified biological functions, which innovated us for the development of the novel classifier based on EMT database.^26^, ^61^

Some limitations of our study should be noted. First, due to the lack of array‐based independent gene expression profiling or sequence datasets with complete follow‐up information, we had to apply internal verification of our results. Thus, our findings require further validation using an external independent dataset. In addition, as compared to the 26.57% to 26.95% of CV in TCGA‐THCA cohort, a 10–20% CV for regular qRT‐PCR is considered more acceptable. At last, the primary source of our study was from TCGA program, in which the samples were collected from North American people. Possible biases would occur when applying the classifier in different countries or regions, which should be addressed in further clinical study.

## 5 CONCLUSION

In this study, we successfully identified a novel 10‐gene classifier and established a classifier‐based regression model combining relevant clinical characteristics for prediction of prognosis in PTC patients. The novel gene classifier and nomogram may be useful and convenient for personalized management for thyroid cancer.

## AUTHOR CONTRIBUTIONS

ZC and RL designed the study, carried out experiments, and obtained the data; RL, ZC, MP, and WM analyzed the data and conducted statistical analyses; RL, ZC, MP, and XL wrote the manuscript; HY and ZL revised and approved the manuscript.

## FUNDING INFORMATION

This research was supported by the National Natural Science Foundation of China (grant number: 82172727), the Nature Science Foundation of Beijing [grant number: 7202164], CAMS Innovation Fund for Medical Sciences (CIFMS) [grant number: 2016‐12 M‐3‐005], and CAMS Innovation Fund for graduate students [grant number: 2019–1002‐44].

## CONFLICT OF INTEREST

The authors declare that they have no competing interests.

## ETHICS STATEMENT

Not applicable

## Supporting information


**Appendix S1** Supplementary Figures

**Appendix S2** Supplementary Tables

## Data Availability

Data Availability Statement All the sequencing datas were obtained from the TCGA (https://portal.gdc.cancer.gov/) and the UCSC Xena database (https://xenabrowser.net/datapages/), the mutational datas were obtained from cBioPortal database (http://www.cbioportal.org/), the microarray datasets were obtained from GEO database (https://www.ncbi.nlm.nih.gov/geo/), other data that support the findings of this study are available from the corresponding author upon reasonable request.
