# Supplementary material for: A novel prognostic model for papillary thyroid cancer based on epithelial–mesenchymal transition‐related genes
Source: Cancer Med. 2022 May 24;11(23):4703–20. doi: 10.1002/cam4.4836 (PMC9741981; doi:10.1002/cam4.4836)
Supplement: Supplementary file 1 — Appendix S1 Supplementary Figures [file CAM4-11-4703-s002.docx]

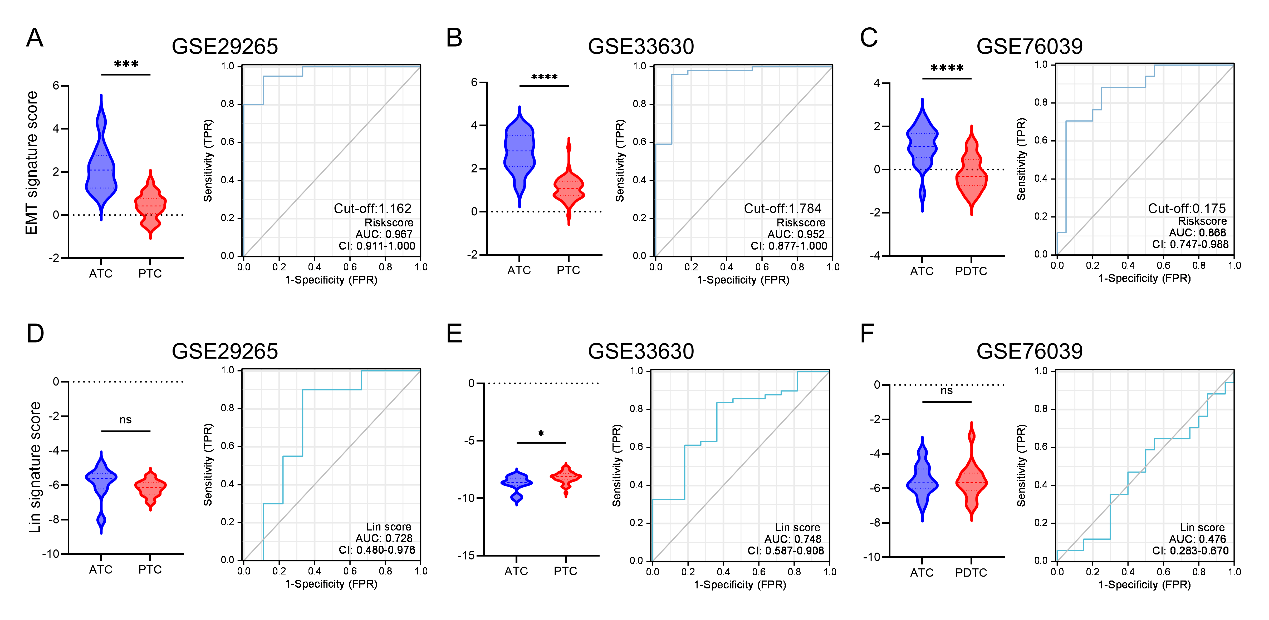
Supplementary Figure 1. The potential value of the EMT-signature in predicting PTC de-differentiation. Violin plot and ROC curve revealing the discriminative value of EMT signature. Signature score of samples from three GEO datasets (GSE 29265, GSE 33630, GSE 76039) were generated by EMT-signature (A-C) and Lin’s immune-signature (D-F). (A, D) Signature score of ATC versus PTC samples from GSE 29265. (B, E) Signature scores of ATC versus PTC samples from GSE 33630. (C, F) Signature score of ATC versus PDTC samples from GSE 76039. Data are presented as mean ± standard error of mean (SEM). Unpaired t test with Welch’s correction, *P < 0.05, **P < 0.01.


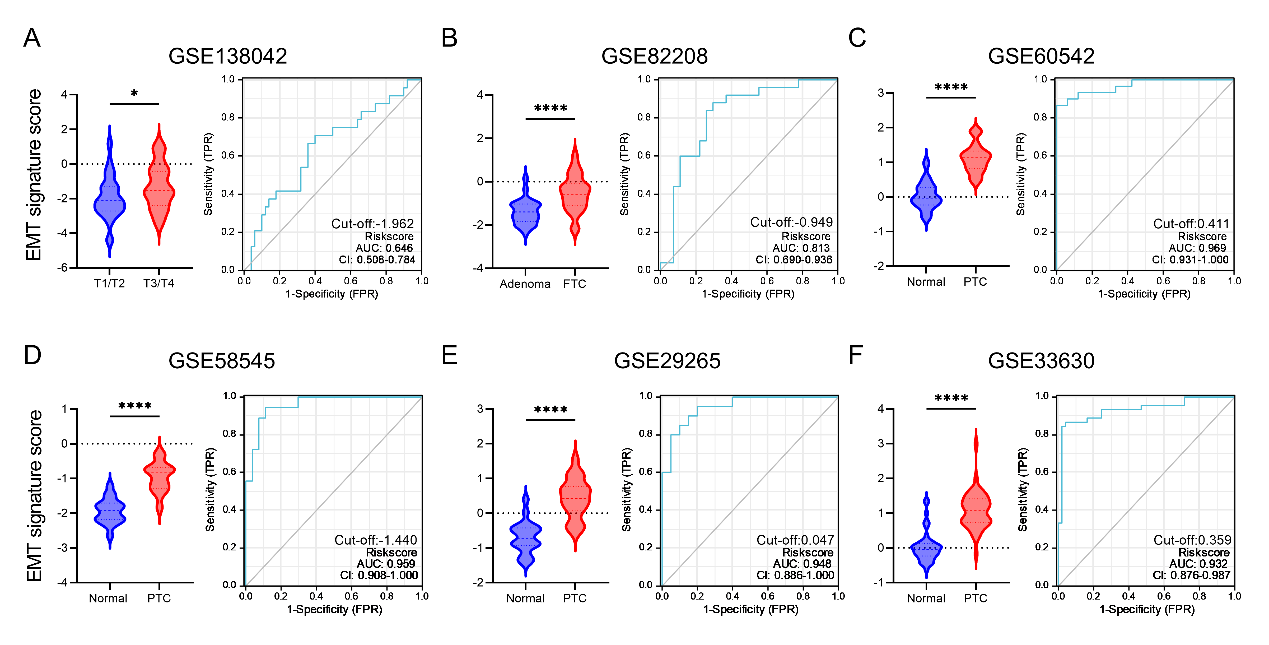
Supplementary Figure 2. External clinical validation of the EMT-signature. Violin plot and ROC curve revealing the discriminative value of EMT signature. (A) Signature scores of early-stage tumors (T1/T2) versus advanced tumors (T3/T4). (B) Signature score of follicular adenoma samples versus follicular cancer samples in GSE 82208. (C-F) Signature score of normal thyroid tissues versus PTC tissues in GSE60542, GSE 58545, GSE29265 and GSE 33630. Data are presented as mean ± standard error of mean (SEM). Unpaired t test with Welch’s correction, *P < 0.05, ****P < 0.0001.

Supplementary Figure 3. The online program for application of the nomogram available at: https://liuruisurgeon.shinyapps.io/EMTbased_nomogram_PTC/
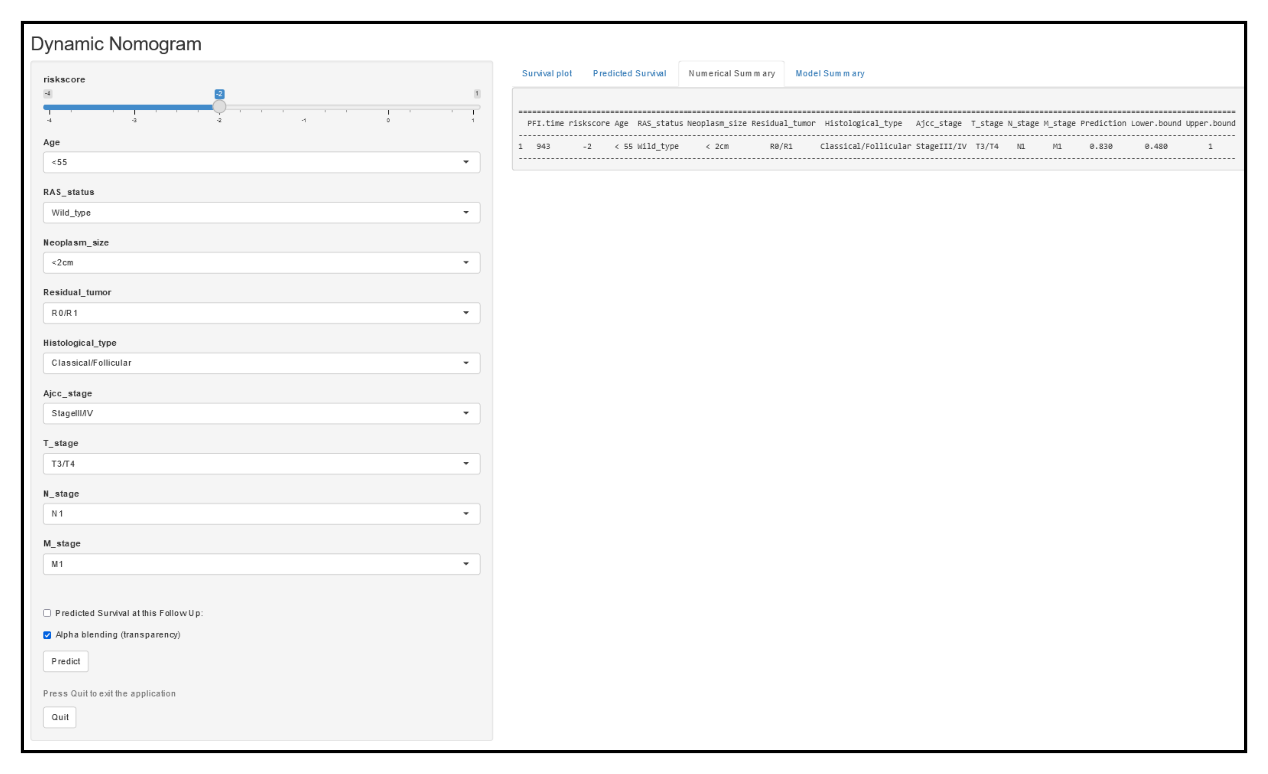
.

Supplementary document 1. Codes for analysis in the study

Code for random splits

library(survival)

setwd('')

THCA <- read.table(file = '.txt',header=T,sep="\t",row.names = 1,check.names = F )

as.data.frame(THCA)

str(THCA)

set.seed() #random number generator

ind <- sample(2, nrow(THCA), replace = T, prob = c(0.8, 0.2))

train <- THCA[ind==1, ] #the training data set

test <- THCA[ind==2, ] #the test data set

as.data.frame(train)

as.data.frame(test)

write.table(train, row.names=T,col.names = NA,file = 'train.txt',sep = '\t',quote=F)

write.table(test, row.names=T,col.names = NA,file = 'test.txt',sep = '\t',quote=F)

Code for edgeR differential analysis

setwd()

library("edgeR")

foldChange=1

padj=0.05

rt=read.table(".txt",sep="\t",header=T,check.names=F)

rt=as.matrix(rt)

rownames(rt)=rt[,1]

exp=rt[,2:ncol(rt)]

dimnames=list(rownames(exp),colnames(exp))

data=matrix(as.numeric(as.matrix(exp)),nrow=nrow(exp),dimnames=dimnames)

data=avereps(data)

data=data[rowMeans(data)>0,]

#group=c("normal","tumor","tumor","normal","tumor")

group=c(rep("normal",),rep("tumor",))

design <- model.matrix(~group)

y <- DGEList(counts=data,group=group)

y <- calcNormFactors(y)

y <- estimateCommonDisp(y)

y <- estimateTagwiseDisp(y)

et <- exactTest(y,pair = c("normal","tumor"))

topTags(et)

ordered_tags <- topTags(et, n=100000)

allDiff=ordered_tags$table

allDiff=allDiff[is.na(allDiff$FDR)==FALSE,]

diff=allDiff

newData=y$pseudo.counts

write.table(diff,file=".xls",sep="\t",quote=F)

diffSig = diff[(diff$FDR < padj & (diff$logFC>foldChange | diff$logFC<(-foldChange))),]

write.table(diffSig, file=".xls",sep="\t",quote=F)

diffUp = diff[(diff$FDR < padj & (diff$logFC>foldChange)),]

write.table(diffUp, file=".xls",sep="\t",quote=F)

diffDown = diff[(diff$FDR < padj & (diff$logFC<(-foldChange))),]

write.table(diffDown, file=".xls",sep="\t",quote=F)

normalizeExp=rbind(id=colnames(newData),newData)

write.table(normalizeExp,file=".txt",sep="\t",quote=F,col.names=F)

diffExp=rbind(id=colnames(newData),newData[rownames(diffSig),])

write.table(diffExp,file=".txt",sep="\t",quote=F,col.names=F)#

Code for LASSO analysis

library("glmnet")

library("survival")

setwd('')

rt=read.table(".txt",header=T,sep="",row.names=1)

rt$PFI.time=rt$PFI.time/365

set.seed(1)

x=as.matrix(rt[,c(3:ncol(rt))])

y=data.matrix(Surv(rt$PFI.time,rt$PFI))

fit=glmnet(x, y, family = "cox")

plot(fit, xvar = "lambda", label = TRUE)

cvfit = cv.glmnet(x, y, family="cox")

plot(cvfit)

cvfit

abline(v = log(c(cvfit$lambda.min,cvfit$lambda.1se)),lty="dashed")

coef = coef(fit, s = cvfit$lambda.min)

index = which(coef != 0)

actCoef = coef[index]

lassoGene = row.names(coef)[index]

geneCoef = cbind(Gene=lassoGene,Coef=actCoef)

geneCoef

FinalGeneExp = rt[,lassoGene]

myFun = function(x){crossprod(as.numeric(x),actCoef)}

riskScore = apply(FinalGeneExp,1,myFun)

outCol = c("PFI.time", "PFI", lassoGene)

risk = as.vector(ifelse(riskScore > median(riskScore), "high", "low"))

dat = cbind(rt[,outCol], riskScore=as.vector(riskScore), risk)

library(ggpubr)

p <- ggboxplot(dat, x = "PFI", y = "riskScore",

color = "PFI", palette = "jco",

add = "jitter")

p <- p + stat_compare_means() # Add p-value

p

library(ROCR)

library(glmnet)

library(caret)

pred <- prediction(dat$riskScore, dat$PFI)

perf <- performance(pred,"tpr","fpr")

performance(pred,"auc") # shows calculated AUC for model

plot(perf,colorize=FALSE, col="red")

lines(c(0,1),c(0,1),col = "gray", lty = 4 )

auc = performance(pred,"auc")

auc = unlist(slot(auc, "y.values"))

auc

write.table(riskScore,file = '.txt',sep = '\t',row.names = TRUE,

col.names = TRUE,quote = F)

rt=read.table("test.txt",header=T,sep="",row.names=1)

rt$PFI.time=rt$PFI.time/365

FinalGeneExp = rt[,lassoGene]

myFun = function(x){crossprod(as.numeric(x),actCoef)}

riskScore = apply(FinalGeneExp,1,myFun)

outCol = c("PFI.time", "PFI", lassoGene)

risk = as.vector(ifelse(riskScore > median(riskScore), "high", "low"))

dat = cbind(rt[,outCol], riskScore=as.vector(riskScore), risk)

library(ggpubr)

p <- ggboxplot(dat, x = "PFI", y = "riskScore",

color = "PFI", palette = "jco",

add = "jitter")

p <- p + stat_compare_means()

p

library(ROCR)

library(glmnet)

library(caret)

pred <- prediction(dat$riskScore, dat$PFI)

perf <- performance(pred,"tpr","fpr")

performance(pred,"auc")

plot(perf,colorize=FALSE, col="red")

lines(c(0,1),c(0,1),col = "gray", lty = 4 )

auc = performance(pred,"auc")

auc = unlist(slot(auc, "y.values"))

auc

write.csv(riskScore,file = '.csv')

library('timeROC')

library(survival)

setwd('')

library(survcomp)

THCA <- read.table(".txt",header=T,sep="\t")

fit <- coxph(Surv(PFI.time,PFI)~risk_score, data=THCA)

cindex <- concordance.index(predict(fit),

surv.time = THCA$PFI.time, surv.event = THCA$PFI,method = "noether")

cindex$c.index; cindex$lower; cindex$upper

Code for Cox survival analysis

library(survival)

setwd('')

inputfile=".txt"

gene<-read.table(inputfile,header=T,sep="\t",row.names = 1,check.names = F)

coxR=data.frame()

coxf<-function(x){

fmla1 <- as.formula(Surv(PFI_time,status)~gene[,x])

mycox <- coxph(fmla1,data=gene)

}

for(a in colnames(gene[,3:ncol(gene)])){

mycox=coxf(a)

coxResult = summary(mycox)

coxR=rbind(coxR,cbind(genename=a,HR=coxResult$coefficients[,"exp(coef)"],

P=coxResult$coefficients[,"Pr(>|z|)"]))

}

write.table(coxR,"coxResult.txt",sep="\t",row.names=F,quote=F)

Code for GO and KEGG analysis

library(clusterProfiler)

library(org.Hs.eg.db) ## org.Mm.eg.db

gene_ids = bitr(geneID = gene_list, fromType = "SYMBOL",

toType = "ENTREZID", OrgDb = "org.Hs.eg.db")

ego <- enrichGO(gene = gene_ids$ENTREZID, OrgDb = "org.Hs.eg.db",

keyType = "ENTREZID", ont = "BP")

Code for Cox regression and nomogram visualization

library(foreign)

library(rms)

library(survival)

library(regplot)

library('DynNom')

setwd('')

THCA <- read.table(".txt",header=T,sep="\t")

THCA<-as.data.frame(THCA)

str(THCA)

dd <- datadist(THCA)

option <- options(datadist = "dd")

coxm <- cph(Surv(PFI.time,PFI) ~ Signature.score+Age+T+

N+M+Histological.type,data = THCA, x = T, y = T, surv = T)

surv <- Survival(coxm)

res.cox <- psm(Surv(PFI.time,PFI) ~ Signature.score+Age+T+

N+M+Histological.type+Primary.type+~, data = THCA,dist='lognormal',x=T, y=T)

surv <- Survival(res.cox)

nom.cox <- nomogram(res.cox,

fun=list(function(x) surv(365, x),function(x) surv(730, x),function(x) surv(1095, x),

function(x) surv(1825, x)),

funlabel=c("1-year PFI","3-year PFI", "5-year PFI"),

maxscale=10,

fun.at=c(0.01,seq(0.1,0.9,by=0.2),0.95,0.99))

plot((nom.cox), xfrac = .3)

surv <- Survival(coxm)

nom <- nomogram(coxm,fun=list(function(x)surv(365, x), function(x)surv(1095, x),

function(x)surv(1825, x)),

lp = T,funlabel = c('1-Year PFI', '3-Years PFI','5-Years PFI'),

maxscale = 10, fun.at = c('0.90','0.80','0.70','0.6','0.5','0.4','0.3','0.2','0.1'))

plot((nom), xfrac = .3)

nom1<-regplot(nom.cox, clickable=TRUE,

points=TRUE, rank="sd",failtime = c(365,1095,1825),prfail = T)

cal_5<-calibrate(res.cox,u=1825,method="boot",cmethod='KM',m=65,B=1000)

par(mar=c(7,4,4,3),cex=1.0)

plot(cal_5,lwd=2,lty=2,

errbar.col=c(rgb(100,18,192,maxColorValue = 255)),

xlab='Nomogram-Predicted Probability of 5-year PFI',

ylab='Actual 5-year PFI(proportion)',

col=c(rgb(142,18,177,maxColorValue = 255)),

xlim = c(0.5,1),ylim = c(0,1))

library('nomogramFormula')

options(option)

results <- formula_lp(nomogram = nom.cox)

points <- points_cal(formula = results$formula, lp = nom.cox$linear.predictors)

head(points)

options(option)

results <- formula_rd(nomogram = nom.cox)

THCA$points <- points_cal(formula = results$formula,rd=THCA)

head(THCA$points)

THCA$linear.predictors<-predict(res.cox,type="lp",newdata=THCA)

head(THCA$linear.predictors)

library('pec')

THCA$survprob1 <- predictSurvProb(res.cox,newdata=THCA,times=365)

THCA$survprob3 <- predictSurvProb(res.cox,newdata=THCA,times=1025)

THCA$survprob5 <- predictSurvProb(res.cox,newdata=THCA,times=1825)

write.csv(THCA, file = ".csv")

print(surv)

print(nom)

print(coxm)

fcox <- cph(Surv(PFI.time, PFI) ~ Gene.score+Age+T+

N+M+Histological.type+Primary.type,data = THCA, x = T, y = T, surv = T)

DynNom(fcox, THCA)

DNbuilder(fcox)

| **Table S1. Top 500 DEGs identified in PTC with edgeR** | | | | | |
| --- | --- | --- | --- | --- | --- |
| Genes | logFC | logCPM | PValue | FDR | Regulated |
| METTL7B | 4.362161 | 5.866525 | 3.98E-82 | 2.32E-77 | Up-Regulated |
| GALE | 3.238034 | 6.050589 | 3.27E-76 | 1.91E-71 | Up-Regulated |
| P4HA2 | 2.044891 | 6.360803 | 9.49E-76 | 5.54E-71 | Up-Regulated |
| SRCIN1 | 3.441774 | 4.407189 | 1.99E-75 | 1.16E-70 | Up-Regulated |
| ETV4 | 3.435957 | 5.456567 | 1.30E-74 | 7.58E-70 | Up-Regulated |
| PLXND1 | 1.65252 | 7.426605 | 3.28E-74 | 1.91E-69 | Up-Regulated |
| GABRB2 | 7.189509 | 6.507562 | 4.47E-74 | 2.61E-69 | Up-Regulated |
| GOLT1A | 4.00799 | 3.403701 | 3.13E-73 | 1.83E-68 | Up-Regulated |
| PRR36 | 3.307444 | 4.325133 | 1.77E-71 | 1.03E-66 | Up-Regulated |
| LRP4 | 4.52967 | 7.704609 | 9.40E-71 | 5.49E-66 | Up-Regulated |
| STK32A | 2.972476 | 5.968177 | 7.21E-70 | 4.21E-65 | Up-Regulated |
| SHROOM4 | 2.827807 | 6.01597 | 1.56E-69 | 9.11E-65 | Up-Regulated |
| LIPH | 5.690439 | 5.954686 | 8.42E-69 | 4.92E-64 | Up-Regulated |
| BMP1 | 1.591066 | 6.399493 | 2.06E-68 | 1.20E-63 | Up-Regulated |
| ENTPD1 | 2.559727 | 7.860126 | 4.13E-68 | 2.41E-63 | Up-Regulated |
| TUSC3 | 3.222503 | 5.989115 | 6.39E-68 | 3.73E-63 | Up-Regulated |
| KLHDC8A | 4.900204 | 5.519689 | 2.45E-67 | 1.43E-62 | Up-Regulated |
| PLEKHN1 | 3.695581 | 2.897945 | 8.51E-67 | 4.96E-62 | Up-Regulated |
| RP11-363E7.4 | 1.856158 | 3.319047 | 2.99E-66 | 1.75E-61 | Up-Regulated |
| NPC2 | 2.578499 | 10.90092 | 3.72E-66 | 2.17E-61 | Up-Regulated |
| RP11-353N14.2 | 3.883144 | 0.41405 | 9.76E-66 | 5.69E-61 | Up-Regulated |
| ZMAT3 | 1.92634 | 6.518452 | 1.42E-65 | 8.30E-61 | Up-Regulated |
| CDH3 | 4.145565 | 6.73317 | 2.36E-65 | 1.37E-60 | Up-Regulated |
| ABR | 1.572793 | 6.742246 | 3.23E-65 | 1.88E-60 | Up-Regulated |
| BBC3 | 2.194537 | 3.902327 | 4.51E-65 | 2.63E-60 | Up-Regulated |
| ETV5 | 1.873517 | 6.390929 | 7.96E-65 | 4.64E-60 | Up-Regulated |
| PTCHD4 | 3.398757 | 4.204524 | 1.02E-64 | 5.92E-60 | Up-Regulated |
| SERINC2 | 1.913155 | 7.28144 | 1.90E-64 | 1.11E-59 | Up-Regulated |
| MPZL2 | 2.499199 | 7.954431 | 5.65E-64 | 3.30E-59 | Up-Regulated |
| FAXC | 3.189691 | 3.836334 | 8.87E-64 | 5.18E-59 | Up-Regulated |
| ABCC6P2 | 2.36121 | 0.52618 | 3.68E-63 | 2.15E-58 | Up-Regulated |
| GALNT7 | 2.326815 | 6.539133 | 1.98E-62 | 1.16E-57 | Up-Regulated |
| GRHL3 | 5.091102 | 2.477705 | 2.23E-62 | 1.30E-57 | Up-Regulated |
| CCND1 | 1.903563 | 8.91707 | 2.51E-62 | 1.46E-57 | Up-Regulated |
| RUNDC3A-AS1 | 2.013164 | 2.778259 | 8.26E-62 | 4.82E-57 | Up-Regulated |
| AP000997.3 | 3.762039 | -1.61759 | 1.50E-61 | 8.76E-57 | Up-Regulated |
| DLG4 | 1.599813 | 5.082165 | 1.99E-60 | 1.16E-55 | Up-Regulated |
| TGFA | 2.671022 | 6.437983 | 2.01E-60 | 1.17E-55 | Up-Regulated |
| PHLDA3 | 2.041393 | 6.215376 | 2.57E-60 | 1.50E-55 | Up-Regulated |
| SAMD1 | 1.287368 | 5.369585 | 5.53E-60 | 3.23E-55 | Up-Regulated |
| SLC22A31 | 7.17885 | 5.567438 | 1.01E-59 | 5.90E-55 | Up-Regulated |
| NRCAM | 2.402187 | 5.900781 | 1.34E-59 | 7.80E-55 | Up-Regulated |
| EPS8 | 2.084239 | 6.945373 | 1.34E-59 | 7.84E-55 | Up-Regulated |
| ECE1 | 1.683082 | 9.080848 | 1.61E-59 | 9.39E-55 | Up-Regulated |
| RNF24 | 1.36583 | 5.839745 | 2.03E-59 | 1.19E-54 | Up-Regulated |
| HMGA2 | 5.607711 | 5.49363 | 1.01E-58 | 5.86E-54 | Up-Regulated |
| FAM84A | 2.985598 | 4.95213 | 1.37E-58 | 8.01E-54 | Up-Regulated |
| COL13A1 | 3.495201 | 3.971314 | 2.06E-58 | 1.20E-53 | Up-Regulated |
| NGEF | 5.350493 | 5.241501 | 3.61E-58 | 2.11E-53 | Up-Regulated |
| PTP4A3 | 2.019687 | 5.605357 | 4.37E-58 | 2.54E-53 | Up-Regulated |
| KCNQ3 | 4.561961 | 4.902924 | 5.32E-58 | 3.10E-53 | Up-Regulated |
| PLCD3 | 3.501329 | 6.953777 | 6.76E-58 | 3.94E-53 | Up-Regulated |
| FHOD1 | 1.533528 | 5.918199 | 2.28E-57 | 1.33E-52 | Up-Regulated |
| JAG2 | 1.555012 | 4.915168 | 3.14E-57 | 1.83E-52 | Up-Regulated |
| RP11-221N13.3 | 4.642438 | 0.871485 | 4.03E-57 | 2.35E-52 | Up-Regulated |
| TENM1 | 4.67923 | 6.2606 | 4.49E-57 | 2.62E-52 | Up-Regulated |
| RXRG | 5.218568 | 6.353714 | 5.02E-57 | 2.93E-52 | Up-Regulated |
| NOL4L | 1.017212 | 5.368269 | 7.12E-57 | 4.15E-52 | Up-Regulated |
| SEMA3F | 1.599272 | 6.484425 | 7.55E-57 | 4.40E-52 | Up-Regulated |
| RPSAP52 | 4.346658 | 0.394329 | 9.68E-57 | 5.64E-52 | Up-Regulated |
| CTC-255N20.1 | 3.102305 | 1.032866 | 1.32E-56 | 7.70E-52 | Up-Regulated |
| TNFRSF10C | 2.49248 | 3.591252 | 1.70E-56 | 9.91E-52 | Up-Regulated |
| UNC5B | 2.288692 | 5.796102 | 2.98E-56 | 1.74E-51 | Up-Regulated |
| TNFRSF10B | 1.210223 | 6.118625 | 3.64E-56 | 2.12E-51 | Up-Regulated |
| ADORA1 | 2.806677 | 5.262827 | 4.95E-56 | 2.88E-51 | Up-Regulated |
| RP11-547D24.1 | 3.509402 | -1.00168 | 5.66E-56 | 3.30E-51 | Up-Regulated |
| TMEM41A | 1.092799 | 5.066318 | 1.43E-55 | 8.36E-51 | Up-Regulated |
| DDB2 | 1.429423 | 4.842981 | 2.12E-55 | 1.24E-50 | Up-Regulated |
| MYEF2 | 1.589146 | 4.351618 | 2.40E-55 | 1.40E-50 | Up-Regulated |
| PVRL4 | 4.737556 | 4.801074 | 9.23E-55 | 5.38E-50 | Up-Regulated |
| LPAR5 | 3.478227 | 5.746083 | 1.10E-54 | 6.40E-50 | Up-Regulated |
| KIAA1211L | 1.139069 | 4.525872 | 3.39E-54 | 1.97E-49 | Up-Regulated |
| HEY2 | 2.19054 | 4.170565 | 3.99E-54 | 2.32E-49 | Up-Regulated |
| PRR15 | 5.82979 | 6.732471 | 4.52E-54 | 2.63E-49 | Up-Regulated |
| RP11-230G5.2 | 5.435039 | 1.185252 | 6.12E-54 | 3.57E-49 | Up-Regulated |
| CBX2 | 1.475706 | 1.887047 | 6.90E-54 | 4.02E-49 | Up-Regulated |
| MAP3K6 | 1.488491 | 5.505741 | 1.00E-53 | 5.85E-49 | Up-Regulated |
| MTHFD1L | 1.424898 | 4.899558 | 1.37E-53 | 7.99E-49 | Up-Regulated |
| RP3-510D11.2 | 1.710414 | 0.847784 | 2.15E-53 | 1.25E-48 | Up-Regulated |
| FAM178B | 4.962526 | 1.348831 | 2.26E-53 | 1.32E-48 | Up-Regulated |
| HCN4 | 4.680304 | 2.205403 | 3.02E-53 | 1.76E-48 | Up-Regulated |
| REN | 5.199564 | 0.864361 | 4.45E-53 | 2.59E-48 | Up-Regulated |
| BID | 2.035649 | 5.055987 | 5.82E-53 | 3.39E-48 | Up-Regulated |
| DPP4 | 4.577917 | 7.142287 | 6.05E-53 | 3.52E-48 | Up-Regulated |
| PROS1 | 3.600447 | 8.032906 | 9.75E-53 | 5.68E-48 | Up-Regulated |
| AC079630.2 | 5.353789 | 3.372323 | 1.20E-52 | 7.01E-48 | Up-Regulated |
| CDKN2B | 3.02647 | 3.367818 | 1.66E-52 | 9.65E-48 | Up-Regulated |
| QPCT | 3.522445 | 6.16403 | 1.72E-52 | 1.00E-47 | Up-Regulated |
| GGCT | 2.275919 | 6.14822 | 8.58E-52 | 5.00E-47 | Up-Regulated |
| ADK | 1.117488 | 5.94107 | 1.17E-51 | 6.80E-47 | Up-Regulated |
| SLMO1 | 2.071065 | 2.378443 | 1.22E-51 | 7.13E-47 | Up-Regulated |
| SPINT1 | 1.751781 | 7.953812 | 1.31E-51 | 7.64E-47 | Up-Regulated |
| UNC5B-AS1 | 5.027585 | 0.09165 | 1.33E-51 | 7.76E-47 | Up-Regulated |
| SPTBN2 | 3.626293 | 4.155478 | 1.42E-51 | 8.28E-47 | Up-Regulated |
| RP11-350J20.12 | 4.566807 | -0.12368 | 2.20E-51 | 1.28E-46 | Up-Regulated |
| LIMK1 | 1.199802 | 6.190462 | 3.78E-51 | 2.20E-46 | Up-Regulated |
| RASD2 | 3.171068 | 4.378972 | 4.48E-51 | 2.61E-46 | Up-Regulated |
| GLT1D1 | 2.746883 | 3.713042 | 7.71E-51 | 4.49E-46 | Up-Regulated |
| LRRK2 | 4.057065 | 8.049773 | 1.06E-50 | 6.18E-46 | Up-Regulated |
| LAD1 | 2.615008 | 5.490346 | 1.19E-50 | 6.91E-46 | Up-Regulated |
| AGRN | 1.207838 | 8.83764 | 1.45E-50 | 8.42E-46 | Up-Regulated |
| CDH24 | 1.447866 | 3.455202 | 1.51E-50 | 8.76E-46 | Up-Regulated |
| ULBP2 | 2.875374 | 2.004692 | 2.61E-50 | 1.52E-45 | Up-Regulated |
| GJB3 | 4.806562 | 3.971789 | 2.98E-50 | 1.73E-45 | Up-Regulated |
| TRIM47 | 1.637562 | 5.232076 | 3.14E-50 | 1.83E-45 | Up-Regulated |
| CELF4 | 3.309507 | 2.251373 | 3.67E-50 | 2.13E-45 | Up-Regulated |
| MANEAL | 1.852265 | 3.890383 | 4.09E-50 | 2.38E-45 | Up-Regulated |
| B3GNT7 | 2.63809 | 4.309966 | 4.93E-50 | 2.87E-45 | Up-Regulated |
| RP11-467J12.4 | 2.563253 | -2.34187 | 4.95E-50 | 2.88E-45 | Up-Regulated |
| ABCC6P1 | 3.105761 | 0.797793 | 5.22E-50 | 3.04E-45 | Up-Regulated |
| SPATA9 | 1.71842 | 0.00701 | 5.70E-50 | 3.31E-45 | Up-Regulated |
| SCEL | 4.23543 | 6.084027 | 6.25E-50 | 3.64E-45 | Up-Regulated |
| TYRO3 | 2.068194 | 3.793499 | 9.59E-50 | 5.58E-45 | Up-Regulated |
| ZCCHC12 | 6.814225 | 8.927407 | 1.09E-49 | 6.35E-45 | Up-Regulated |
| UBE2QL1 | 2.558623 | 4.372625 | 1.67E-49 | 9.71E-45 | Up-Regulated |
| ST3GAL5 | 1.517598 | 7.717172 | 1.96E-49 | 1.14E-44 | Up-Regulated |
| SYTL5 | 5.856372 | 4.527297 | 2.61E-49 | 1.52E-44 | Up-Regulated |
| UBTD1 | 1.200709 | 4.901258 | 3.68E-49 | 2.14E-44 | Up-Regulated |
| RYR1 | 3.049843 | 5.062576 | 5.11E-49 | 2.97E-44 | Up-Regulated |
| ALDH3B1 | 2.689158 | 5.283428 | 5.46E-49 | 3.18E-44 | Up-Regulated |
| XPR1 | 1.705844 | 6.946348 | 6.61E-49 | 3.84E-44 | Up-Regulated |
| SCN1B | 2.452613 | 4.472159 | 6.98E-49 | 4.06E-44 | Up-Regulated |
| C16orf45 | 1.845454 | 5.268569 | 1.26E-48 | 7.31E-44 | Up-Regulated |
| EPHB3 | 3.599485 | 4.374055 | 1.69E-48 | 9.86E-44 | Up-Regulated |
| ARHGAP23 | 1.862449 | 5.726571 | 2.33E-48 | 1.36E-43 | Up-Regulated |
| CDH6 | 3.440311 | 5.536253 | 2.60E-48 | 1.51E-43 | Up-Regulated |
| NPDC1 | 1.827888 | 6.385342 | 2.77E-48 | 1.61E-43 | Up-Regulated |
| RP11-280O1.2 | 7.142086 | 3.698278 | 2.85E-48 | 1.66E-43 | Up-Regulated |
| PERM1 | 3.315763 | 1.79624 | 4.72E-48 | 2.75E-43 | Up-Regulated |
| TMEM8A | 1.019732 | 6.584225 | 6.52E-48 | 3.79E-43 | Up-Regulated |
| CTC-277H1.7 | 2.141224 | 0.653203 | 6.81E-48 | 3.96E-43 | Up-Regulated |
| C11orf80 | 1.708425 | 3.985658 | 1.47E-47 | 8.54E-43 | Up-Regulated |
| CAMK2N2 | 3.732101 | -0.05149 | 2.00E-47 | 1.17E-42 | Up-Regulated |
| ELFN1 | 1.972537 | 1.381952 | 3.54E-47 | 2.06E-42 | Up-Regulated |
| SERPINA1 | 4.696984 | 10.66217 | 3.78E-47 | 2.20E-42 | Up-Regulated |
| STRA6 | 5.475205 | 4.735752 | 3.83E-47 | 2.23E-42 | Up-Regulated |
| GDF15 | 4.267353 | 6.594138 | 5.70E-47 | 3.32E-42 | Up-Regulated |
| RIN1 | 2.428047 | 3.022166 | 6.05E-47 | 3.52E-42 | Up-Regulated |
| GRB7 | 2.345583 | 5.165962 | 6.46E-47 | 3.76E-42 | Up-Regulated |
| CTXN1 | 3.428038 | 4.181183 | 6.88E-47 | 4.00E-42 | Up-Regulated |
| TIMM8AP1 | 4.70195 | 0.392344 | 7.75E-47 | 4.51E-42 | Up-Regulated |
| LRRC75A | 1.518471 | 3.979911 | 9.65E-47 | 5.61E-42 | Up-Regulated |
| AC018816.3 | 3.15441 | 1.650417 | 1.10E-46 | 6.39E-42 | Up-Regulated |
| NOX4 | 2.607519 | 2.70955 | 1.64E-46 | 9.51E-42 | Up-Regulated |
| TPD52L1 | 1.911552 | 7.077045 | 1.71E-46 | 9.93E-42 | Up-Regulated |
| MFGE8 | 2.378334 | 7.9827 | 1.79E-46 | 1.04E-41 | Up-Regulated |
| MRC2 | 3.165641 | 7.578052 | 2.02E-46 | 1.17E-41 | Up-Regulated |
| DRAXIN | 3.658287 | 1.693026 | 2.23E-46 | 1.30E-41 | Up-Regulated |
| MCTP2 | 1.292899 | 5.582525 | 3.38E-46 | 1.96E-41 | Up-Regulated |
| TIAM1 | 3.074294 | 5.455231 | 3.78E-46 | 2.20E-41 | Up-Regulated |
| MTMR11 | 2.280652 | 3.570402 | 5.59E-46 | 3.25E-41 | Up-Regulated |
| AC079630.4 | 3.991975 | 4.632479 | 6.45E-46 | 3.75E-41 | Up-Regulated |
| KCNJ2 | 3.925339 | 5.010422 | 6.98E-46 | 4.06E-41 | Up-Regulated |
| SEMA4C | 1.163852 | 5.517179 | 7.95E-46 | 4.62E-41 | Up-Regulated |
| SYTL1 | 2.006749 | 5.976436 | 8.15E-46 | 4.74E-41 | Up-Regulated |
| PDGFA | 1.368577 | 3.732757 | 1.02E-45 | 5.93E-41 | Up-Regulated |
| DYSF | 1.49873 | 5.723791 | 1.35E-45 | 7.83E-41 | Up-Regulated |
| EPHA10 | 4.216176 | 3.223444 | 1.38E-45 | 8.04E-41 | Up-Regulated |
| PDE4C | 3.54483 | 4.151463 | 1.59E-45 | 9.23E-41 | Up-Regulated |
| CITED1 | 4.972283 | 7.751085 | 1.65E-45 | 9.59E-41 | Up-Regulated |
| LRP4-AS1 | 4.258732 | -0.13216 | 1.73E-45 | 1.01E-40 | Up-Regulated |
| INSR | 1.163341 | 7.074943 | 1.87E-45 | 1.09E-40 | Up-Regulated |
| KRT15 | 5.109916 | 2.549667 | 2.24E-45 | 1.30E-40 | Up-Regulated |
| PLD3 | 1.314909 | 8.778495 | 2.63E-45 | 1.53E-40 | Up-Regulated |
| CAMK2N1 | 3.47604 | 7.12611 | 2.63E-45 | 1.53E-40 | Up-Regulated |
| RP1-228H13.5 | 1.712494 | 1.740922 | 2.78E-45 | 1.61E-40 | Up-Regulated |
| HPCAL4 | 5.23074 | 4.137618 | 2.81E-45 | 1.63E-40 | Up-Regulated |
| RP11-320N7.2 | 3.288168 | 0.380649 | 3.77E-45 | 2.19E-40 | Up-Regulated |
| FN1 | 5.75278 | 13.70144 | 4.23E-45 | 2.46E-40 | Up-Regulated |
| MET | 2.722577 | 8.791319 | 4.44E-45 | 2.58E-40 | Up-Regulated |
| DUSP6 | 2.483869 | 8.668514 | 6.44E-45 | 3.74E-40 | Up-Regulated |
| TGFB1I1 | 1.136199 | 5.161206 | 7.36E-45 | 4.28E-40 | Up-Regulated |
| GABRD | 3.074039 | 2.796921 | 9.07E-45 | 5.27E-40 | Up-Regulated |
| RP11-474O21.5 | 4.645983 | 3.086902 | 9.33E-45 | 5.42E-40 | Up-Regulated |
| TSTA3 | 1.046306 | 6.13864 | 1.11E-44 | 6.42E-40 | Up-Regulated |
| PLA2G12B | 5.744848 | 1.257273 | 1.26E-44 | 7.33E-40 | Up-Regulated |
| DTX4 | 3.220995 | 8.433002 | 1.45E-44 | 8.42E-40 | Up-Regulated |
| LAMB3 | 4.597858 | 7.386276 | 1.50E-44 | 8.72E-40 | Up-Regulated |
| CCND2 | 1.430832 | 8.41649 | 1.85E-44 | 1.07E-39 | Up-Regulated |
| CORO2A | 2.012086 | 4.548276 | 1.89E-44 | 1.10E-39 | Up-Regulated |
| RARA | 1.03939 | 5.609296 | 2.37E-44 | 1.38E-39 | Up-Regulated |
| SLC35F2 | 1.734011 | 5.125602 | 2.38E-44 | 1.38E-39 | Up-Regulated |
| CDH13 | 2.099056 | 4.33031 | 4.44E-44 | 2.58E-39 | Up-Regulated |
| BAIAP3 | 3.559062 | 4.422003 | 6.04E-44 | 3.51E-39 | Up-Regulated |
| ICAM5 | 3.904832 | 4.058038 | 7.53E-44 | 4.37E-39 | Up-Regulated |
| RAB27A | 1.86793 | 7.231698 | 1.05E-43 | 6.09E-39 | Up-Regulated |
| FYN | 1.308859 | 7.243306 | 1.33E-43 | 7.69E-39 | Up-Regulated |
| C1orf216 | 1.057429 | 4.088222 | 1.63E-43 | 9.44E-39 | Up-Regulated |
| ARHGEF2 | 1.023695 | 6.88898 | 1.92E-43 | 1.12E-38 | Up-Regulated |
| TGFB1 | 1.543023 | 6.078809 | 1.93E-43 | 1.12E-38 | Up-Regulated |
| B3GNT8 | 2.266812 | 2.631122 | 2.16E-43 | 1.26E-38 | Up-Regulated |
| ISYNA1 | 1.663757 | 5.934366 | 2.20E-43 | 1.28E-38 | Up-Regulated |
| CLDN16 | 5.198337 | 6.57721 | 2.29E-43 | 1.33E-38 | Up-Regulated |
| TMEM184A | 3.040352 | 3.309807 | 2.45E-43 | 1.42E-38 | Up-Regulated |
| AMIGO2 | 2.569197 | 5.018021 | 2.59E-43 | 1.50E-38 | Up-Regulated |
| RAPGEF5 | 1.625518 | 4.98732 | 2.73E-43 | 1.59E-38 | Up-Regulated |
| DVL1 | 1.1116 | 6.717575 | 3.38E-43 | 1.96E-38 | Up-Regulated |
| TMPRSS6 | 7.16024 | 5.728211 | 3.40E-43 | 1.97E-38 | Up-Regulated |
| PDE9A | 1.779425 | 4.993067 | 3.72E-43 | 2.16E-38 | Up-Regulated |
| RIPPLY3 | 2.170267 | -0.54087 | 3.75E-43 | 2.18E-38 | Up-Regulated |
| RP11-615I2.2 | 2.437491 | 0.111583 | 6.12E-43 | 3.55E-38 | Up-Regulated |
| SDC4 | 2.482658 | 9.772335 | 6.24E-43 | 3.62E-38 | Up-Regulated |
| ABCC3 | 2.691879 | 6.402346 | 7.68E-43 | 4.46E-38 | Up-Regulated |
| HNRNPA1P27 | 4.261568 | 1.340114 | 1.08E-42 | 6.28E-38 | Up-Regulated |
| SEZ6L2 | 3.977771 | 5.106661 | 1.17E-42 | 6.81E-38 | Up-Regulated |
| ADRA1B | 2.306688 | 1.97391 | 1.25E-42 | 7.25E-38 | Up-Regulated |
| C19orf26 | 2.024535 | 1.098482 | 1.41E-42 | 8.17E-38 | Up-Regulated |
| PSD3 | 2.008408 | 6.67985 | 1.45E-42 | 8.39E-38 | Up-Regulated |
| MAMLD1 | 2.916075 | 4.18563 | 1.77E-42 | 1.03E-37 | Up-Regulated |
| TMC6 | 2.17686 | 6.47363 | 1.77E-42 | 1.03E-37 | Up-Regulated |
| MGAT4B | 1.265101 | 7.388494 | 2.07E-42 | 1.20E-37 | Up-Regulated |
| CD44 | 1.368109 | 8.972548 | 2.85E-42 | 1.66E-37 | Up-Regulated |
| SH2D4A | 1.247311 | 5.800459 | 3.11E-42 | 1.80E-37 | Up-Regulated |
| E2F1 | 2.083146 | 3.213491 | 3.38E-42 | 1.96E-37 | Up-Regulated |
| TMPRSS4 | 6.324149 | 6.041356 | 3.47E-42 | 2.01E-37 | Up-Regulated |
| PLEKHG5 | 1.242507 | 4.155233 | 3.59E-42 | 2.08E-37 | Up-Regulated |
| SLIT1 | 5.786112 | 6.288947 | 3.59E-42 | 2.08E-37 | Up-Regulated |
| GPRC5B | 1.389592 | 6.543167 | 3.67E-42 | 2.13E-37 | Up-Regulated |
| SYT12 | 6.645711 | 6.300486 | 4.13E-42 | 2.40E-37 | Up-Regulated |
| FOLH1 | 2.444255 | 1.583458 | 4.80E-42 | 2.78E-37 | Up-Regulated |
| CSF2 | 5.615484 | 0.266827 | 4.93E-42 | 2.86E-37 | Up-Regulated |
| OIT3 | 3.417459 | 1.213426 | 5.58E-42 | 3.24E-37 | Up-Regulated |
| FRMD3 | 2.660509 | 7.285027 | 6.65E-42 | 3.86E-37 | Up-Regulated |
| C5AR2 | 2.086396 | 1.663584 | 7.83E-42 | 4.54E-37 | Up-Regulated |
| TMEM79 | 2.158963 | 4.160824 | 8.51E-42 | 4.93E-37 | Up-Regulated |
| AC046143.3 | 1.741262 | -0.14068 | 8.62E-42 | 5.00E-37 | Up-Regulated |
| CTD-3035K23.7 | 3.69341 | 0.841993 | 8.67E-42 | 5.03E-37 | Up-Regulated |
| GLRB | 2.134923 | 2.455209 | 1.05E-41 | 6.07E-37 | Up-Regulated |
| RP11-98D18.9 | 1.448391 | 0.168822 | 1.06E-41 | 6.12E-37 | Up-Regulated |
| SREBF1 | 1.431653 | 7.083541 | 1.08E-41 | 6.27E-37 | Up-Regulated |
| EDIL3 | 2.132274 | 4.116936 | 1.44E-41 | 8.36E-37 | Up-Regulated |
| HAGLROS | 3.907959 | -0.53729 | 1.63E-41 | 9.44E-37 | Up-Regulated |
| AC007255.8 | 4.959019 | 2.346464 | 1.78E-41 | 1.03E-36 | Up-Regulated |
| RAB34 | 1.212602 | 7.177507 | 2.06E-41 | 1.19E-36 | Up-Regulated |
| CTD-2619J13.13 | 3.89913 | 1.242005 | 2.28E-41 | 1.32E-36 | Up-Regulated |
| PLXNA3 | 1.167318 | 6.010827 | 2.43E-41 | 1.41E-36 | Up-Regulated |
| TMEM132D | 4.000239 | 2.25112 | 2.45E-41 | 1.42E-36 | Up-Regulated |
| EDA2R | 1.466177 | 3.890248 | 3.43E-41 | 1.99E-36 | Up-Regulated |
| AC008063.2 | 4.542884 | -0.96899 | 3.80E-41 | 2.20E-36 | Up-Regulated |
| TBC1D2 | 2.067254 | 6.400174 | 3.83E-41 | 2.22E-36 | Up-Regulated |
| RP11-547C5.1 | 5.013329 | -1.09321 | 3.98E-41 | 2.30E-36 | Up-Regulated |
| MROH6 | 2.701723 | 3.497092 | 4.34E-41 | 2.51E-36 | Up-Regulated |
| SIPA1L2 | 1.494363 | 5.8632 | 4.38E-41 | 2.54E-36 | Up-Regulated |
| CLDN1 | 3.233678 | 8.593799 | 4.57E-41 | 2.65E-36 | Up-Regulated |
| GPRIN1 | 2.398447 | 1.34981 | 5.18E-41 | 3.00E-36 | Up-Regulated |
| PPP1R1B | 4.916226 | 3.478071 | 5.61E-41 | 3.25E-36 | Up-Regulated |
| KLK10 | 6.260137 | 6.43625 | 6.08E-41 | 3.53E-36 | Up-Regulated |
| TAX1BP3 | 1.222026 | 4.831057 | 6.23E-41 | 3.61E-36 | Up-Regulated |
| XKRX | 3.876827 | 3.182925 | 6.72E-41 | 3.89E-36 | Up-Regulated |
| DOK4 | 1.013396 | 4.971921 | 6.81E-41 | 3.95E-36 | Up-Regulated |
| RP3-483K16.4 | -3.07336 | -0.94475 | 8.36E-129 | 4.88E-124 | Down-Regulated |
| CTD-3014M21.1 | -2.47886 | -0.06493 | 1.54E-122 | 8.97E-118 | Down-Regulated |
| RNU12 | -3.79834 | -3.43281 | 1.05E-118 | 6.16E-114 | Down-Regulated |
| RPS6KA5 | -1.66139 | 2.696702 | 6.38E-113 | 3.72E-108 | Down-Regulated |
| LYVE1 | -2.95359 | 2.579437 | 1.33E-100 | 7.76E-96 | Down-Regulated |
| RNU1-1 | -3.27342 | -4.41349 | 6.09E-97 | 3.56E-92 | Down-Regulated |
| CDHR3 | -2.07689 | 1.377602 | 6.13E-97 | 3.58E-92 | Down-Regulated |
| RNU11 | -3.69421 | -4.13118 | 1.52E-93 | 8.85E-89 | Down-Regulated |
| MMRN1 | -3.09859 | 3.219296 | 9.16E-92 | 5.35E-87 | Down-Regulated |
| PLA2R1 | -2.74517 | 5.462181 | 3.44E-82 | 2.01E-77 | Down-Regulated |
| UGT2B11 | -3.37147 | -1.88911 | 1.95E-80 | 1.14E-75 | Down-Regulated |
| RASSF9 | -2.40794 | 1.846634 | 4.16E-80 | 2.43E-75 | Down-Regulated |
| RNU5F-1 | -3.7808 | -3.22992 | 1.42E-79 | 8.30E-75 | Down-Regulated |
| ABCA9 | -2.75032 | 1.171579 | 1.08E-78 | 6.30E-74 | Down-Regulated |
| RNVU1-6 | -4.124 | -3.51573 | 2.74E-71 | 1.60E-66 | Down-Regulated |
| PBX4 | -2.10471 | 2.715343 | 9.53E-71 | 5.56E-66 | Down-Regulated |
| SRF | -1.40398 | 6.141176 | 3.61E-69 | 2.10E-64 | Down-Regulated |
| KCNIP4 | -1.98524 | 1.745325 | 1.35E-68 | 7.87E-64 | Down-Regulated |
| CFD | -2.24154 | 3.858481 | 1.71E-68 | 9.95E-64 | Down-Regulated |
| PAPSS2 | -2.28924 | 3.988783 | 2.89E-68 | 1.69E-63 | Down-Regulated |
| LMOD1 | -2.28692 | 5.206819 | 3.50E-68 | 2.04E-63 | Down-Regulated |
| MLF1 | -1.26179 | 3.615979 | 2.41E-67 | 1.41E-62 | Down-Regulated |
| TPPP | -2.09192 | 3.983999 | 2.92E-67 | 1.70E-62 | Down-Regulated |
| GPC3 | -3.06637 | 1.862801 | 5.41E-67 | 3.16E-62 | Down-Regulated |
| IGSF10 | -2.98895 | 0.709344 | 1.30E-66 | 7.56E-62 | Down-Regulated |
| CTD-2194A8.2 | -3.4957 | -4.38451 | 1.60E-66 | 9.34E-62 | Down-Regulated |
| RELN | -3.38178 | 1.435568 | 1.24E-65 | 7.24E-61 | Down-Regulated |
| PLSCR4 | -1.40475 | 4.628701 | 1.58E-65 | 9.20E-61 | Down-Regulated |
| C8orf88 | -2.18747 | 0.27054 | 2.54E-65 | 1.48E-60 | Down-Regulated |
| PID1 | -2.39971 | 1.300405 | 8.37E-64 | 4.88E-59 | Down-Regulated |
| RP11-293M10.2 | -3.20757 | -3.69069 | 9.59E-64 | 5.60E-59 | Down-Regulated |
| HDAC4 | -1.15217 | 4.017596 | 3.80E-63 | 2.22E-58 | Down-Regulated |
| TBC1D4 | -1.699 | 5.412546 | 2.53E-62 | 1.48E-57 | Down-Regulated |
| MAFB | -1.68698 | 5.940755 | 5.44E-61 | 3.17E-56 | Down-Regulated |
| ADD3-AS1 | -1.4796 | -1.04838 | 1.06E-60 | 6.18E-56 | Down-Regulated |
| RP11-96J19.1 | -4.21627 | -4.36122 | 1.61E-60 | 9.36E-56 | Down-Regulated |
| EPHA3 | -2.01706 | 3.657981 | 5.26E-60 | 3.07E-55 | Down-Regulated |
| SNX18P3 | -2.04454 | -0.9261 | 3.71E-59 | 2.17E-54 | Down-Regulated |
| CH17-373J23.1 | -1.90331 | -2.06311 | 6.51E-59 | 3.80E-54 | Down-Regulated |
| AKR1E2 | -2.16879 | 0.258068 | 1.08E-58 | 6.29E-54 | Down-Regulated |
| MYH15 | -2.64602 | -1.51931 | 1.30E-58 | 7.61E-54 | Down-Regulated |
| RP11-545A16.3 | -2.53482 | -2.2195 | 1.61E-58 | 9.36E-54 | Down-Regulated |
| F10 | -2.18323 | 0.686429 | 2.42E-58 | 1.41E-53 | Down-Regulated |
| GRIA1 | -3.41195 | -3.29791 | 7.74E-57 | 4.51E-52 | Down-Regulated |
| LAYN | -1.75891 | 3.464434 | 1.47E-56 | 8.57E-52 | Down-Regulated |
| AC152010.1 | -8.83862 | -2.75664 | 5.51E-56 | 3.21E-51 | Down-Regulated |
| AADACP1 | -3.07572 | -0.54577 | 6.71E-56 | 3.91E-51 | Down-Regulated |
| GPM6A | -2.5651 | 2.758632 | 7.87E-56 | 4.59E-51 | Down-Regulated |
| IPCEF1 | -2.86488 | 4.956889 | 8.05E-56 | 4.69E-51 | Down-Regulated |
| DNAJB4 | -1.23184 | 5.273088 | 1.17E-55 | 6.81E-51 | Down-Regulated |
| AC093609.1 | -2.45293 | -2.2987 | 1.35E-55 | 7.85E-51 | Down-Regulated |
| BMP2 | -1.94023 | 5.099294 | 1.79E-55 | 1.04E-50 | Down-Regulated |
| CSGALNACT1 | -1.79399 | 6.26 | 3.67E-55 | 2.14E-50 | Down-Regulated |
| CTH | -1.51919 | 2.192534 | 5.01E-55 | 2.92E-50 | Down-Regulated |
| ATP2C2 | -2.47366 | 2.805714 | 5.22E-55 | 3.04E-50 | Down-Regulated |
| RNU2-63P | -3.54415 | -4.24116 | 7.61E-55 | 4.43E-50 | Down-Regulated |
| GPR125 | -1.36421 | 5.37172 | 2.80E-54 | 1.63E-49 | Down-Regulated |
| MATN2 | -2.15062 | 7.664605 | 3.45E-54 | 2.01E-49 | Down-Regulated |
| RYR2 | -2.8771 | 3.408106 | 4.74E-54 | 2.76E-49 | Down-Regulated |
| RNU5A-1 | -2.91374 | -3.90579 | 6.50E-54 | 3.79E-49 | Down-Regulated |
| RP11-160E2.6 | -2.91346 | -3.43257 | 8.95E-54 | 5.21E-49 | Down-Regulated |
| RNU6-2 | -2.808 | -4.01237 | 2.38E-53 | 1.39E-48 | Down-Regulated |
| RPS23P1 | -2.21228 | -3.08555 | 4.97E-53 | 2.89E-48 | Down-Regulated |
| ESRRAP2 | -2.04609 | -2.27171 | 5.54E-53 | 3.23E-48 | Down-Regulated |
| DDX25 | -2.10886 | 1.053319 | 7.04E-53 | 4.10E-48 | Down-Regulated |
| LRIG1 | -1.3264 | 5.422739 | 1.13E-52 | 6.58E-48 | Down-Regulated |
| KCNA1 | -4.06214 | -0.44403 | 1.14E-52 | 6.61E-48 | Down-Regulated |
| SNORD3B-1 | -3.40875 | -3.54179 | 2.43E-52 | 1.42E-47 | Down-Regulated |
| ACKR2 | -1.68412 | 0.188568 | 8.60E-52 | 5.01E-47 | Down-Regulated |
| RNVU1-15 | -2.12969 | -3.24436 | 1.30E-51 | 7.60E-47 | Down-Regulated |
| ANKS1B | -1.78288 | 2.083807 | 4.49E-51 | 2.61E-46 | Down-Regulated |
| AGPAT4 | -1.63934 | 2.479073 | 1.17E-50 | 6.81E-46 | Down-Regulated |
| TNS3 | -1.28047 | 6.658526 | 1.18E-50 | 6.87E-46 | Down-Regulated |
| ST7-AS1 | -1.38882 | 1.592124 | 1.74E-50 | 1.01E-45 | Down-Regulated |
| KLHL4 | -2.20318 | -0.30547 | 7.38E-50 | 4.30E-45 | Down-Regulated |
| AC105053.3 | -2.07853 | -1.6041 | 1.41E-49 | 8.20E-45 | Down-Regulated |
| PROX1 | -1.97505 | 3.454965 | 4.37E-49 | 2.54E-44 | Down-Regulated |
| GHR | -1.7045 | 2.04948 | 7.89E-49 | 4.59E-44 | Down-Regulated |
| AGR3 | -3.20555 | 0.058643 | 1.71E-48 | 9.93E-44 | Down-Regulated |
| CCDC173 | -1.65723 | 0.367925 | 2.02E-48 | 1.18E-43 | Down-Regulated |
| TFCP2L1 | -2.72086 | 5.69548 | 2.68E-48 | 1.56E-43 | Down-Regulated |
| KB-1732A1.1 | -1.53536 | -1.10584 | 3.48E-48 | 2.02E-43 | Down-Regulated |
| AC008753.6 | -2.16879 | -2.21394 | 3.63E-48 | 2.11E-43 | Down-Regulated |
| DCDC1 | -1.66796 | 0.247119 | 4.62E-48 | 2.69E-43 | Down-Regulated |
| ZFPM2 | -1.91128 | 2.974561 | 1.17E-47 | 6.80E-43 | Down-Regulated |
| PRKCQ | -1.14409 | 4.489206 | 1.35E-47 | 7.85E-43 | Down-Regulated |
| AC078899.3 | -2.87392 | -0.77706 | 1.81E-47 | 1.06E-42 | Down-Regulated |
| DNAH7 | -1.33879 | 2.495273 | 2.00E-47 | 1.16E-42 | Down-Regulated |
| KCTD16 | -2.84394 | 0.862437 | 2.56E-47 | 1.49E-42 | Down-Regulated |
| EYA1 | -2.60466 | -1.45998 | 3.24E-47 | 1.88E-42 | Down-Regulated |
| LINC00936 | -1.2456 | 1.373275 | 4.69E-47 | 2.73E-42 | Down-Regulated |
| RP4-568B10.1 | -2.89039 | -3.02079 | 6.37E-47 | 3.70E-42 | Down-Regulated |
| SLC25A33 | -1.35323 | 2.713022 | 9.94E-47 | 5.78E-42 | Down-Regulated |
| UBE2FP1 | -1.48631 | -1.98101 | 1.31E-46 | 7.60E-42 | Down-Regulated |
| RPS6KA6 | -1.42731 | 3.868874 | 1.42E-46 | 8.27E-42 | Down-Regulated |
| LPP-AS2 | -1.24322 | 0.323022 | 1.87E-46 | 1.09E-41 | Down-Regulated |
| ADH1B | -3.21456 | 2.429148 | 3.00E-46 | 1.74E-41 | Down-Regulated |
| ABCA10 | -1.61957 | 0.536753 | 4.03E-46 | 2.34E-41 | Down-Regulated |
| SLC6A15 | -3.85297 | -1.92094 | 4.48E-46 | 2.61E-41 | Down-Regulated |
| DLG2 | -2.34881 | 3.760853 | 5.16E-46 | 3.00E-41 | Down-Regulated |
| RP11-362F19.1 | -2.11899 | -0.0522 | 5.57E-46 | 3.24E-41 | Down-Regulated |
| TLE4 | -1.50183 | 3.579046 | 5.68E-46 | 3.30E-41 | Down-Regulated |
| RP1-140C12.2 | -2.71118 | -3.05773 | 5.87E-46 | 3.41E-41 | Down-Regulated |
| RP11-327P2.5 | -1.12815 | 1.684617 | 6.11E-46 | 3.55E-41 | Down-Regulated |
| AC007365.3 | -1.7307 | -2.12246 | 7.58E-46 | 4.40E-41 | Down-Regulated |
| CCDC81 | -1.16346 | -0.21104 | 9.08E-46 | 5.27E-41 | Down-Regulated |
| RNVU1-20 | -2.42116 | -3.39764 | 1.43E-45 | 8.32E-41 | Down-Regulated |
| RP11-322E11.5 | -2.10723 | -1.96315 | 1.46E-45 | 8.47E-41 | Down-Regulated |
| CCDC146 | -1.58079 | 3.668997 | 1.47E-45 | 8.56E-41 | Down-Regulated |
| CYTH3 | -1.00956 | 5.653491 | 1.87E-45 | 1.09E-40 | Down-Regulated |
| DPT | -3.24331 | 4.027996 | 2.28E-45 | 1.32E-40 | Down-Regulated |
| SEPT7-AS1 | -1.21478 | 1.118536 | 3.67E-45 | 2.13E-40 | Down-Regulated |
| ADH1C | -2.63046 | -1.95211 | 3.84E-45 | 2.23E-40 | Down-Regulated |
| RP11-317B3.2 | -3.07352 | -3.46255 | 4.42E-45 | 2.57E-40 | Down-Regulated |
| C11orf74 | -1.61741 | 4.099656 | 1.29E-44 | 7.48E-40 | Down-Regulated |
| CLUL1 | -1.78578 | 1.822652 | 1.53E-44 | 8.87E-40 | Down-Regulated |
| CXorf57 | -1.15528 | 1.906877 | 2.32E-44 | 1.35E-39 | Down-Regulated |
| RP3-442L6.4 | -3.25463 | -4.6315 | 2.57E-44 | 1.49E-39 | Down-Regulated |
| TLE1 | -1.17379 | 4.363039 | 2.73E-44 | 1.58E-39 | Down-Regulated |
| CLMN | -1.58467 | 3.698323 | 2.97E-44 | 1.72E-39 | Down-Regulated |
| OR2V1 | -4.18663 | -3.92102 | 3.21E-44 | 1.87E-39 | Down-Regulated |
| CTB-109A12.1 | -2.36621 | -2.89301 | 4.07E-44 | 2.36E-39 | Down-Regulated |
| TCEAL5 | -2.92905 | -0.19706 | 1.72E-43 | 9.98E-39 | Down-Regulated |
| TFPI | -1.40359 | 3.538727 | 1.85E-43 | 1.08E-38 | Down-Regulated |
| RP11-375I20.6 | -1.97016 | -1.18587 | 2.14E-43 | 1.24E-38 | Down-Regulated |
| RNA5SP470 | -2.76382 | -4.68405 | 3.45E-43 | 2.00E-38 | Down-Regulated |
| TMEM232 | -1.19278 | 1.502574 | 4.68E-43 | 2.71E-38 | Down-Regulated |
| SLC17A7 | -1.83595 | -0.28008 | 4.98E-43 | 2.89E-38 | Down-Regulated |
| MIR100HG | -1.7223 | -0.94324 | 5.57E-43 | 3.23E-38 | Down-Regulated |
| TSPAN19 | -3.11858 | -2.4826 | 6.88E-43 | 3.99E-38 | Down-Regulated |
| CNTN5 | -2.63633 | 1.376653 | 1.10E-42 | 6.39E-38 | Down-Regulated |
| AL035610.1 | -2.95189 | -3.93467 | 1.18E-42 | 6.87E-38 | Down-Regulated |
| LIMD1-AS1 | -1.39841 | -2.29433 | 2.31E-42 | 1.34E-37 | Down-Regulated |
| ANKRD37 | -1.34201 | 2.405914 | 2.36E-42 | 1.37E-37 | Down-Regulated |
| RP11-528A10.1 | -2.10274 | -3.71898 | 2.58E-42 | 1.50E-37 | Down-Regulated |
| EPHB1 | -1.86798 | 3.451447 | 4.23E-42 | 2.45E-37 | Down-Regulated |
| LINC01354 | -2.21304 | -0.79265 | 5.77E-42 | 3.34E-37 | Down-Regulated |
| ANK2 | -2.17659 | 4.106986 | 5.98E-42 | 3.47E-37 | Down-Regulated |
| RP11-305O6.4 | -1.57215 | -2.23771 | 6.84E-42 | 3.97E-37 | Down-Regulated |
| STARD13 | -1.26179 | 5.19802 | 6.86E-42 | 3.98E-37 | Down-Regulated |
| PIK3C2G | -3.06717 | -4.16916 | 7.28E-42 | 4.22E-37 | Down-Regulated |
| SOX5 | -1.57114 | 1.003203 | 7.45E-42 | 4.32E-37 | Down-Regulated |
| IGSF9B | -1.41034 | 1.619432 | 9.13E-42 | 5.29E-37 | Down-Regulated |
| STARD13-AS | -1.54045 | -1.8993 | 9.25E-42 | 5.36E-37 | Down-Regulated |
| RP11-704M14.1 | -3.04744 | -4.66763 | 1.05E-41 | 6.08E-37 | Down-Regulated |
| RP11-118F19.1 | -2.37472 | -2.90145 | 1.05E-41 | 6.10E-37 | Down-Regulated |
| AC009237.16 | -2.52599 | -3.18964 | 1.23E-41 | 7.12E-37 | Down-Regulated |
| RP4-710M3.2 | -3.1606 | -3.77043 | 1.58E-41 | 9.15E-37 | Down-Regulated |
| RNU4ATAC9P | -3.16677 | -4.81457 | 1.75E-41 | 1.02E-36 | Down-Regulated |
| C9orf47 | -1.42259 | -1.40421 | 1.86E-41 | 1.08E-36 | Down-Regulated |
| SYNE1 | -1.61787 | 6.769763 | 1.94E-41 | 1.12E-36 | Down-Regulated |
| TMEM171 | -2.01919 | 3.470057 | 2.25E-41 | 1.30E-36 | Down-Regulated |
| PLCH1 | -2.10531 | 2.970244 | 2.83E-41 | 1.64E-36 | Down-Regulated |
| RP11-12J10.3 | -1.34947 | -0.48171 | 3.77E-41 | 2.18E-36 | Down-Regulated |
| FTCDNL1 | -1.43399 | 1.901947 | 3.99E-41 | 2.32E-36 | Down-Regulated |
| RP11-806L2.2 | -1.78238 | -1.9431 | 4.66E-41 | 2.70E-36 | Down-Regulated |
| RP13-270P17.2 | -1.08494 | -0.14941 | 5.22E-41 | 3.02E-36 | Down-Regulated |
| SLC26A7 | -2.3781 | 8.105579 | 5.45E-41 | 3.16E-36 | Down-Regulated |
| EPHA6 | -1.81919 | -0.53222 | 5.97E-41 | 3.46E-36 | Down-Regulated |
| RNF150 | -1.92738 | 3.758699 | 7.38E-41 | 4.28E-36 | Down-Regulated |
| CITED2 | -1.54409 | 7.655912 | 7.97E-41 | 4.62E-36 | Down-Regulated |
| TMEM253 | -1.84283 | -1.22719 | 8.59E-41 | 4.98E-36 | Down-Regulated |
| SH3RF1 | -1.03149 | 5.224479 | 9.32E-41 | 5.40E-36 | Down-Regulated |
| LONRF3 | -1.54253 | 3.345617 | 1.09E-40 | 6.31E-36 | Down-Regulated |
| RP11-70C1.1 | -1.69921 | -2.47964 | 1.18E-40 | 6.83E-36 | Down-Regulated |
| SCML2 | -1.32448 | 0.925768 | 1.19E-40 | 6.90E-36 | Down-Regulated |
| RP11-69I8.3 | -2.5005 | -2.45969 | 1.44E-40 | 8.33E-36 | Down-Regulated |
| PRKX | -1.27954 | 6.213301 | 1.52E-40 | 8.79E-36 | Down-Regulated |
| RNU5D-1 | -3.02012 | -4.17826 | 2.41E-40 | 1.40E-35 | Down-Regulated |
| CTD-2008L17.2 | -2.65014 | -0.4117 | 3.89E-40 | 2.25E-35 | Down-Regulated |
| AOX1 | -2.67293 | 2.834203 | 4.30E-40 | 2.49E-35 | Down-Regulated |
| C6orf118 | -2.60874 | -2.89136 | 4.95E-40 | 2.86E-35 | Down-Regulated |
| LRRIQ1 | -1.41976 | 1.079325 | 6.15E-40 | 3.56E-35 | Down-Regulated |
| RP11-120D5.1 | -1.34518 | -2.13853 | 8.28E-40 | 4.80E-35 | Down-Regulated |
| FHDC1 | -1.34362 | 5.394364 | 8.43E-40 | 4.88E-35 | Down-Regulated |
| RP11-253E3.3 | -1.43192 | 1.615076 | 8.75E-40 | 5.06E-35 | Down-Regulated |
| KB-1448A5.1 | -2.60576 | -3.54598 | 9.10E-40 | 5.27E-35 | Down-Regulated |
| RP11-1D12.2 | -1.82956 | -3.11074 | 9.45E-40 | 5.47E-35 | Down-Regulated |
| FAM46B | -1.75389 | 2.735411 | 9.48E-40 | 5.49E-35 | Down-Regulated |
| NEK10 | -1.39273 | -1.14278 | 9.81E-40 | 5.68E-35 | Down-Regulated |
| RP11-521B24.3 | -1.01978 | 0.788973 | 1.71E-39 | 9.92E-35 | Down-Regulated |
| PDLIM3 | -1.90603 | 4.248308 | 2.30E-39 | 1.33E-34 | Down-Regulated |
| WWOX | -1.01516 | 4.371946 | 2.37E-39 | 1.37E-34 | Down-Regulated |
| CAMK1D | -1.25124 | 5.275581 | 3.02E-39 | 1.75E-34 | Down-Regulated |
| AF186192.5 | -1.12986 | 1.227183 | 3.41E-39 | 1.97E-34 | Down-Regulated |
| GARNL3 | -1.13377 | 2.720788 | 3.58E-39 | 2.07E-34 | Down-Regulated |
| MIR4697HG | -1.44944 | 3.087273 | 3.85E-39 | 2.23E-34 | Down-Regulated |
| ZFP36L2 | -1.02945 | 8.287585 | 4.53E-39 | 2.62E-34 | Down-Regulated |
| GLI1 | -2.18021 | -0.59238 | 5.47E-39 | 3.17E-34 | Down-Regulated |
| RP11-455O6.5 | -1.84885 | -0.24485 | 6.78E-39 | 3.92E-34 | Down-Regulated |
| FBXO30 | -1.00686 | 4.616124 | 7.13E-39 | 4.13E-34 | Down-Regulated |
| SLC25A25 | -1.37653 | 4.892606 | 7.35E-39 | 4.25E-34 | Down-Regulated |
| LRRN4CL | -2.62875 | -0.28842 | 1.00E-38 | 5.79E-34 | Down-Regulated |
| ARHGAP6 | -1.10927 | 5.221544 | 1.17E-38 | 6.78E-34 | Down-Regulated |
| SMOC2 | -2.22415 | 5.620518 | 1.41E-38 | 8.13E-34 | Down-Regulated |
| ACACB | -1.30005 | 5.070125 | 1.41E-38 | 8.18E-34 | Down-Regulated |
| CYP7B1 | -1.45227 | 2.022873 | 1.58E-38 | 9.13E-34 | Down-Regulated |
| PRTG | -1.51205 | 4.405362 | 2.78E-38 | 1.61E-33 | Down-Regulated |
| WDR72 | -1.87582 | 5.83046 | 2.96E-38 | 1.71E-33 | Down-Regulated |
| TNFRSF11B | -2.01257 | 5.667129 | 3.37E-38 | 1.95E-33 | Down-Regulated |
| LYPLAL1-AS1 | -1.35444 | -2.32153 | 4.16E-38 | 2.41E-33 | Down-Regulated |
| FAM180B | -3.09673 | -0.2413 | 4.18E-38 | 2.42E-33 | Down-Regulated |
| RP11-162G10.5 | -1.49813 | -1.69598 | 4.75E-38 | 2.75E-33 | Down-Regulated |
| EGR2 | -2.16041 | 6.556838 | 5.03E-38 | 2.91E-33 | Down-Regulated |
| JUN | -1.65222 | 8.980911 | 5.65E-38 | 3.26E-33 | Down-Regulated |
| DNAJB1 | -1.18609 | 6.878022 | 6.62E-38 | 3.83E-33 | Down-Regulated |
| AIF1L | -1.35691 | 7.166629 | 8.91E-38 | 5.15E-33 | Down-Regulated |
| CTC-400I9.1 | -3.87117 | -4.0839 | 1.03E-37 | 5.94E-33 | Down-Regulated |
| DNAH6 | -1.27477 | 2.246746 | 1.37E-37 | 7.91E-33 | Down-Regulated |
| CHRDL1 | -2.90812 | 3.643028 | 1.57E-37 | 9.06E-33 | Down-Regulated |
| RP11-242F24.1 | -2.48274 | -2.71426 | 2.83E-37 | 1.64E-32 | Down-Regulated |
| HBB | -2.18227 | 5.46311 | 3.03E-37 | 1.75E-32 | Down-Regulated |
| RP3-340B19.3 | -1.35188 | 1.292949 | 3.29E-37 | 1.90E-32 | Down-Regulated |
| SLC1A7 | -2.26519 | -1.24194 | 3.37E-37 | 1.95E-32 | Down-Regulated |
| C1QTNF7 | -2.01004 | 1.167684 | 3.73E-37 | 2.15E-32 | Down-Regulated |
| TTC30A | -1.10101 | 3.567167 | 5.45E-37 | 3.15E-32 | Down-Regulated |
| NMNAT3 | -1.12436 | 2.386237 | 5.81E-37 | 3.35E-32 | Down-Regulated |
| ENO4 | -1.20966 | -0.68477 | 5.82E-37 | 3.36E-32 | Down-Regulated |
| BCL2 | -1.39837 | 6.224847 | 6.00E-37 | 3.47E-32 | Down-Regulated |
| AC010226.4 | -1.15864 | -0.30646 | 6.17E-37 | 3.56E-32 | Down-Regulated |
| ANGPTL1 | -1.71302 | 4.491573 | 6.85E-37 | 3.96E-32 | Down-Regulated |
| SCG3 | -2.02403 | -1.19023 | 7.16E-37 | 4.14E-32 | Down-Regulated |
| CFAP61 | -1.44335 | -1.01295 | 7.18E-37 | 4.15E-32 | Down-Regulated |
| AC007563.5 | -1.74554 | -3.63923 | 9.98E-37 | 5.77E-32 | Down-Regulated |
| LINC01539 | -2.49629 | -0.80227 | 9.99E-37 | 5.77E-32 | Down-Regulated |
| ART5 | -1.96732 | -0.31344 | 1.05E-36 | 6.06E-32 | Down-Regulated |
| RP11-779O18.1 | -2.18727 | -3.5158 | 1.12E-36 | 6.49E-32 | Down-Regulated |
| RNU1-2 | -2.8851 | -4.6584 | 1.13E-36 | 6.51E-32 | Down-Regulated |
| DGKI | -2.32505 | 5.06129 | 1.27E-36 | 7.31E-32 | Down-Regulated |
| C4orf22 | -1.97262 | -2.48028 | 1.40E-36 | 8.07E-32 | Down-Regulated |
| RP11-503E24.2 | -1.51378 | -1.23951 | 1.92E-36 | 1.11E-31 | Down-Regulated |
| CFAP43 | -1.34232 | 2.025139 | 2.34E-36 | 1.35E-31 | Down-Regulated |
| BX322557.10 | -1.09229 | 1.450689 | 2.69E-36 | 1.55E-31 | Down-Regulated |
| RP11-456O19.2 | -2.7395 | -3.94878 | 2.96E-36 | 1.71E-31 | Down-Regulated |
| SEC14L3 | -3.24966 | -1.50479 | 4.05E-36 | 2.34E-31 | Down-Regulated |
| SYT9 | -2.0816 | 0.156347 | 5.19E-36 | 2.99E-31 | Down-Regulated |
| LIFR | -1.84718 | 5.641829 | 5.44E-36 | 3.14E-31 | Down-Regulated |
| AC012360.6 | -2.21463 | -2.39454 | 6.17E-36 | 3.56E-31 | Down-Regulated |
| RP11-444D3.1 | -1.82948 | -2.99451 | 6.38E-36 | 3.68E-31 | Down-Regulated |
| ID4 | -1.23656 | 8.161934 | 6.50E-36 | 3.75E-31 | Down-Regulated |
| RP11-168K11.3 | -2.3113 | -3.78013 | 6.63E-36 | 3.82E-31 | Down-Regulated |
| USP49 | -1.07126 | 3.197564 | 6.70E-36 | 3.87E-31 | Down-Regulated |
| OSR1 | -2.18732 | 0.391729 | 6.81E-36 | 3.93E-31 | Down-Regulated |
| VTRNA2-1 | -2.59002 | -4.78228 | 6.97E-36 | 4.02E-31 | Down-Regulated |
| DBX2 | -2.78214 | -3.7085 | 9.09E-36 | 5.25E-31 | Down-Regulated |
| RP1-47A17.1 | -2.0743 | -3.43132 | 1.01E-35 | 5.85E-31 | Down-Regulated |
| SYP | -1.11719 | 0.927009 | 2.04E-35 | 1.18E-30 | Down-Regulated |
| NRXN1 | -3.01852 | -2.19694 | 2.09E-35 | 1.21E-30 | Down-Regulated |
| NUAK2 | -1.2182 | 4.798505 | 2.14E-35 | 1.24E-30 | Down-Regulated |
| AC006042.7 | -1.38448 | -2.74749 | 2.71E-35 | 1.56E-30 | Down-Regulated |

| **Table S2. The literatures curated EMT-related genes from dbEMT2** |
| --- |
| Genes |
| TGFB1, SNAI1, ZEB1, CDH1, TWIST1, CTNNB1, STAT3, SNAI2, HIF1A, AKT1, MIR200C, ZEB2, SMAD3, EGFR, MIR200B, SMAD2, MIR200A, NOTCH1, MIR21, ILK, GSK3B, NFKB1, IL6, HMGA2, CXCR4, TP53, EGF, MTDH, MAPK1, CD44, BMI1, PTEN, MTOR, VIM, RAC1, MMP9, MMP2, GLI1, MET, SMAD4, KLF4, WNT5A, MYC, SMAD7, SIRT1, CTGF, CCR7, PROM1, TGFB2, PIK3CA, MIR34A, RHOA, AR, CD274, FOXM1, YAP1, SRC, MIR30A, MIR205, MALAT1, JAK2, WWTR1, CDH2, TNF, CXCL12, RELA, MAPK3, MIR221, MIR145, CXCL8, HGF, FN1, TWIST2, TP63, NANOG, VEGFA, SP1, S100A4, PTK2, MAPK7, MUC1, HMGB1, EZH2, ESR1, HOTAIR, CAV1, SOX2, BMP7, BMP4, SHH, PTGS2, POU5F1, PRRX1, KRAS, ID1, H19, FGFR1, POSTN, FOXQ1, MTA1, GRHL2, TRPS1, ADAM17, T, AURKA, BMP2, CCL18, CCL2, ROCK1, MIR429, ESRP1, PPARG, NTRK2, EPCAM, MIR93, MIR204, MIR203A, MIR186, MIR155, MIR148A, LOXL2, LGALS1, JUN, ITGB1, HDAC1, GREM1, KDM1A, AGER, NUMB, WNT1, EZR, TLR4, TCF3, STK11, UCA1, CCL21, CCL20, LEF1, PAX2, DDR2, YBX1, MCAM, MIR26B, MIR23A, MIR214, MIR19A, MIR150, MIR141, MIR124-1, MIR101-1, LOX, LCN2, IL17A, IL1B, IGF1R, IGF1, HSF1, HNF4A, FOXA1, FOXC2, ERBB2, EGR1, KLF8, NES, LINC-ROR, MIR300, NUAK1, DCLK1, CLDN1, RUNX2, TET1, TP73, THBD, TGM2, TGFBR3, TGFBR2, TGFB3, STIM1, SRF, BRCA1, SPARC, SOX9, SOX4, FSCN1, SIX1, SDC1, SATB1, BCL2L1, OVOL2, MKL1, NDRG2, EIF5A2, PARD3, PRKCI, AXL, FBXW7, TRIM33, PAK1, OSM, ROR2, NOTCH2, MSX2, MMP14, MIR9-1, MIR30C2, MIR30C1, MIR29C, MIR29B1, MIR26A1, MIR137, MIR130B, MIR10B, MIR106B, MIR106A, MIRLET7A1, LEP, L1CAM, RHOC, CYR61, BIRC5, HSPA5, HOXA13, ANXA2, PDCD4, GATA3, ALK, FLOT2, FOXO3, FOXC1, FGFR2, FGF2, AKT2, EPAS1, DNMT1, DAB2, CUX1, CTNND1, CTBP1, MAPK14, NLRP3, CYTOR, AGR2, NDRG1, CDKN1B, CD24, TNFSF15, GDF15, TP53INP1, TM4SF5, WNT3A, WISP2, NRP1, ACTL6A, CUL4A, FZD7, FOSL1, ADAM12, PDGFD, VASH2, TBL1XR1, TNFAIP8L2, YWHAG, YY1, XBP1, WT1, VDR, CCR2, MIR655, NR2C2, TIMP2, TGFBR1, TGFA, TERT, MIR630, MIR590, TCF4, BSG, SPRR2A, SPP1, SKIL, SMURF2, SFRP2, CXCL5, CCL5, BDNF, BCL2, RB1, PTPN11, MIR491, MIR485, CEMIP, SALL4, TMPRSS4, MAPK8, PRKCA, MEG3, TUG1, SEMA4C, PIN1, PGF, SERPINF1, GOLM1, PAWR, MIR375, MIR361, NODAL, ATM, MUC4, MMP7, FOXO4, MAP3K4, MAP3K3, MDM2, MIR96, MIR31, MIR222, MIR218-1, MIR181A1, MIR194-1, MIR185, MIR182, MIR181A2, MIR17, MIR143, MIR130A, MIR128-1, MIR10A, JUNB, ITGA5, IGFBP7, HSPB1, HSPA4, HOXB7, FOXA2, HMOX1, HDGF, HAS2, SENP1, RGCC, NEAT1, GPC3, USP22, FOXO1, DKK1, EFEMP1, VWCE, F2R, ELF5, EPHA2, EDN1, JAG1, DLX2, CTSL, PARP1, CRKL, FERMT2, TXNIP, SPRY2, ADAM10, TRIM28, NAMPT, MIR1271, HDAC6, ZBTB33, CDC42, HS3ST3B1, SETDB1, HDAC4, UBE3C, BAG3, MAGED1, CD36, ABCG2, NTN1, ADIPOQ, RNF8, ACVR1, CCNA2, SQSTM1, SPHK1, HDAC3, WISP3, NRP2, RUNX3, STC2, RUNX1, TNFSF11, BHLHE40, LGR5, HAVCR2, AFAP1L2, DYRK2, PITPNM3, AXIN2, AXIN1, NCOA3, ACTN4, ESRP2, PEAK1, CA9, YWHAZ, KDM6A, TUFT1, TPBG, TP53BP2, TLE1, TIMP1, TIAM1, TGFB1I1, TEAD1, TCF21, MIR616, MIR612, MAP3K7, STAT5B, STAT5A, STAT1, BRAF, SRI, SPOCK1, SOX5, BMP6, SKP2, SIM2, SDC2, CCL19, CEACAM1, RPS6KB1, ALX4, RNH1, CCND1, KDM5A, ACTA2, RAF1, PTX3, CIP2A, MTA3, MIR506, MIR497, MIR494, MIR489, PTHLH, PBXIP1, PAK5, CTNNBIP1, KLK6, PRSS8, PROP1, MAP2K1, ERBIN, BTBD7, PRKAA1, TRIM62, RNF111, IL17RD, PLAUR, PKD1, CDK14, PFN2, SUFU, IL23A, MAGEC2, HOOK1, PDCD1, CRIM1, EGFL7, PCBP1, F11R, SERPINE1, FURIN, PEBP1, P2RY2, NFKBIA, NF1, NCL, CEACAM6, MIR196B, MIR373, MIR135B, ZFAS1, MMP3, MMP1, ASCL1, MGAT3, MDK, MCL1, MIR34C, MIR33A, MIR30D, MIR301A, MIR27A, MIR24-1, MIR224, MIR223, MIR218-2, MIR206, MIR20A, MIR199A1, MIR193A, MIR191, MIR187, MIR183, MIR16-1, MIR15B, MIR15A, MIR153-1, MIR146A, MIR135A1, MIR134, MIR132, MIR124-3, MIR124-2, MIR122, MIR101-2, MIR100, LMNA, LGALS3, LASP1, LAMA5, KIT, CD82, JARID2, ITGB4, ITGA3, ITGA2, AQP5, AQP3, IL11, CXCR2, MACC1, TNC, HES1, PRMT1, HRAS, HOXB9, HOXA10, HMGB3, HIC1, HDAC2, H2AFX, GSN, UHRF1, CPS1-IT1, HIPK2, GRN, GPR32, ANG, GLI2, GJB1, NOX1, LRIG1, SLC39A6, FZD2, FUT4, BRD4, SIRT3, DICER1, SATB2, BOP1, EPB41L3, KDM4B, FGF9, FGF1, ETV4, ESRRA, ESR2, ERG, EPS8, EPO, EPHB2, GKN2, TET3, AHR, E2F1, HBEGF, DLX4, DAB2IP, PAQR3, CTSZ, GLIPR2, SLC30A7, RBFOX3, CSNK2B, CRYAB, LINC00261, CRK, CREB1, CLDN7, CLDN4, CLU, UHRF2, EHMT2, PROKR1, WASF3, CFTR, KHDRBS1, TRIM16, CEBPB, CEBPA, HOXB13, TACC3, CDX2, FAM3C, CDKN2A, CDK5, NPTN-IT1, SPRY4-IT1, ZEB2-AS1, MIR1236, MIR675, FGF19, RBM8A, MRC2, GAB2, CD151, HDAC9, SEMA3E, VGLL4, CD63, MICAL2, ISG15, PITPNM1, CXCL14, ONECUT2, ROCK2, RASAL2, EIF2AK3, MAP4K4, QKI, HAND1, CYP7B1, MUC16, FOXP2, ZFYVE9, SLC9A3R1, KL, LHX2, IGSF8, NREP, CD14, NOG, LRRFIP1, MARVELD3, ARHGEF2, SMC3, LATS1, NMI, AOC4P, PSTPIP1, SOCS3, HS6ST2, CCNG2, CDKL2, BTRC, CCND2, TIMELESS, NR1I2, KAT2B, INPP4B, SCEL, TNFRSF11A, TNFSF10, TNFSF12, CBR1, EED, ABCC3, CBLB, EIF3I, IRS2, SCIN, IKBKG, PIK3R3, AJUBA, PPM1D, GEMIN2, USMG5, SEMA7A, LOXL3, HINT2, SPZ1, TEAD2, FOXN1, CUL3, KAT8, SPOP, MARVELD1, ITCH, YPEL3, CAPZA1, CAPNS1, SMC1A, USP9X, VANGL1, TRIM11, WNT5B, IFT88, SETD7, PTP4A2, MED28, ELL3, NUBPL, FBXO11, MICALL2, LIN28A, TRPM8, DDR1, ZYX, ZNF217, PCGF2, ZNF143, MIR802, MIR454, MZF1, ZFP36, WNT11, WNT6, VTN, VSNL1, VHL, VCP, UCP2, UCHL3, TYRO3, TYMS, TXN, TUFM, C5AR1, PHLDA2, TSC2, TSC1, MIR663A, TRPC5, ACTG2, TP53BP1, TNXB, TNS1, TLN1, TLE4, TJP1, TIMP3, NR2F2, TFCP2, TDGF1, MIR92B, MIR646, MIR639, MIR638, TCF7, MIR573, TBX3, TBX2, TAZ, KLF5, ST14, SREBF1, SOX3, SON, SOD2, MIR487B, STK33, SLIT3, SLC2A1, SKP1, SKI, ST8SIA1, ST6GAL1, SIAH2, SIAH1, SHC1, CPEB1, AKTIP, SFTPC, SOX17, SEMA4A, ELSPBP1, GOLPH3, CX3CL1, CCL25, CCL22, CCL3, SERPINB4, ATXN1, SALL1, S100P, S100A9, S100A8, S100A6, S100A2, ROS1, RORC, EXOC4, BCL6, RGS3, RDX, HAS2-AS1, RBP2, SENP2, NTN4, RASA1, PRUNE1, CXCL16, PTPRZ1, NKX3-2, PTPN14, PTPN6, SCUBE2, EPB41L5, PTN, STIM2, MTUS1, MKL2, MIR522, MIR517C, MIR520G, MIR452, RAB22A, CAMK1D, TBX20, POGLUT1, PROX1, AJAP1, PRKCQ, HDAC8, PAG1, KDM3A, FOXJ2, PRKCE, PRKAA2, FERMT1, MIR449A, LGR4, BRF2, IMP3, PTPA, PPP1R8, GIPC2, POMC, ERRFI1, MBD3, PLS3, PLAGL2, PLA2G4A, PIK3R1, SERPINI1, ABCB1, STK26, WWOX, UIMC1, CYB5R1, PDGFRB, ACKR4, PDGFB, LIMA1, PRRX2, PDE4A, UBR5, PLAC8, TFDP3, PCSK1, ING4, ANGPTL4, PCMT1, GMNN, PCDH9, PBX3, PARD6A, NEUROG3, IL22, PRDX1, DUXAP9, OPRM1, MIR424, MIR382, MIR381, NUCB2, ROR1, NTRK3, NPPA, NOV, NOTCH4, NOTCH3, NME1, NKX6-1, NFIL3, NFIC, MYL2, MYD88, MYCN, MYBL2, MYB, TRIM37, MUC2, MST1R, MSN, MIR345, MIR331, MIR326, MMP19, MMP13, MMP11, MMP8, MLLT3, ASCL2, MITF, CXCL9, MEF2D, MDM4, ART1, SMAD9, ARRB1, MIR7-1, MIR30E, MIR30B, MIR302A, MIR29A, MIR26A2, MIR25, MIR24-2, MIR23B, MIR217, MIR216A, MIR211, MIR208A, MIR199A2, MIR190A, MIR181B1, MIR153-2, MIR152, MIR149, MIR144, MIR136, MIR129-1, MIR125B1, MIRLET7G, MIRLET7D, MIRLET7B, LYN, LTBP1, LRP6, LMNB1, LIMS1, LIF, LGALS9, LAMC2, USP17L9P, RHOG, NANOGP8, KRT19, RHOB, KMT5A, KRT18, KRT17, KRT8, KRT7, KIF5B, KDR, KCNN4, KCNH1, JAK3, JAG2, ITGB6, ITGB3, IRS1, IRF6, AQP9, ITGA6, FOXK2, IL18, IL6R, IL4, FAS, IGFBP3, MCRIP1, IRGM, NANOS3, IDH2, IDH1, ID2, IRF8, RAB43, HTN1, HSP90AA1, HSPB2, BIRC2, HRG, HPGD, HOXD9, APBB1, HOXA9, HNRNPAB, NR4A1, HK2, HIP1, EHD2, ANXA5, H2AFZ, ANXA1, REPIN1, OLA1, CARD10, GSK3A, BRD7, TBK1, FHOD1, GRIN1, ANPEP, TUSC7, LAMA1, CAVIN1, GPI, GOLGA2, GLRX, GLS, EML4, GLO1, GLS2, AGO2, DKK3, BBC3, LYPD3, GJB2, AMHR2, GH1, GATA6, GATA1, GAPDH, SIN3A, LETMD1, BRMS1, ARMC8, TXN2, SPDEF, FSCN2, CBY1, CERS6, NR5A2, FSHR, IL27, ZMYND8, CCNDBP1, PADI4, MMD, SCRIB, SUZ12, NCSTN, ARHGEF12, NEDD4L, FMR1, EXOC7, FLT1, FLNA, KDM6B, PLXND1, PEG10, SNW1, ALDOB, CEP164, FKBP5, PHLDA1, XRN2, FHL2, FHL1, SCUBE3, FCN2, ZEB1-AS1, FBP1, FASN, FBLN1, ALDH1A1, F3, F2RL2, F2, ETV1, ETS2, ETS1, ESRRB, ALAD, ERN1, ERF, EPHB3, EPHA8, EPHA4, EP300, ENO1, ENG, EMP3, FBXO45, RPL22L1, ELK3, ELK1, CYP4Z1, ELAVL1, EIF5A, EIF4G1, EIF4E, EIF2S1, RMST, EFNB2, EEF1D, EDNRA, ECT2, DVL2, AGTR1, DNMT3B, DDX5, DAPK1, CYP3A5, PEBP4, ZNF746, CYP1B1, CMTM8, CSPG4, VCAN, CSK, CSF2, CRP, CRMP1, FOXR2, CREBBP, ADM, KLF6, COL8A2, COL8A1, KLF17, CNTN1, MSI2, CCR6, CCR5, CLK2, LRG1, CKS2, BATF2, CTHRC1, CIRBP, MGLL, VSIG4, EGLN3, PKP3, RASSF1, FSTL1, PTP4A3, BVES, FOXN3, PIM2, EHD1, PPARGC1A, PGRMC1, HPSE, CCR9, MTHFD2, KDM5B, GNA13, LEFTY1, PDPN, CXCL13, FBLN5, CEACAM5, FST, MAD2L2, TAB1, RACK1, PAK4, CDKN1A, SPRY1, MSLN, PSME3, LINC01186, CDK3, G3BP1, TRAP1, CDH13, CDH11, NR1H3, MICA, OCLN, DNAJB6, MIR1181, CDH5, FRAT1, PDCD6IP, HDAC5, MIR124-2HG, MIR875, MIR888. |

| **Table S3. 244 prognosis related DE-ERGs identified in PTC** | | | | | |
| --- | --- | --- | --- | --- | --- |
| Genes | logFC | logCPM | PValue | FDR | Regulated |
| PLXND1 | 1.65252 | 7.426605 | 3.28E-74 | 1.91E-69 | Up-Regulated |
| ITGA3 | 1.791318 | 9.597039 | 2.69E-38 | 1.56E-33 | Up-Regulated |
| CX3CL1 | 1.437633 | 6.714217 | 4.53E-37 | 2.62E-32 | Up-Regulated |
| ETV1 | 1.381506 | 5.532467 | 1.28E-26 | 7.26E-22 | Up-Regulated |
| MRC2 | 3.165641 | 7.578052 | 2.02E-46 | 1.17E-41 | Up-Regulated |
| PLAUR | 1.763035 | 4.776669 | 4.68E-14 | 2.52E-09 | Up-Regulated |
| EHD2 | 1.067737 | 7.007634 | 2.75E-32 | 1.58E-27 | Up-Regulated |
| FAS | 1.001413 | 4.949413 | 1.75E-15 | 9.50E-11 | Up-Regulated |
| CD44 | 1.368109 | 8.972548 | 2.85E-42 | 1.66E-37 | Up-Regulated |
| GAB2 | 1.005589 | 4.940212 | 1.27E-21 | 7.12E-17 | Up-Regulated |
| VCAN | 2.058848 | 5.498287 | 1.58E-12 | 8.38E-08 | Up-Regulated |
| TNC | 2.261396 | 7.0024 | 6.53E-16 | 3.56E-11 | Up-Regulated |
| ROS1 | 3.833897 | -0.63602 | 4.16E-08 | 0.002054 | Up-Regulated |
| HDAC9 | 1.334774 | 4.12038 | 6.15E-16 | 3.35E-11 | Up-Regulated |
| LTBP1 | 1.351898 | 4.944623 | 4.01E-11 | 2.09E-06 | Up-Regulated |
| LAMC2 | 1.403926 | 7.823523 | 1.26E-25 | 7.15E-21 | Up-Regulated |
| SREBF1 | 1.431653 | 7.083541 | 1.08E-41 | 6.27E-37 | Up-Regulated |
| TRPC5 | 5.228446 | 3.853798 | 3.60E-26 | 2.05E-21 | Up-Regulated |
| TP63 | 2.582745 | 2.221388 | 1.92E-15 | 1.04E-10 | Up-Regulated |
| NOTCH3 | 1.262457 | 6.864524 | 5.83E-34 | 3.36E-29 | Up-Regulated |
| MCAM | 1.34696 | 6.564255 | 4.85E-30 | 2.78E-25 | Up-Regulated |
| CEACAM6 | 5.001633 | 3.786276 | 1.35E-22 | 7.59E-18 | Up-Regulated |
| TYRO3 | 2.068194 | 3.793499 | 9.59E-50 | 5.58E-45 | Up-Regulated |
| TGFB2 | 1.177497 | 3.889792 | 1.74E-09 | 8.82E-05 | Up-Regulated |
| IL11 | 1.687031 | -2.09067 | 3.20E-09 | 0.000161 | Up-Regulated |
| MMP11 | 3.613042 | 3.824626 | 1.05E-30 | 6.03E-26 | Up-Regulated |
| LGALS1 | 1.711484 | 7.459515 | 6.99E-22 | 3.92E-17 | Up-Regulated |
| HMOX1 | 1.065461 | 4.998621 | 1.26E-09 | 6.43E-05 | Up-Regulated |
| PDGFB | 1.021139 | 4.782006 | 3.42E-24 | 1.94E-19 | Up-Regulated |
| E2F1 | 2.083146 | 3.213491 | 3.38E-42 | 1.96E-37 | Up-Regulated |
| TIMP1 | 3.135507 | 9.292365 | 3.04E-39 | 1.76E-34 | Up-Regulated |
| MSLN | 3.41753 | 1.462707 | 1.99E-16 | 1.09E-11 | Up-Regulated |
| AQP9 | 1.449269 | 0.812422 | 3.21E-08 | 0.001586 | Up-Regulated |
| CEMIP | 1.276449 | 3.438907 | 1.63E-07 | 0.007929 | Up-Regulated |
| KCNN4 | 3.65942 | 5.544249 | 1.47E-25 | 8.34E-21 | Up-Regulated |
| BBC3 | 2.194537 | 3.902327 | 4.51E-65 | 2.63E-60 | Up-Regulated |
| TGFB1 | 1.543023 | 6.078809 | 1.93E-43 | 1.12E-38 | Up-Regulated |
| MET | 2.722577 | 8.791319 | 4.44E-45 | 2.58E-40 | Up-Regulated |
| HOXA13 | 2.99688 | -3.04243 | 4.54E-15 | 2.46E-10 | Up-Regulated |
| AGR2 | 3.578034 | 5.763443 | 3.63E-36 | 2.09E-31 | Up-Regulated |
| AHR | 1.19883 | 7.002112 | 6.47E-18 | 3.57E-13 | Up-Regulated |
| LHX2 | 5.310218 | 0.88783 | 3.05E-13 | 1.62E-08 | Up-Regulated |
| TGFBR1 | 1.776464 | 6.748425 | 1.93E-33 | 1.11E-28 | Up-Regulated |
| ENG | 1.21938 | 7.821463 | 1.34E-22 | 7.55E-18 | Up-Regulated |
| DKK1 | 2.222883 | -0.16396 | 6.73E-12 | 3.53E-07 | Up-Regulated |
| DLX4 | 1.334454 | -0.29681 | 4.18E-11 | 2.18E-06 | Up-Regulated |
| ABCC3 | 2.691879 | 6.402346 | 7.68E-43 | 4.46E-38 | Up-Regulated |
| CCND1 | 1.903563 | 8.91707 | 2.51E-62 | 1.46E-57 | Up-Regulated |
| MDK | 2.698095 | 7.405876 | 4.62E-37 | 2.67E-32 | Up-Regulated |
| LOX | 1.249841 | 4.706453 | 2.26E-07 | 0.010953 | Up-Regulated |
| SPARC | 1.1258 | 10.11626 | 1.05E-30 | 6.04E-26 | Up-Regulated |
| FGF1 | 1.564471 | 3.794787 | 7.04E-34 | 4.05E-29 | Up-Regulated |
| HRG | 2.891053 | -3.35926 | 3.84E-15 | 2.08E-10 | Up-Regulated |
| WNT5A | 1.153014 | 4.808035 | 3.86E-16 | 2.10E-11 | Up-Regulated |
| CCL20 | 2.989316 | 1.011897 | 6.92E-16 | 3.77E-11 | Up-Regulated |
| FN1 | 5.75278 | 13.70144 | 4.23E-45 | 2.46E-40 | Up-Regulated |
| SDC1 | 1.178416 | 7.317183 | 2.56E-19 | 1.42E-14 | Up-Regulated |
| EPHA4 | 1.370818 | 6.702399 | 6.81E-16 | 3.71E-11 | Up-Regulated |
| ARHGEF2 | 1.023695 | 6.88898 | 1.92E-43 | 1.12E-38 | Up-Regulated |
| MAD2L2 | 1.214986 | 4.694287 | 9.09E-34 | 5.24E-29 | Up-Regulated |
| MMP8 | 1.908523 | -3.67546 | 7.04E-07 | 0.033654 | Up-Regulated |
| NRP2 | 2.316841 | 7.453029 | 1.56E-38 | 9.05E-34 | Up-Regulated |
| SPP1 | 1.66152 | 5.752592 | 2.43E-09 | 0.000123 | Up-Regulated |
| CCND2 | 1.430832 | 8.41649 | 1.85E-44 | 1.07E-39 | Up-Regulated |
| CLU | 1.633687 | 12.69498 | 8.43E-17 | 4.62E-12 | Up-Regulated |
| TBX2 | 1.027793 | 4.393678 | 8.25E-08 | 0.004046 | Up-Regulated |
| CKS2 | 1.027448 | 3.692516 | 8.40E-16 | 4.57E-11 | Up-Regulated |
| CDKN1A | 1.11973 | 8.110369 | 2.56E-15 | 1.39E-10 | Up-Regulated |
| SOX4 | 1.792752 | 7.175548 | 2.86E-35 | 1.65E-30 | Up-Regulated |
| RUNX2 | 2.272464 | 4.126895 | 7.81E-25 | 4.42E-20 | Up-Regulated |
| HIP1 | 1.308522 | 6.744923 | 7.35E-40 | 4.26E-35 | Up-Regulated |
| MGAT3 | 3.51262 | 5.323958 | 5.24E-33 | 3.01E-28 | Up-Regulated |
| KRT17 | 4.634602 | 5.121772 | 4.58E-20 | 2.55E-15 | Up-Regulated |
| MTUS1 | 1.228618 | 6.904683 | 8.61E-32 | 4.95E-27 | Up-Regulated |
| FOXA1 | 1.784587 | -1.02009 | 9.40E-09 | 0.000471 | Up-Regulated |
| EGLN3 | 1.143559 | 2.621875 | 2.93E-07 | 0.014162 | Up-Regulated |
| GDF15 | 4.267353 | 6.594138 | 5.70E-47 | 3.32E-42 | Up-Regulated |
| LGALS3 | 2.728964 | 8.407 | 2.68E-39 | 1.55E-34 | Up-Regulated |
| ITGB4 | 2.11767 | 6.704231 | 3.19E-27 | 1.82E-22 | Up-Regulated |
| POSTN | 3.038131 | 6.700711 | 1.52E-20 | 8.49E-16 | Up-Regulated |
| KL | 1.834779 | 2.879963 | 1.33E-33 | 7.65E-29 | Up-Regulated |
| MICAL2 | 1.351757 | 5.882178 | 2.57E-18 | 1.42E-13 | Up-Regulated |
| LOXL2 | 1.921329 | 4.245884 | 4.90E-37 | 2.83E-32 | Up-Regulated |
| BHLHE40 | 1.616663 | 8.334927 | 4.82E-22 | 2.71E-17 | Up-Regulated |
| ANXA1 | 1.436307 | 10.11966 | 1.69E-18 | 9.35E-14 | Up-Regulated |
| ELF5 | 1.423127 | -2.62959 | 3.86E-09 | 0.000195 | Up-Regulated |
| CD63 | 1.017447 | 10.07519 | 3.60E-35 | 2.08E-30 | Up-Regulated |
| GLS2 | 1.740039 | 0.617016 | 2.33E-34 | 1.34E-29 | Up-Regulated |
| FHOD1 | 1.533528 | 5.918199 | 2.28E-57 | 1.33E-52 | Up-Regulated |
| SCEL | 4.23543 | 6.084027 | 6.25E-50 | 3.64E-45 | Up-Regulated |
| DAB2IP | 1.061772 | 7.005764 | 6.47E-32 | 3.72E-27 | Up-Regulated |
| TMPRSS4 | 6.324149 | 6.041356 | 3.47E-42 | 2.01E-37 | Up-Regulated |
| MMP7 | 4.124268 | 4.362854 | 2.80E-16 | 1.53E-11 | Up-Regulated |
| MMP13 | 6.751282 | 1.318273 | 2.02E-15 | 1.10E-10 | Up-Regulated |
| CYP1B1 | 3.164262 | 6.505632 | 7.74E-25 | 4.38E-20 | Up-Regulated |
| FGF2 | 1.049122 | 3.440751 | 4.08E-14 | 2.19E-09 | Up-Regulated |
| NTRK3 | 1.714022 | 3.203112 | 1.03E-13 | 5.50E-09 | Up-Regulated |
| TGFB1I1 | 1.136199 | 5.161206 | 7.36E-45 | 4.28E-40 | Up-Regulated |
| CDH11 | 1.810526 | 5.054082 | 1.16E-11 | 6.05E-07 | Up-Regulated |
| CDH13 | 2.099056 | 4.33031 | 4.44E-44 | 2.58E-39 | Up-Regulated |
| EMP3 | 1.042358 | 5.66561 | 1.10E-12 | 5.84E-08 | Up-Regulated |
| IL17RD | 2.20547 | 3.642281 | 1.17E-33 | 6.76E-29 | Up-Regulated |
| COL8A1 | 1.67724 | 8.202587 | 2.82E-33 | 1.62E-28 | Up-Regulated |
| MUC4 | 1.583726 | 1.429574 | 1.13E-08 | 0.000567 | Up-Regulated |
| CXCL14 | 4.006001 | 8.308883 | 2.51E-17 | 1.38E-12 | Up-Regulated |
| IGFBP3 | 1.78124 | 6.719266 | 1.98E-16 | 1.09E-11 | Up-Regulated |
| MSN | 1.19742 | 9.794168 | 2.39E-38 | 1.38E-33 | Up-Regulated |
| CDKN2A | 3.190506 | 2.266646 | 2.86E-29 | 1.64E-24 | Up-Regulated |
| LCN2 | 4.94726 | 5.604118 | 3.20E-31 | 1.84E-26 | Up-Regulated |
| ADAM12 | 2.542947 | 3.068775 | 2.34E-15 | 1.27E-10 | Up-Regulated |
| ADM | 2.10649 | 5.306135 | 2.82E-26 | 1.60E-21 | Up-Regulated |
| HMGA2 | 5.607711 | 5.49363 | 1.01E-58 | 5.86E-54 | Up-Regulated |
| MMP3 | 3.151172 | -1.8213 | 7.00E-08 | 0.003439 | Up-Regulated |
| EPS8 | 2.084239 | 6.945373 | 1.34E-59 | 7.84E-55 | Up-Regulated |
| SPOCK1 | 2.60483 | 5.449384 | 9.23E-25 | 5.22E-20 | Up-Regulated |
| WNT3A | 2.419505 | -2.1065 | 2.10E-08 | 0.001042 | Up-Regulated |
| TIAM1 | 3.074294 | 5.455231 | 3.78E-46 | 2.20E-41 | Up-Regulated |
| MMP14 | 1.112013 | 7.306911 | 1.96E-16 | 1.07E-11 | Up-Regulated |
| RUNX1 | 2.561753 | 5.672108 | 1.42E-38 | 8.23E-34 | Up-Regulated |
| HK2 | 1.029441 | 6.642543 | 1.12E-16 | 6.15E-12 | Up-Regulated |
| AQP5 | 2.422365 | 4.30637 | 8.20E-15 | 4.44E-10 | Up-Regulated |
| CXCL16 | 1.166999 | 5.771286 | 1.91E-27 | 1.09E-22 | Up-Regulated |
| FGF19 | 4.75781 | -3.21906 | 4.33E-11 | 2.25E-06 | Up-Regulated |
| S100A9 | 1.260159 | 3.451578 | 2.66E-07 | 0.01289 | Up-Regulated |
| TGFA | 2.671022 | 6.437983 | 2.01E-60 | 1.17E-55 | Up-Regulated |
| CLDN1 | 3.233678 | 8.593799 | 4.57E-41 | 2.65E-36 | Up-Regulated |
| CXCL5 | 4.37189 | -0.03649 | 3.49E-21 | 1.95E-16 | Up-Regulated |
| SPRY1 | 1.237077 | 6.693486 | 4.54E-22 | 2.55E-17 | Up-Regulated |
| MST1R | 1.700888 | 3.691942 | 2.73E-24 | 1.54E-19 | Up-Regulated |
| ITGA2 | 3.05814 | 6.204706 | 5.28E-38 | 3.05E-33 | Up-Regulated |
| F2RL2 | 2.492604 | 0.419084 | 7.81E-18 | 4.31E-13 | Up-Regulated |
| FOXQ1 | 2.164543 | 5.097069 | 1.18E-19 | 6.56E-15 | Up-Regulated |
| CSF2 | 5.615484 | 0.266827 | 4.93E-42 | 2.86E-37 | Up-Regulated |
| SHH | 3.258091 | -1.40427 | 1.95E-11 | 1.02E-06 | Up-Regulated |
| MICALL2 | 1.062148 | 4.662519 | 1.50E-27 | 8.55E-23 | Up-Regulated |
| CTHRC1 | 2.362529 | 3.827306 | 4.10E-15 | 2.22E-10 | Up-Regulated |
| ANPEP | 1.156567 | 3.746307 | 3.41E-08 | 0.001685 | Up-Regulated |
| PBX3 | 1.229262 | 7.348128 | 2.54E-11 | 1.33E-06 | Up-Regulated |
| KLK6 | 6.82606 | 2.236019 | 5.13E-26 | 2.91E-21 | Up-Regulated |
| ANGPTL4 | 1.545133 | 5.670463 | 1.46E-15 | 7.92E-11 | Up-Regulated |
| SEMA4C | 1.163852 | 5.517179 | 7.95E-46 | 4.62E-41 | Up-Regulated |
| CXCL8 | 1.99295 | 3.026966 | 8.36E-12 | 4.39E-07 | Up-Regulated |
| SEMA3E | 1.802228 | 2.724432 | 1.72E-10 | 8.86E-06 | Up-Regulated |
| CDH2 | 3.041495 | 5.871591 | 7.45E-28 | 4.25E-23 | Up-Regulated |
| HS6ST2 | 3.66169 | 3.969055 | 3.29E-18 | 1.82E-13 | Up-Regulated |
| ALK | 4.533222 | 3.02654 | 2.51E-28 | 1.44E-23 | Up-Regulated |
| LRG1 | 3.527293 | 3.57906 | 1.51E-36 | 8.73E-32 | Up-Regulated |
| KRT19 | 2.831135 | 8.226255 | 6.09E-26 | 3.46E-21 | Up-Regulated |
| BCL2L1 | 1.28872 | 7.114985 | 4.73E-35 | 2.73E-30 | Up-Regulated |
| COL8A2 | 1.946255 | 6.797226 | 4.61E-19 | 2.56E-14 | Up-Regulated |
| HPSE | 1.286075 | 2.48394 | 1.98E-13 | 1.06E-08 | Up-Regulated |
| CSPG4 | 1.655361 | 4.476867 | 3.09E-28 | 1.76E-23 | Up-Regulated |
| UCP2 | 1.123328 | 8.13253 | 6.04E-18 | 3.33E-13 | Up-Regulated |
| ETV4 | 3.435957 | 5.456567 | 1.30E-74 | 7.58E-70 | Up-Regulated |
| SPHK1 | 1.358619 | 4.335731 | 4.74E-22 | 2.66E-17 | Up-Regulated |
| GRIN1 | 2.220081 | -1.25928 | 1.10E-17 | 6.09E-13 | Up-Regulated |
| TYMS | 1.171293 | 3.395609 | 3.52E-23 | 1.98E-18 | Up-Regulated |
| CD151 | 1.309487 | 8.336701 | 2.42E-39 | 1.40E-34 | Up-Regulated |
| F2R | 1.496941 | 5.47303 | 2.40E-32 | 1.38E-27 | Up-Regulated |
| TNFSF15 | 1.968646 | 3.772865 | 2.16E-27 | 1.23E-22 | Up-Regulated |
| PHLDA2 | 1.934261 | 4.111004 | 4.57E-25 | 2.59E-20 | Up-Regulated |
| EPHB3 | 3.599485 | 4.374055 | 1.69E-48 | 9.86E-44 | Up-Regulated |
| MACC1 | 1.799031 | 4.44679 | 6.46E-16 | 3.52E-11 | Up-Regulated |
| PTP4A3 | 2.019687 | 5.605357 | 4.37E-58 | 2.54E-53 | Up-Regulated |
| JAG2 | 1.555012 | 4.915168 | 3.14E-57 | 1.83E-52 | Up-Regulated |
| WT1 | 2.080662 | -2.39051 | 1.18E-07 | 0.005758 | Up-Regulated |
| ROR1 | 1.512604 | 2.000944 | 3.46E-11 | 1.80E-06 | Up-Regulated |
| MUC1 | 2.522309 | 6.895371 | 3.13E-18 | 1.73E-13 | Up-Regulated |
| CYP4Z1 | 1.301985 | -2.73403 | 6.30E-07 | 0.030159 | Up-Regulated |
| NANOS3 | 1.364798 | 0.127555 | 2.50E-18 | 1.38E-13 | Up-Regulated |
| S100A4 | 1.82359 | 7.412216 | 1.45E-13 | 7.77E-09 | Up-Regulated |
| AJAP1 | 1.292028 | 2.392891 | 4.92E-09 | 0.000248 | Up-Regulated |
| MMP1 | 3.457664 | 0.340755 | 2.14E-21 | 1.20E-16 | Up-Regulated |
| S100A2 | 2.222776 | 5.035546 | 5.71E-14 | 3.07E-09 | Up-Regulated |
| FLNA | 1.02027 | 9.650373 | 1.26E-17 | 6.95E-13 | Up-Regulated |
| S100A6 | 2.059505 | 11.01035 | 1.04E-26 | 5.89E-22 | Up-Regulated |
| TGM2 | 1.915899 | 7.158108 | 8.25E-22 | 4.63E-17 | Up-Regulated |
| MIR222 | 1.848044 | -3.26765 | 1.36E-09 | 6.89E-05 | Up-Regulated |
| MIR34A | 1.66767 | -3.15102 | 2.56E-18 | 1.41E-13 | Up-Regulated |
| MIR221 | 1.944659 | -0.75664 | 2.63E-12 | 1.39E-07 | Up-Regulated |
| HOXA10 | 1.969165 | -1.70931 | 4.66E-08 | 0.002301 | Up-Regulated |
| UHRF1 | 1.059934 | 1.461334 | 1.29E-09 | 6.57E-05 | Up-Regulated |
| UCA1 | 3.138195 | -2.54566 | 3.94E-11 | 2.05E-06 | Up-Regulated |
| PROM1 | -1.97118 | 2.344763 | 2.67E-15 | 1.45E-10 | Down-Regulated |
| CNTN1 | -1.44105 | 0.704231 | 6.73E-08 | 0.003308 | Down-Regulated |
| RUNX3 | -1.00623 | 2.783695 | 5.47E-08 | 0.002696 | Down-Regulated |
| FHL1 | -1.57452 | 7.952275 | 6.52E-22 | 3.66E-17 | Down-Regulated |
| NTN1 | -1.1308 | 1.429125 | 3.30E-12 | 1.74E-07 | Down-Regulated |
| PRKCQ | -1.14409 | 4.489206 | 1.35E-47 | 7.85E-43 | Down-Regulated |
| FGFR2 | -1.06185 | 6.054531 | 1.57E-29 | 8.97E-25 | Down-Regulated |
| KLF6 | -1.22099 | 8.212938 | 4.19E-17 | 2.30E-12 | Down-Regulated |
| HDAC4 | -1.15217 | 4.017596 | 3.80E-63 | 2.22E-58 | Down-Regulated |
| TGFBR3 | -1.0042 | 4.222611 | 2.66E-31 | 1.53E-26 | Down-Regulated |
| FBLN1 | -1.71324 | 5.976957 | 4.90E-19 | 2.72E-14 | Down-Regulated |
| WNT11 | -1.80913 | 1.235146 | 6.25E-21 | 3.49E-16 | Down-Regulated |
| FKBP5 | -1.15606 | 6.634277 | 6.36E-17 | 3.49E-12 | Down-Regulated |
| SALL4 | -1.00407 | 1.169686 | 1.02E-09 | 5.18E-05 | Down-Regulated |
| FERMT1 | -1.21478 | 2.275914 | 4.39E-12 | 2.31E-07 | Down-Regulated |
| TLE4 | -1.50183 | 3.579046 | 5.68E-46 | 3.30E-41 | Down-Regulated |
| CCL2 | -1.11183 | 4.038585 | 1.82E-10 | 9.38E-06 | Down-Regulated |
| FOXN1 | -1.24607 | -0.98924 | 5.89E-12 | 3.10E-07 | Down-Regulated |
| PPARGC1A | -1.72902 | 4.928908 | 2.98E-21 | 1.67E-16 | Down-Regulated |
| CRYAB | -1.21726 | 7.457838 | 3.84E-18 | 2.12E-13 | Down-Regulated |
| GLI1 | -2.18021 | -0.59238 | 5.47E-39 | 3.17E-34 | Down-Regulated |
| SRF | -1.40398 | 6.141176 | 3.61E-69 | 2.10E-64 | Down-Regulated |
| EFEMP1 | -1.61 | 6.419606 | 7.57E-30 | 4.33E-25 | Down-Regulated |
| FHL2 | -1.19236 | 4.372478 | 1.94E-20 | 1.08E-15 | Down-Regulated |
| PROX1 | -1.97505 | 3.454965 | 4.37E-49 | 2.54E-44 | Down-Regulated |
| CTGF | -1.46886 | 8.773304 | 9.09E-21 | 5.08E-16 | Down-Regulated |
| SMAD9 | -1.41792 | 5.498426 | 5.68E-28 | 3.24E-23 | Down-Regulated |
| EGR1 | -1.64571 | 9.702856 | 6.78E-31 | 3.89E-26 | Down-Regulated |
| NR4A1 | -1.46334 | 7.775022 | 4.14E-15 | 2.24E-10 | Down-Regulated |
| SNAI1 | -1.12827 | 2.917342 | 2.03E-16 | 1.11E-11 | Down-Regulated |
| FOXA2 | -1.95019 | 2.3107 | 1.69E-17 | 9.32E-13 | Down-Regulated |
| BMP2 | -1.94023 | 5.099294 | 1.79E-55 | 1.04E-50 | Down-Regulated |
| CCR7 | -1.54776 | 2.147406 | 2.22E-14 | 1.20E-09 | Down-Regulated |
| FOXP2 | -1.84903 | 2.937237 | 8.79E-28 | 5.01E-23 | Down-Regulated |
| WASF3 | -1.29252 | 4.634867 | 1.64E-28 | 9.39E-24 | Down-Regulated |
| SOX5 | -1.57114 | 1.003203 | 7.45E-42 | 4.32E-37 | Down-Regulated |
| IL6 | -1.3244 | 2.242626 | 3.86E-07 | 0.018578 | Down-Regulated |
| CCL21 | -3.1896 | 5.519708 | 3.55E-29 | 2.03E-24 | Down-Regulated |
| FBLN5 | -1.10947 | 4.873987 | 4.41E-27 | 2.51E-22 | Down-Regulated |
| IRF8 | -1.47895 | 3.507662 | 7.22E-17 | 3.96E-12 | Down-Regulated |
| CYR61 | -1.68304 | 8.429421 | 7.33E-30 | 4.20E-25 | Down-Regulated |
| LRIG1 | -1.3264 | 5.422739 | 1.13E-52 | 6.58E-48 | Down-Regulated |
| SCUBE3 | -1.32316 | 6.21314 | 7.07E-18 | 3.90E-13 | Down-Regulated |
| GPC3 | -3.06637 | 1.862801 | 5.41E-67 | 3.16E-62 | Down-Regulated |
| KIT | -1.94252 | 5.336668 | 2.29E-23 | 1.29E-18 | Down-Regulated |
| FCN2 | -2.07653 | -3.7431 | 8.48E-19 | 4.70E-14 | Down-Regulated |
| PTX3 | -1.28633 | 0.253883 | 3.60E-28 | 2.06E-23 | Down-Regulated |
| ALDH1A1 | -1.18774 | 7.516468 | 2.40E-20 | 1.34E-15 | Down-Regulated |
| TNXB | -1.40795 | 4.199829 | 5.24E-30 | 3.00E-25 | Down-Regulated |
| IRS1 | -1.30693 | 6.362521 | 9.77E-28 | 5.57E-23 | Down-Regulated |
| ROR2 | -1.42741 | 3.663505 | 1.37E-15 | 7.43E-11 | Down-Regulated |
| AR | -1.04092 | 3.583235 | 6.49E-10 | 3.32E-05 | Down-Regulated |
| BCL2 | -1.39837 | 6.224847 | 6.00E-37 | 3.47E-32 | Down-Regulated |
| CCL19 | -1.9137 | 3.884259 | 2.57E-09 | 0.00013 | Down-Regulated |
| CYP7B1 | -1.45227 | 2.022873 | 1.58E-38 | 9.13E-34 | Down-Regulated |
| PCSK1 | -1.13829 | -1.35177 | 6.02E-13 | 3.19E-08 | Down-Regulated |

| Table S4. Cox survival results | | | | | | |
| --- | --- | --- | --- | --- | --- | --- |
| Terms | HR[exp(coef)] | coef | 95% CI lower | 95% CI upper | Z | P value |
| FHL1 | 0.7268 | -0.3191 | -0.5218 | -0.1164 | -3.0862 | 0.0020 |
| TGFBR3 | 0.5336 | -0.6281 | -1.0898 | -0.1664 | -2.6664 | 0.0077 |
| CTGF | 0.7532 | -0.2834 | -0.4937 | -0.0731 | -2.6413 | 0.0083 |
| FOXP2 | 0.4859 | -0.7218 | -1.2599 | -0.1837 | -2.6291 | 0.0086 |
| FBLN5 | 0.6369 | -0.4512 | -0.8060 | -0.0964 | -2.4924 | 0.0127 |
| WT1 | 2.2997 | 0.8328 | 0.1289 | 1.5367 | 2.3189 | 0.0204 |
| IL11 | 1.6419 | 0.4958 | 0.0631 | 0.9285 | 2.2460 | 0.0247 |
| EFEMP1 | 0.7981 | -0.2255 | -0.4241 | -0.0269 | -2.2256 | 0.0260 |
| EGR1 | 0.7947 | -0.2298 | -0.4355 | -0.0241 | -2.1900 | 0.0285 |
| JUN | 0.7377 | -0.3042 | -0.5822 | -0.0263 | -2.1454 | 0.0319 |
| WNT11 | 0.6538 | -0.4250 | -0.8280 | -0.0220 | -2.0670 | 0.0387 |
| FOXA2 | 0.7709 | -0.2602 | -0.5096 | -0.0109 | -2.0458 | 0.0408 |
| ALDH1A1 | 0.7994 | -0.2239 | -0.4391 | -0.0087 | -2.0389 | 0.0415 |
| PROX1 | 0.6781 | -0.3884 | -0.7624 | -0.0145 | -2.0358 | 0.0418 |
| AQP9 | 1.4249 | 0.3541 | 0.0131 | 0.6951 | 2.0354 | 0.0418 |
| LCN2 | 1.1542 | 0.1434 | 0.0050 | 0.2819 | 2.0305 | 0.0423 |
| UHRF1 | 1.6816 | 0.5198 | 0.0072 | 1.0324 | 1.9873 | 0.0469 |
| PRKCQ | 0.6698 | -0.4008 | -0.8006 | -0.0009 | -1.9645 | 0.0495 |
| PDGFB | 0.6562 | -0.4213 | -0.8463 | 0.0038 | -1.9423 | 0.0521 |
| MAD2L2 | 1.7767 | 0.5747 | -0.0122 | 1.1616 | 1.9193 | 0.0549 |
| CYR61 | 0.7926 | -0.2324 | -0.4711 | 0.0063 | -1.9083 | 0.0564 |
| FHOD1 | 1.6616 | 0.5078 | -0.0153 | 1.0308 | 1.9026 | 0.0571 |
| NR4A1 | 0.8292 | -0.1872 | -0.3866 | 0.0121 | -1.8409 | 0.0656 |
| CKS2 | 1.4704 | 0.3855 | -0.0327 | 0.8037 | 1.8067 | 0.0708 |
| KIT | 0.8234 | -0.1943 | -0.4052 | 0.0166 | -1.8060 | 0.0709 |
| SOX5 | 0.4033 | -0.9082 | -1.8941 | 0.0778 | -1.8054 | 0.0710 |
| CDKN2A | 1.2746 | 0.2426 | -0.0232 | 0.5084 | 1.7890 | 0.0736 |
| GRIN1 | 2.1196 | 0.7512 | -0.0802 | 1.5826 | 1.7709 | 0.0766 |
| PLAUR | 1.2319 | 0.2086 | -0.0258 | 0.4430 | 1.7441 | 0.0811 |
| WNT3A | 1.4231 | 0.3529 | -0.0454 | 0.7511 | 1.7365 | 0.0825 |
| EPHB3 | 1.2490 | 0.2224 | -0.0290 | 0.4737 | 1.7341 | 0.0829 |
| ROR2 | 0.7995 | -0.2238 | -0.4768 | 0.0292 | -1.7340 | 0.0829 |
| IRS1 | 0.7812 | -0.2470 | -0.5285 | 0.0346 | -1.7192 | 0.0856 |
| TDGF1 | 0.3443 | -1.0662 | -2.2847 | 0.1523 | -1.7150 | 0.0863 |
| MMP13 | 1.1795 | 0.1651 | -0.0240 | 0.3542 | 1.7117 | 0.0870 |
| ITGA2 | 1.2043 | 0.1859 | -0.0279 | 0.3997 | 1.7044 | 0.0883 |
| FOXQ1 | 1.1985 | 0.1811 | -0.0285 | 0.3907 | 1.6936 | 0.0903 |
| SPHK1 | 1.4025 | 0.3383 | -0.0631 | 0.7397 | 1.6520 | 0.0985 |
| TLE4 | 0.7085 | -0.3446 | -0.7535 | 0.0643 | -1.6517 | 0.0986 |
| LRIG1 | 0.6967 | -0.3614 | -0.7912 | 0.0683 | -1.6484 | 0.0993 |
| FHL2 | 0.7495 | -0.2884 | -0.6318 | 0.0551 | -1.6456 | 0.0998 |
| AQP5 | 0.8735 | -0.1352 | -0.2967 | 0.0263 | -1.6410 | 0.1008 |
| FN1 | 1.1153 | 0.1092 | -0.0212 | 0.2396 | 1.6408 | 0.1008 |
| TYMS | 1.4833 | 0.3942 | -0.0820 | 0.8705 | 1.6225 | 0.1047 |
| GPC3 | 0.7625 | -0.2712 | -0.6022 | 0.0598 | -1.6058 | 0.1083 |
| ENG | 0.7487 | -0.2894 | -0.6443 | 0.0655 | -1.5983 | 0.1100 |
| CEACAM6 | 1.1223 | 0.1154 | -0.0264 | 0.2571 | 1.5949 | 0.1107 |
| FGFR2 | 0.7674 | -0.2647 | -0.5908 | 0.0615 | -1.5906 | 0.1117 |
| CXCL16 | 1.5084 | 0.4110 | -0.0974 | 0.9195 | 1.5846 | 0.1131 |
| MICALL2 | 1.5323 | 0.4267 | -0.1042 | 0.9577 | 1.5754 | 0.1152 |
| SPP1 | 1.1533 | 0.1426 | -0.0363 | 0.3215 | 1.5624 | 0.1182 |
| F2R | 0.7706 | -0.2606 | -0.5929 | 0.0717 | -1.5370 | 0.1243 |
| SALL4 | 1.3111 | 0.2709 | -0.0820 | 0.6238 | 1.5046 | 0.1324 |
| CX3CL1 | 0.7373 | -0.3047 | -0.7035 | 0.0941 | -1.4976 | 0.1342 |
| E2F1 | 1.3561 | 0.3046 | -0.0960 | 0.7053 | 1.4902 | 0.1362 |
| CCL20 | 1.1699 | 0.1569 | -0.0525 | 0.3663 | 1.4689 | 0.1419 |
| LOX | 1.1709 | 0.1578 | -0.0533 | 0.3689 | 1.4653 | 0.1428 |
| MUC1 | 1.1440 | 0.1346 | -0.0456 | 0.3148 | 1.4637 | 0.1433 |
| S100A6 | 1.2316 | 0.2083 | -0.0713 | 0.4880 | 1.4600 | 0.1443 |
| MDK | 1.1769 | 0.1629 | -0.0615 | 0.3872 | 1.4230 | 0.1547 |
| POSTN | 1.1258 | 0.1185 | -0.0453 | 0.2823 | 1.4175 | 0.1563 |
| SOX4 | 1.2626 | 0.2332 | -0.0926 | 0.5590 | 1.4030 | 0.1606 |
| EGLN3 | 1.2584 | 0.2298 | -0.0918 | 0.5515 | 1.4003 | 0.1614 |
| LAMC2 | 1.2817 | 0.2482 | -0.1021 | 0.5985 | 1.3888 | 0.1649 |
| ITGA3 | 1.2883 | 0.2533 | -0.1072 | 0.6139 | 1.3770 | 0.1685 |
| WASF3 | 0.7998 | -0.2235 | -0.5423 | 0.0954 | -1.3737 | 0.1695 |
| BMP2 | 0.8115 | -0.2089 | -0.5083 | 0.0905 | -1.3676 | 0.1714 |
| GAB2 | 1.3433 | 0.2951 | -0.1288 | 0.7190 | 1.3645 | 0.1724 |
| HDAC4 | 0.6506 | -0.4299 | -1.0477 | 0.1880 | -1.3637 | 0.1727 |
| MMP8 | 2.8221 | 1.0375 | -0.4622 | 2.5371 | 1.3559 | 0.1751 |
| ADAM12 | 1.1833 | 0.1683 | -0.0821 | 0.4186 | 1.3174 | 0.1877 |
| CYP7B1 | 0.7861 | -0.2407 | -0.6100 | 0.1286 | -1.2777 | 0.2014 |
| JAG2 | 0.7237 | -0.3234 | -0.8223 | 0.1755 | -1.2707 | 0.2039 |
| WWOX | 0.7261 | -0.3201 | -0.8193 | 0.1792 | -1.2565 | 0.2089 |
| HMGA2 | 1.1563 | 0.1452 | -0.0821 | 0.3726 | 1.2520 | 0.2106 |
| TLE1 | 0.7476 | -0.2909 | -0.7504 | 0.1686 | -1.2407 | 0.2147 |
| TYRO3 | 1.2941 | 0.2578 | -0.1521 | 0.6677 | 1.2326 | 0.2177 |
| KL | 0.8026 | -0.2199 | -0.5745 | 0.1347 | -1.2153 | 0.2243 |
| PPARGC1A | 0.8591 | -0.1518 | -0.3977 | 0.0940 | -1.2104 | 0.2261 |
| ANGPTL4 | 0.8607 | -0.1500 | -0.3956 | 0.0956 | -1.1969 | 0.2313 |
| HPSE | 1.2311 | 0.2079 | -0.1342 | 0.5499 | 1.1911 | 0.2336 |
| SMAD9 | 0.8456 | -0.1677 | -0.4437 | 0.1083 | -1.1911 | 0.2336 |
| ROR1 | 1.2340 | 0.2102 | -0.1385 | 0.5590 | 1.1816 | 0.2374 |
| CTHRC1 | 1.1116 | 0.1058 | -0.0707 | 0.2823 | 1.1751 | 0.2399 |
| TNXB | 0.7907 | -0.2349 | -0.6293 | 0.1596 | -1.1672 | 0.2431 |
| SPRY1 | 0.8351 | -0.1802 | -0.4832 | 0.1229 | -1.1652 | 0.2439 |
| TNC | 1.1107 | 0.1050 | -0.0732 | 0.2833 | 1.1549 | 0.2481 |
| CSPG4 | 0.8122 | -0.2080 | -0.5621 | 0.1460 | -1.1516 | 0.2495 |
| PLXND1 | 0.7783 | -0.2506 | -0.6794 | 0.1782 | -1.1454 | 0.2520 |
| FOXN1 | 0.5373 | -0.6211 | -1.6881 | 0.4458 | -1.1411 | 0.2538 |
| MMP1 | 1.1697 | 0.1568 | -0.1129 | 0.4265 | 1.1392 | 0.2546 |
| NANOS3 | 1.2773 | 0.2448 | -0.1810 | 0.6705 | 1.1266 | 0.2599 |
| KRT19 | 1.1053 | 0.1001 | -0.0744 | 0.2747 | 1.1241 | 0.2610 |
| ARHGEF2 | 1.3851 | 0.3257 | -0.2475 | 0.8990 | 1.1137 | 0.2654 |
| CRYAB | 0.8615 | -0.1490 | -0.4121 | 0.1141 | -1.1102 | 0.2669 |
| NTN1 | 0.7165 | -0.3333 | -0.9330 | 0.2663 | -1.0896 | 0.2759 |
| BCL2 | 0.8438 | -0.1699 | -0.4757 | 0.1360 | -1.0885 | 0.2764 |
| CCND2 | 0.8295 | -0.1869 | -0.5245 | 0.1507 | -1.0851 | 0.2779 |
| CNTN1 | 0.6815 | -0.3835 | -1.0788 | 0.3118 | -1.0810 | 0.2797 |
| MICAL2 | 1.1902 | 0.1741 | -0.1525 | 0.5007 | 1.0450 | 0.2960 |
| SRF | 0.7583 | -0.2766 | -0.7961 | 0.2429 | -1.0437 | 0.2966 |
| MCAM | 0.8239 | -0.1937 | -0.5659 | 0.1785 | -1.0200 | 0.3077 |
| MST1R | 1.1859 | 0.1705 | -0.1582 | 0.4992 | 1.0167 | 0.3093 |
| MACC1 | 1.1461 | 0.1363 | -0.1268 | 0.3995 | 1.0154 | 0.3099 |
| TGFB1 | 1.2460 | 0.2200 | -0.2060 | 0.6459 | 1.0120 | 0.3115 |
| BBC3 | 1.2632 | 0.2337 | -0.2207 | 0.6881 | 1.0079 | 0.3135 |
| TMPRSS4 | 1.0799 | 0.0768 | -0.0735 | 0.2271 | 1.0020 | 0.3164 |
| IGFBP3 | 1.1228 | 0.1158 | -0.1129 | 0.3446 | 0.9924 | 0.3210 |
| ADM | 1.1433 | 0.1339 | -0.1335 | 0.4014 | 0.9815 | 0.3263 |
| CAMK1D | 0.8264 | -0.1907 | -0.5737 | 0.1924 | -0.9754 | 0.3293 |
| S100A9 | 1.1127 | 0.1067 | -0.1086 | 0.3221 | 0.9715 | 0.3313 |
| SPARC | 0.8205 | -0.1978 | -0.5988 | 0.2031 | -0.9672 | 0.3334 |
| HOXA13 | 0.0574 | -2.8572 | -8.6795 | 2.9651 | -0.9618 | 0.3361 |
| FCN2 | 0.4092 | -0.8936 | -2.7323 | 0.9451 | -0.9525 | 0.3408 |
| CDH13 | 0.8378 | -0.1770 | -0.5431 | 0.1892 | -0.9473 | 0.3435 |
| ELF5 | 0.3185 | -1.1442 | -3.5309 | 1.2425 | -0.9396 | 0.3474 |
| CXCL14 | 0.9582 | -0.0427 | -0.1350 | 0.0497 | -0.9053 | 0.3653 |
| HS6ST2 | 0.9244 | -0.0786 | -0.2503 | 0.0932 | -0.8966 | 0.3699 |
| SEMA3E | 0.8791 | -0.1289 | -0.4117 | 0.1539 | -0.8935 | 0.3716 |
| PCSK1 | 0.4459 | -0.8076 | -2.5794 | 0.9643 | -0.8933 | 0.3717 |
| TGM2 | 1.1271 | 0.1197 | -0.1434 | 0.3828 | 0.8917 | 0.3726 |
| AR | 0.8517 | -0.1605 | -0.5135 | 0.1925 | -0.8913 | 0.3728 |
| MSLN | 0.8860 | -0.1210 | -0.3908 | 0.1487 | -0.8793 | 0.3793 |
| FOXA1 | 1.2477 | 0.2213 | -0.2753 | 0.7180 | 0.8735 | 0.3824 |
| LTBP1 | 1.1127 | 0.1068 | -0.1355 | 0.3490 | 0.8640 | 0.3876 |
| IL6 | 0.9076 | -0.0970 | -0.3173 | 0.1234 | -0.8626 | 0.3883 |
| TGFB2 | 0.8922 | -0.1141 | -0.3744 | 0.1462 | -0.8590 | 0.3904 |
| MMP7 | 1.0611 | 0.0593 | -0.0772 | 0.1957 | 0.8515 | 0.3945 |
| MET | 1.1119 | 0.1061 | -0.1408 | 0.3529 | 0.8422 | 0.3997 |
| CCL21 | 0.9543 | -0.0468 | -0.1577 | 0.0642 | -0.8256 | 0.4090 |
| CXCL8 | 1.0868 | 0.0832 | -0.1147 | 0.2812 | 0.8242 | 0.4098 |
| RUNX1 | 1.1314 | 0.1234 | -0.1710 | 0.4179 | 0.8218 | 0.4112 |
| MSN | 1.2141 | 0.1940 | -0.2752 | 0.6632 | 0.8105 | 0.4177 |
| KCNN4 | 1.0646 | 0.0626 | -0.0895 | 0.2146 | 0.8066 | 0.4199 |
| TBX2 | 0.8830 | -0.1245 | -0.4302 | 0.1813 | -0.7979 | 0.4249 |
| FERMT1 | 0.8624 | -0.1481 | -0.5142 | 0.2180 | -0.7928 | 0.4279 |
| ETV4 | 1.1228 | 0.1159 | -0.1754 | 0.4071 | 0.7795 | 0.4357 |
| SNAI1 | 0.8923 | -0.1140 | -0.4028 | 0.1748 | -0.7737 | 0.4391 |
| MMP11 | 1.0892 | 0.0854 | -0.1324 | 0.3032 | 0.7686 | 0.4422 |
| ABCC3 | 1.0976 | 0.0931 | -0.1524 | 0.3386 | 0.7435 | 0.4572 |
| KRT17 | 0.9480 | -0.0534 | -0.1943 | 0.0875 | -0.7428 | 0.4576 |
| CYP4Z1 | 1.5191 | 0.4181 | -0.6865 | 1.5227 | 0.7419 | 0.4582 |
| CXCL5 | 1.1266 | 0.1192 | -0.1965 | 0.4349 | 0.7398 | 0.4594 |
| LGALS3 | 1.0867 | 0.0832 | -0.1376 | 0.3039 | 0.7384 | 0.4603 |
| CLDN1 | 1.0737 | 0.0711 | -0.1213 | 0.2634 | 0.7242 | 0.4689 |
| TIMP1 | 1.0745 | 0.0719 | -0.1230 | 0.2668 | 0.7227 | 0.4699 |
| CDKN1A | 0.8847 | -0.1225 | -0.4574 | 0.2125 | -0.7168 | 0.4735 |
| FLNA | 1.1367 | 0.1281 | -0.2238 | 0.4800 | 0.7134 | 0.4756 |
| GLI1 | 0.7447 | -0.2947 | -1.1135 | 0.5241 | -0.7055 | 0.4805 |
| HRG | 0.1577 | -1.8469 | -7.0043 | 3.3105 | -0.7019 | 0.4828 |
| SCUBE3 | 0.9243 | -0.0787 | -0.2992 | 0.1418 | -0.6996 | 0.4842 |
| NTRK3 | 0.8616 | -0.1489 | -0.5761 | 0.2782 | -0.6833 | 0.4944 |
| EPHA4 | 1.0948 | 0.0906 | -0.1714 | 0.3525 | 0.6776 | 0.4980 |
| MMP14 | 1.1220 | 0.1151 | -0.2193 | 0.4495 | 0.6748 | 0.4998 |
| KLF6 | 0.9202 | -0.0831 | -0.3259 | 0.1597 | -0.6710 | 0.5022 |
| S100A2 | 1.0672 | 0.0650 | -0.1260 | 0.2560 | 0.6674 | 0.5045 |
| VCAN | 1.0715 | 0.0691 | -0.1366 | 0.2747 | 0.6583 | 0.5103 |
| BCL2L1 | 1.1736 | 0.1601 | -0.3228 | 0.6430 | 0.6496 | 0.5159 |
| CSF2 | 1.0869 | 0.0833 | -0.1689 | 0.3355 | 0.6476 | 0.5173 |
| HDAC9 | 1.1238 | 0.1167 | -0.2407 | 0.4742 | 0.6401 | 0.5221 |
| GDF15 | 1.0656 | 0.0635 | -0.1317 | 0.2587 | 0.6376 | 0.5238 |
| FBLN1 | 0.9395 | -0.0624 | -0.2594 | 0.1346 | -0.6209 | 0.5346 |
| CEMIP | 0.9137 | -0.0902 | -0.3817 | 0.2013 | -0.6065 | 0.5442 |
| WNT5A | 0.9107 | -0.0936 | -0.3991 | 0.2120 | -0.6001 | 0.5484 |
| AGR2 | 1.0515 | 0.0502 | -0.1206 | 0.2209 | 0.5760 | 0.5646 |
| TGFB1I1 | 1.1726 | 0.1592 | -0.3914 | 0.7099 | 0.5668 | 0.5708 |
| DKK1 | 1.1133 | 0.1073 | -0.2643 | 0.4790 | 0.5660 | 0.5714 |
| SEMA4C | 1.1535 | 0.1428 | -0.3567 | 0.6422 | 0.5603 | 0.5753 |
| SREBF1 | 1.1166 | 0.1103 | -0.3191 | 0.5398 | 0.5034 | 0.6147 |
| SDC1 | 1.1040 | 0.0989 | -0.2884 | 0.4862 | 0.5005 | 0.6167 |
| CD44 | 1.0930 | 0.0890 | -0.2751 | 0.4530 | 0.4790 | 0.6320 |
| LGALS1 | 0.9385 | -0.0635 | -0.3257 | 0.1987 | -0.4747 | 0.6350 |
| CCR7 | 1.0704 | 0.0680 | -0.2169 | 0.3529 | 0.4678 | 0.6400 |
| TRPC5 | 0.9601 | -0.0408 | -0.2128 | 0.1313 | -0.4643 | 0.6424 |
| MIR221 | 1.0425 | 0.0416 | -0.1352 | 0.2184 | 0.4614 | 0.6445 |
| S100A4 | 1.0457 | 0.0446 | -0.1474 | 0.2367 | 0.4556 | 0.6487 |
| MGAT3 | 1.0411 | 0.0402 | -0.1357 | 0.2162 | 0.4483 | 0.6539 |
| UCA1 | 1.2135 | 0.1935 | -0.6533 | 1.0404 | 0.4479 | 0.6542 |
| LOXL2 | 1.0899 | 0.0861 | -0.2911 | 0.4632 | 0.4473 | 0.6547 |
| PBX3 | 0.9459 | -0.0556 | -0.2994 | 0.1882 | -0.4467 | 0.6551 |
| RUNX3 | 1.0673 | 0.0651 | -0.2290 | 0.3592 | 0.4338 | 0.6644 |
| RUNX2 | 0.9487 | -0.0527 | -0.2993 | 0.1939 | -0.4188 | 0.6753 |
| ANXA1 | 1.0533 | 0.0520 | -0.1929 | 0.2969 | 0.4159 | 0.6775 |
| EMP3 | 1.0699 | 0.0676 | -0.2668 | 0.4019 | 0.3959 | 0.6922 |
| FKBP5 | 0.9415 | -0.0603 | -0.3678 | 0.2473 | -0.3840 | 0.7010 |
| HOXA10 | 0.7013 | -0.3548 | -2.2023 | 1.4927 | -0.3764 | 0.7066 |
| ITGB4 | 1.0458 | 0.0448 | -0.2000 | 0.2895 | 0.3586 | 0.7199 |
| TP63 | 0.9489 | -0.0524 | -0.3545 | 0.2497 | -0.3400 | 0.7339 |
| TGFA | 1.0476 | 0.0465 | -0.2281 | 0.3210 | 0.3317 | 0.7401 |
| CLU | 0.9709 | -0.0295 | -0.2087 | 0.1497 | -0.3228 | 0.7468 |
| SCEL | 0.9723 | -0.0281 | -0.2002 | 0.1440 | -0.3197 | 0.7492 |
| HIP1 | 1.0694 | 0.0671 | -0.3509 | 0.4851 | 0.3148 | 0.7529 |
| LRG1 | 0.9679 | -0.0326 | -0.2385 | 0.1733 | -0.3104 | 0.7562 |
| PTP4A3 | 1.0701 | 0.0677 | -0.3692 | 0.5046 | 0.3038 | 0.7613 |
| MTUS1 | 1.0607 | 0.0589 | -0.3257 | 0.4435 | 0.3001 | 0.7641 |
| FGF2 | 1.0591 | 0.0574 | -0.3347 | 0.4496 | 0.2870 | 0.7741 |
| MRC2 | 1.0294 | 0.0289 | -0.1691 | 0.2270 | 0.2865 | 0.7745 |
| MIR222 | 1.0400 | 0.0392 | -0.2357 | 0.3142 | 0.2797 | 0.7797 |
| CYP1B1 | 1.0207 | 0.0204 | -0.1362 | 0.1771 | 0.2558 | 0.7981 |
| CD151 | 0.9467 | -0.0548 | -0.4889 | 0.3793 | -0.2473 | 0.8047 |
| EHD2 | 1.0610 | 0.0592 | -0.4112 | 0.5297 | 0.2468 | 0.8051 |
| TIAM1 | 0.9718 | -0.0286 | -0.2653 | 0.2081 | -0.2365 | 0.8130 |
| GLS2 | 1.0775 | 0.0747 | -0.5443 | 0.6937 | 0.2364 | 0.8131 |
| ETV1 | 1.0417 | 0.0408 | -0.2994 | 0.3811 | 0.2353 | 0.8140 |
| HMOX1 | 0.9673 | -0.0333 | -0.3169 | 0.2503 | -0.2301 | 0.8180 |
| DAB2IP | 0.9544 | -0.0467 | -0.4570 | 0.3637 | -0.2228 | 0.8237 |
| MMP3 | 0.9371 | -0.0650 | -0.6565 | 0.5266 | -0.2152 | 0.8296 |
| TGFBR1 | 1.0328 | 0.0322 | -0.2695 | 0.3340 | 0.2094 | 0.8341 |
| F2RL2 | 1.0424 | 0.0415 | -0.3510 | 0.4341 | 0.2074 | 0.8357 |
| CD63 | 0.9378 | -0.0642 | -0.6809 | 0.5526 | -0.2040 | 0.8384 |
| TNFSF15 | 1.0320 | 0.0315 | -0.2856 | 0.3485 | 0.1945 | 0.8458 |
| CCL19 | 1.0123 | 0.0122 | -0.1121 | 0.1365 | 0.1922 | 0.8476 |
| NOTCH3 | 1.0398 | 0.0390 | -0.3615 | 0.4395 | 0.1911 | 0.8485 |
| SHH | 0.9436 | -0.0580 | -0.6680 | 0.5519 | -0.1865 | 0.8521 |
| MUC4 | 0.9443 | -0.0573 | -0.6665 | 0.5519 | -0.1844 | 0.8537 |
| FGF19 | 0.9239 | -0.0792 | -0.9643 | 0.8059 | -0.1754 | 0.8608 |
| CCL2 | 1.0207 | 0.0205 | -0.2088 | 0.2498 | 0.1752 | 0.8609 |
| EPS8 | 0.9743 | -0.0260 | -0.3308 | 0.2788 | -0.1674 | 0.8671 |
| CDH11 | 1.0176 | 0.0174 | -0.1916 | 0.2265 | 0.1634 | 0.8702 |
| KLK6 | 1.0132 | 0.0131 | -0.1452 | 0.1715 | 0.1626 | 0.8708 |
| UCP2 | 1.0239 | 0.0236 | -0.2842 | 0.3314 | 0.1503 | 0.8805 |
| MIR34A | 0.9740 | -0.0264 | -0.3801 | 0.3273 | -0.1462 | 0.8838 |
| ROS1 | 0.9462 | -0.0553 | -0.8042 | 0.6935 | -0.1448 | 0.8849 |
| CCND1 | 0.9723 | -0.0281 | -0.4114 | 0.3552 | -0.1437 | 0.8857 |
| PHLDA2 | 1.0184 | 0.0182 | -0.2389 | 0.2754 | 0.1390 | 0.8895 |
| FGF1 | 0.9764 | -0.0239 | -0.4074 | 0.3595 | -0.1223 | 0.9027 |
| HK2 | 0.9810 | -0.0192 | -0.3566 | 0.3182 | -0.1115 | 0.9112 |
| CDH2 | 0.9913 | -0.0088 | -0.1691 | 0.1515 | -0.1072 | 0.9147 |
| PTX3 | 0.9733 | -0.0271 | -0.5360 | 0.4818 | -0.1044 | 0.9168 |
| COL8A1 | 0.9845 | -0.0156 | -0.3151 | 0.2839 | -0.1022 | 0.9186 |
| AJAP1 | 1.0201 | 0.0199 | -0.3693 | 0.4091 | 0.1003 | 0.9201 |
| SPOCK1 | 0.9911 | -0.0090 | -0.1903 | 0.1724 | -0.0970 | 0.9228 |
| ANPEP | 1.0134 | 0.0133 | -0.2754 | 0.3020 | 0.0903 | 0.9280 |
| ALK | 0.9853 | -0.0148 | -0.3425 | 0.3129 | -0.0887 | 0.9293 |
| IRF8 | 1.0130 | 0.0129 | -0.2752 | 0.3010 | 0.0877 | 0.9301 |
| AHR | 0.9877 | -0.0124 | -0.3134 | 0.2886 | -0.0806 | 0.9358 |
| IL17RD | 1.0124 | 0.0123 | -0.3109 | 0.3355 | 0.0746 | 0.9405 |
| COL8A2 | 1.0070 | 0.0069 | -0.1835 | 0.1974 | 0.0714 | 0.9431 |
| FAS | 0.9887 | -0.0114 | -0.3805 | 0.3577 | -0.0605 | 0.9518 |
| PROM1 | 1.0041 | 0.0041 | -0.2990 | 0.3072 | 0.0266 | 0.9788 |
| LHX2 | 0.9978 | -0.0022 | -0.2958 | 0.2914 | -0.0149 | 0.9881 |
| DLX4 | 0.9973 | -0.0027 | -0.5117 | 0.5063 | -0.0104 | 0.9917 |
| BHLHE40 | 1.0012 | 0.0012 | -0.2706 | 0.2730 | 0.0089 | 0.9929 |
| NRP2 | 0.9999 | -0.0001 | -0.2525 | 0.2523 | -0.0008 | 0.9993 |
